# STopover captures spatial colocalization and interaction in the tumor microenvironment using topological analysis in spatial transcriptomics data

**DOI:** 10.1186/s13073-025-01457-1

**Published:** 2025-04-01

**Authors:** Sungwoo Bae, Hyekyoung Lee, Kwon Joong Na, Dong Soo Lee, Hongyoon Choi, Young Tae Kim

**Affiliations:** 1 Institute of Radiation Medicine, Medical Research Center, Seoul National University, Seoul, Republic of Korea; 2 Portrai, Inc., Seoul, Republic of Korea; 3 Department of Nuclear Medicine, Seoul National University Hospital, Seoul, Republic of Korea; 4 Department of Thoracic and Cardiovascular Surgery, Seoul National University Hospital, 101 Daehak-Ro, Jongno-Gu, Seoul, Republic of Korea 03080; 5 Medical Science and Engineering, School of Convergence Science and Technology, POSTECH, Pohang, Republic of Korea; 6 Department of Nuclear Medicine, Seoul National University College of Medicine, Daehak-Ro, Jongno-Gu, 101 Seoul03080, , Republic of Korea

**Keywords:** Spatially resolved transcriptomics, Topological analysis, Connected components, Colocalization, Lung cancer, Breast cancer, Tumor microenvironment

## Abstract

Unraveling the spatial configuration of the tumor microenvironment (TME) is crucial for elucidating tumor-immune interactions based on immuno-oncology. We present STopover, a novel approach utilizing spatially resolved transcriptomics (SRT) data and topological analysis to investigate the TME. By gradually lowering the feature threshold, connected components (CCs) are extracted based on spatial distance and persistence, with Jaccard indices quantifying their spatial overlap, and transcriptomic profiles are permutated to assess statistical significance. Applied to lung and breast cancer SRT, STopover revealed immune and stromal cell infiltration patterns, predicted key cell–cell communication, and identified relevant regions, shedding light on cancer pathophysiology (URL: https://github.com/bsungwoo/STopover).

**Supplementary Information:**

The online version contains supplementary material available at 10.1186/s13073-025-01457-1.

## Background

One of the essential characteristics of cancer progression is acquiring an immune escape mechanism in the tumor microenvironment (TME) [1]. The heterogeneous cells in the TME constitute the landscape to evade the immune system, which has been regarded as a target for immunotherapy [2]. The most widely investigated mechanism is the immune checkpoint, which serves to protect normal cells from cytotoxic immune cells. Enhancement of either axis of immune checkpoints eventually leads to impaired function or apoptosis of cytotoxic immune cells such as CD8 + T cells [3, 4]. Alternatively, MHC class I expression, which is responsible for the antigen presentation of cancer cells, is reduced [5], or cancer interacts with cancer-associated fibroblasts (CAFs) and alters the microenvironment such that immune cells have more difficulty suppressing tumor growth [6]. Cancer immunotherapy targeting these mechanisms is gaining a lot of attention lately, particularly with regard to targeting PD-1, PD-L1, and CTLA-4. Multiple preclinical studies and clinical trials have demonstrated that immunotherapy targeting various mechanisms in the TME combined with conventional chemotherapy results in effective tumor suppression [7].

However, not all patients respond well to immunotherapy, even though predictive biomarkers for immune checkpoint inhibitors (ICIs), such as PD-L1 expression, microsatellite instability, and tumor mutational burden, have been used in the clinic [4]. As a spatial feature of the TME is related to the immunotherapy response, the infiltration pattern of immune cells in the tumor is regarded as a key to characterizing immune status [8]. In the case of an immune-excluded or immune-desert tumor where immune cells do not penetrate the tumor, the response to immunotherapy is bound to be poor. Moreover, not only tumor and immune cells but also stromal components in the tumor microenvironment (TME) influence immune cell action on cancer cells [9]. Given that the therapeutic response of ICI is determined by complex intercellular interactions, including lymphocytes, myeloid cells, and fibroblasts in the TME, a method that simultaneously analyzes the spatial configuration of multiple cell types is needed.

With the introduction of spatially resolved transcriptomics (SRT), which acquires the location of RNAs and their quantitative expression in the tissue, the spatial characteristics of cells can be comprehensively analyzed [10]. Because this technique provides whole gene expression data with spatial information, it has the potential to comprehensively analyze the spatial configuration and complex interactions of various cells in the TME. Several computational methods have been developed that utilize SRT to capture cell infiltration patterns in the TME and to estimate the key ligand-receptors of cell–cell interactions. However, they cannot localize the important subregions of cell infiltration and molecular interaction across all cell types present in the cancer tissue. Also, they do not provide a common analytical framework that encompasses both barcode and image-based SRT platforms.

Here, we propose a method, STopover, which applies topological analysis to SRT to extract colocalization patterns between cell types in cancer tissue and to predict the degree of intercellular interaction. Our suggested new approach provides the extent of topological overlap between various cells, such as tumor and immune cells, and presents an estimate of the spatial interaction between cells. STopover can provide quantitative information on spatial interactions in the TME beyond spatial gene expression or cell enrichment.

## Methods

### Dataset characteristics and details

#### Simulation dataset

The simulation dataset was created to test the usefulness of STopover in capturing locally active subregions where one of the features has a low value while the other has a high value. For simplification, the spatial map of two features, tumor and immune cells, was created by adding a trimmed 2D Gaussian function multiple times to the 100 by 100 grid with zero background.

$$f\left(x, y\right)=\left\{\begin{array}{c}M{e}^{-\frac{({x-{c}_{x})}^{2}+{\left(y-{c}_{y}\right)}^{2}}{2{\sigma }^{2}}}, ({x-{c}_{x})}^{2}+{\left(y-{c}_{y}\right)}^{2}<(2{\sigma )}^{2}\\ 0, ({x-{c}_{x})}^{2}+{\left(y-{c}_{y}\right)}^{2}\ge (2{\sigma )}^{2}\end{array}\right.$$

*f(x,y): trimmed 2D Gaussian function for the simulated feature value, M: maximum feature value in the center, c*_*x*_*: x coordinate of the center, c*_*y*_*: y coordinate of the center, σ: standard deviation.*

The input values, M, c_x_, c_y_, and σ, were (8, 30, 30, 20), (8, 60, 70, 10), (2, 70, 20, 10), and (1, 90, 90, 10) in tumor cells and (4, 95, 85, 5), (2, 20, 20, 5), (2,70, 20, 5), (1, 70, 70, 5), and (1, 85, 95, 5) in immune cells. Then, the noise was generated by random sampling from a Gaussian distribution (mean: 1 and standard deviation: 0) and multiplying the sampled values by 0.01. Last, to mimic the noise in the SRT dataset, two independently generated noise samples were added to the 2D grid of tumor and immune cells, and the negative values were replaced with zero.

#### Barcode-based SRT of human lung cancer

Eleven lung adenocarcinoma (ADC) tissue samples were acquired from seven patients who were initially diagnosed with lung cancer and underwent surgical resection. The study protocol was reviewed and approved by the Institutional Review Board of Seoul National University (application number: H-2009–081–1158). The patient number and the tissue number were used to name the samples. For example, the second tissue obtained from patient number 18 was named *spa18ca02*. The samples were divided into two groups based on the PD-L1 expression level. The PD-L1 low group is composed of *spa01ca01*, *spa01ca02*, *spa02ca01*, *spa02ca02*, *spa06ca01*, and *spa10ca01* with PD-L1 expression of 0. The PD-L1 high group expression group is composed of *spa16ca01*, *spa17ca01*, *spa17ca02*, *spa18ca01*, and *spa18ca02* with PD-L1 expression above 80%. The acquired samples were embedded in an optimal cutting temperature (OCT) compound, cryosectioned, and processed to generate Visium spatial transcriptomic datasets. The diameter of the spot, the basic unit of the Visium spatial transcriptome, was 55 µm, and the distance between the spots was 110 µm. The number of spots and genes utilized for the analysis, patient history, and histological information of the samples are summarized in Additional file 2: Table S1 [11]. The cell type composition in the spot was inferred based on the reference single-cell data obtained from human lung cancer tissue [12, 13]. The cell types were classified into 7 large categories (epithelial cells, fibroblasts, endothelial cells, myeloid cells, MAST cells, B lymphocytes, and NK/T cells) and 47 cell subtypes. For example, the NK/T cells were divided into NK cells, naïve CD4 + T, CD4 + Th, CD8 + /CD4 + mixed Th, exhausted Tfh, Treg, naïve CD8 + T, cytotoxic CD8 + T, exhausted CD8 + T, CD8 low T cells, and T lymphocytes_ns.

#### Image-based SRT of human lung cancer

The publicly available CosMx SMI dataset [14], which is one of the image-based SRT platforms, was utilized for STopover analysis. The dataset was a formalin-fixed paraffin-embedded (FFPE) sample of non-small cell lung cancer [15]. The RNA was captured across 30 field of views (FOVs), and the size of each FOV was 0.985 by 0.657 mm. The margin of the cells was segmented based on the immunofluorescence image, and the cell-level count matrix was constructed. The coordinate of the transcript, cell assignment data of each transcript, cell-level expression profiles, and cell metadata files were utilized for the STopover analysis. The numbers of transcripts and cells were 30,370,769 and 100,149, respectively. Among the transcripts, 37,226,610 could be assigned to the cell and corresponded to 960 gene symbols and 20 negative probes, which do not correspond with any sequence. The cell type annotation of the segmented cells was performed with identical lung cancer single-cell data [12, 13], which were used for cell type decomposition in barcode-based SRT.

#### Barcode-based SRT of human breast cancer

The publicly available Visium spatial transcriptomics and single-cell transcriptomics data from four breast cancer patients (CID4290, CID4465, CID44971, and CID4535) were used for analysis [16–18]. The histological type of the tumors was invasive ductal carcinoma (IDC), except for CID4535, which was invasive lobular carcinoma (ILC). CID4290 and CID4535 were classified as ER + tumors, while CID4465 and CID44971 were classified as TNBCs. All of the patients had not received any treatment prior to spatial and single-cell transcriptomic data acquisition. The spatial composition of cell types in the breast cancer tissues was predicted based on the corresponding single-cell data obtained from the same patient. All the cells were classified into nine major cell types as described in the paper [16]: cancer epithelial cells, normal epithelial cells, cancer-associated fibroblasts (CAFs), endothelial cells, perivascular-like cells (PVLs), myeloid cells, T cells, B cells, and plasmablasts. Plasmablasts were not present in the single-cell data for patient CID4290, and therefore, the fraction of plasmablasts was considered 0 in all spots.

#### Preprocessing barcode- and image-based SRT

The preprocessing steps for the SRT datasets are mainly based on Scanpy (ver. 1.9.1) [19] running on Python (ver. 3.8). First, the spot-level count matrix of barcode-based SRT is processed for downstream analysis. The RNA count of each spot is normalized such that the total count became 10,000 and log-transformed [${\text {ln (normalized count +1)}}$]. Cell type decomposition is performed by applying either CellDART [20] or Cell2location algorithm [21] to each Visium spatial transcriptomic dataset based on the reference single-cell dataset. The selection of the two methods is based on a study comparing several cell type deconvolution methods, which showed that the performance of CellDART and Cell2location is the highest [20]. As a result, a spatial composition map of all cell types in the lung cancer tissue is generated. The spatial distribution of cell types is utilized to calculate cell–cell colocalization patterns. Additionally, the spatial expression of LR pairs is adopted to estimate spatial cell–cell communication.

Second, image-based SRT data are processed to create a grid-based count matrix, which enabled analysis of the data similar to the barcode-based SRT. The whole FOV, including all 30 small FOVs, is divided into 100 by 100 grids based on the outermost coordinate of the transcript (Additional file 1: Fig. S1a). The size of the unit grid is approximately 49.2 by 39.4 µm, which is similar to the diameter of the spot from Visium spatial transcriptomics. The grid-level RNA count is normalized to fit the total count in each cell to 1000 and log-transformed. The fraction of the cell assigned to each grid is defined by the ratio of total RNA counts in a portion of the cell belonging to the grid to total RNA counts in the cell (Additional file 1: Fig. S1b). Then, the cell-level count matrix is utilized to annotate the cells based on the reference single-cell data with the “Ingest” algorithm provided by Scanpy (scanpy.tl.ingest) [19] or “TACCO” algorithm [22] (Additional file 1: Fig. S1c). The tool maps the reference single-cell expression data into the spatial single-cell embedding using principal component analysis and the *k*-nearest neighbor search method suggested in the uniform manifold approximation and embedding algorithm [23]. After the annotation of cell types, the grid-level abundance of cell types is calculated by summing the fraction of all cells in the grid corresponding to the cell type (Additional file 1: Fig. S1d). Of note, cell type-specific expression is calculated by extracting the transcripts belonging to the specific cell types and performing log-transformation of the summed normalized count. Finally, a spatial map of cell type abundance is utilized to calculate spatial cell–cell colocalization and grid-level log-normalized counts are applied to compute spatial cell–cell interactions.

#### STopover: extracting colocalized patterns of a feature pair

**Fig. 1:**
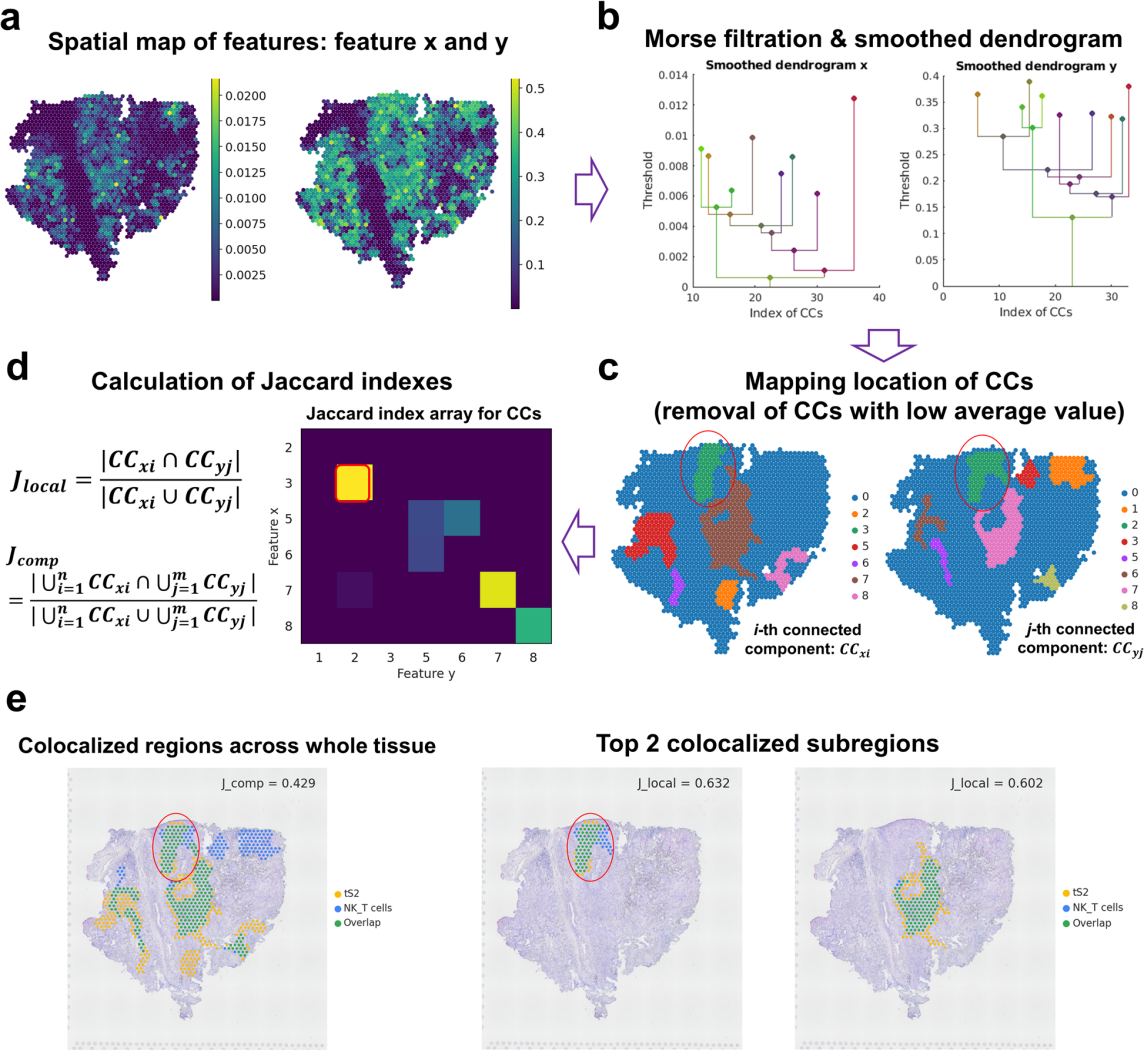
Schematic image of STopover. STopover is a tool that utilizes spatially resolved transcriptomics (SRT) and applies topological analysis to extract colocalization patterns between cell types and estimate spatial cell–cell interaction in the tumor microenvironment. **a **The spatial map of features such as cell fraction or gene expression in each spot was utilized. The spatial distribution of cell types is given as inputs to the STopover model when analyzing cell–cell colocalization. The LR pairs from the CellTalkDB database [26] are provided as inputs when estimating spatial cell–cell communication mediated by ligand-receptor (LR) interactions. **b, c** By utilizing Morse filtration and dendrogram smoothing processes, the key locations of the overlapping spatial domain are extracted as connected components (CCs). **d** After removing CCs with low average feature values, the Jaccard indices are calculated for every CC pair between the two features and named *J*_local_. **e** The CC pairs with a large *J*_local_ indicate important tissue subregions where the two features are highly colocalized. Additionally, all CCs from each feature are aggregated, and the Jaccard index between the two aggregated CCs is calculated and named *J*_comp_. *J*_comp_ measures the extent of spatial overlap of the two features on a global scale

STopover applies Morse filtration and the dendrogram smoothing algorithm [24], one of the topological analysis methods, to extract connected components (CCs) from the given feature pair and calculate the overlap between the CC pairs (Fig. 1 and Additional file 1: Fig. S2). First, to reduce the intrinsic sparsity of features in SRT, a Gaussian smoothing filter is applied to the spatial feature map. The filter with a full-width half maximum (FWHM) of 2.5 times the unit central distance between spots or grids is applied for smoothing. This parameter ensures a balance between smoothing the data and retaining significant spatial features, facilitating more accurate detection of spatial overlaps. Next, CCs are calculated for all thresholds while lowering the value from the highest feature value in the tissue to the lowest value using the algorithm suggested by NetworkX [25]. The existing CCs from the higher threshold were gradually merged or new CCs are defined based on the spatial distance from the newly added spot or grid to the existing CCs. This hierarchical clustering process is summarized with a dendrogram. Each vertical bar in the dendrogram represents the start and end of threshold values that a certain CC is continuously observed, and each horizontal bar links the existing CCs (child) with merged or new CCs (parent) from the lower threshold value. Then, the dendrogram is smoothed such that the hierarchical structure of the spatial feature map can be simplified. The vertical bars are selected in the order of the longest length to the shortest length, and if the connected children CCs of the selected bar have a smaller size than the minimum size of CCs, then their elements are removed and aggregated to the parent. Additionally, the start point of the vertical bars is updated to have the maximum feature value in the CC. The process is iterated until all of the vertical bars are selected and the uppermost bars of the dendrogram, reconfigured CCs, represent locally activated regions of the given feature. Next, the average feature value is computed within each CC region, and CCs, which are presumed to capture noise due to a low average value below a certain percentile, are deleted. Then, the Jaccard index is calculated for all possible pairs of reconfigured CCs from the two features and named *J*_local_. The CC pair with a high *J*_local_ value indicates the tissue subregion where the extent of overlap between two features is high. Additionally, all CCs of each feature are aggregated, and the Jaccard index between the two aggregated CCs is calculated and named *J*_comp_. The Jaccard index is calculated in two different ways: using set-based and weighted methods. The set-based method binarizes the patterns of the feature pair as CCs and finds the overlap between the two features. The Jaccard index from the set-based method is computed by dividing the size of the intersection of two CCs by the union of the CCs. On the other hand, the weighted method considers the feature values inside CCs. The index from the weighted method is calculated by scaling the feature values between 0 and 1 in the whole tissue and then dividing the sum of the minimum scaled feature values inside CCs between the two features by the sum of the maximum scaled values. The formulas below provide further details on how the Jaccard indices are calculated.

$${J}_{S,local\left(i,j\right)}=\frac{|C{C}_{xi}\cap C{C}_{yj}|}{|C{C}_{xi}\cup C{C}_{yj}|}$$

$${J}_{S,comp}=\frac{|{\bigcup }_{i=1}^{n}C{C}_{xi}\cap {\bigcup }_{j=1}^{m}C{C}_{yj}|}{|{\bigcup }_{i=1}^{n}C{C}_{xi}\cup {\bigcup }_{j=1}^{m}C{C}_{yj}|}$$

$${J}_{W,local\left(i,j\right)}=\frac{{\sum }_{s\in C{C}_{xi}\cap C{C}_{yi}}\text{min}({x}_{s},{y}_{s}) }{{\sum }_{s\in C{C}_{xi}\cup C{C}_{yi}}\text{max}({x}_{s}, {y}_{s})}$$

$${J}_{W,comp}=\frac{{\sum }_{s\in [{\bigcup }_{i=1}^{n}C{C}_{xi}\cap {\bigcup }_{j=1}^{m}C{C}_{yj}]}\text{min}({x}_{s},{y}_{s}) }{{\sum }_{s\in [{\bigcup }_{i=1}^{n}C{C}_{xi}\cup {\bigcup }_{j=1}^{m}C{C}_{yj}]}\text{max}({x}_{s}, {y}_{s})}$$

*J*_*S*_: Jaccard index calculated by the set-based method, *Jw*: Jaccard index calculated by the weighted method, *CC*_*xi*_: set including spots or grids belonging to the *i*th connected component of feature *x*, *CC*_*yj*_: set including spots or grids belonging to the *j*th connected component of feature *y*, *x*_*s*_: scaled value of feature x in the spot s, *y*_*s*_: scaled value of feature *y* in the spot *s* (if the spot is not included in *CC*_*xi*_, then *x*_*s*_ is 0, and if not included in *CC*_*yi*_ then *y*_*s*_ is 0).

The main parameters in the models are the minimum size of CCs (${\text{s}}_{\text{min}}$) expressed as the number of spots or grids and the percentile threshold to remove the CCs with low average feature values (${\text{p}}_{\text{t}}$). Increasing both ${\text{s}}_{\text{min}}$ and ${\text{p}}_{\text{t}}$ is expected to remove the noise. However, if ${\text{s}}_{\text{min}}$ is too large, then too many CCs are aggregated, and if ${\text{p}}_{\text{t}}$ is too large, then many CCs unrelated to noise will disappear, which impairs the performance of the STopover. The spatial expression patterns of genes with low average expression exhibit sparse distributions, which may result in a different optimal parameter range compared to dense features like genes with high expression or cell type proportion. To evaluate the optimal parameter range for different feature types, both sparse and dense distributions, we conducted tests to observe how *J*_comp_ values change across various parameters (Additional file 1: Fig. S3). First, for the dense distributions, *J*_comp_ was measured between tS2 (tumor cell type) and T cells in barcode-based ST data of the lung cancer across different ${\text{s}}_{\text{min}}$ and ${\text{p}}_{\text{t}}$ values (Additional file 1: Fig. S3a-c). The two cell types were chosen because the range of *J*_comp_ value is wide across the 11 lung cancer tissues, making it easy to investigate the influence of the parameters on *J*_comp_. For the sparse distributions, *J*_comp_ was computed between one of the ligand-receptor pairs related to the T-cell activation (*CD274*-*PDCD1*), which shows abundant zero values in the spots (Additional file 1: Fig. S3d, e). During the evaluation, FWHM of the Gaussian smoothing filter was fixed to 2.5 times the central distance between spots or grids to minimize the effects of noise and variable changes of the features (genes or cell types) within small subregions. When ${\text{s}}_{\text{min}}$ and ${\text{p}}_{\text{t}}$ were set to 20 and 30, *J*_comp_ remained most stable when ${\text{s}}_{\text{min}}$ was set to 20 and effectively highlighted differences between tissues when ${\text{p}}_{\text{t}}$ was set to 30. The ${\text{s}}_{\text{min}}$ of 20 corresponds to diameters of approximately 479.3 and 295.3 µm in circular CCs of barcode- and image-based SRTs, which are the reference sizes for the smallest regions where local interactions occur. Because the spot in the barcode-based SRT and the grid in the image-based SRT are similar in size, the optimal values of ${\text{s}}_{\text{min}}$ and ${\text{p}}_{\text{t}}$ in both datasets could be shared. In the case of the simulation dataset, the optimal ${\text{s}}_{\text{min}}$ and ${\text{p}}_{\text{t}}$ values to reduce the noise and better represent the spatial feature map were 20 and 80, respectively (Additional file 1: Fig. S4).

#### STopover: estimating cell–cell interaction patterns in tumors

Cell–cell communication is estimated based on the assumption that cell–cell interactions mediated by LR interactions occur within a range of few spots or grids. CellTalkDB or Omnipath [26, 27], the curated LR database, is selected, and the spatial colocalization pattern of all LR expressions is searched. The LR pairs with a high colocalization score (*J*_comp_) are presumed to show high interaction in the tissue subregions represented by CCs. The most meaningful LRs are selected by filtering out those with a *J*_comp_ below the threshold of 0.200 (in some cases, 0.150). GO analysis [28, 29] is performed for the filtered ligand and receptor gene sets using gseapy (version 0.10.8) [30, 31] in Python or clusterProfiler (version 4.2.2) [32] in R. The overrepresented biological process terms are extracted to comprehend the functional role of the dominant LR interaction in the given tissue. Also, differential LR interaction analysis is performed to compare the strength of cell–cell communication between the two groups of tissues. The average *J*_comp_ values are calculated across the tissues within each group, and the fold change of *J*_comp_ is used to determine the degree of difference in LR interaction. When identifying differential LR interactions that are increased in group 2 compared to group 1, LR pairs with an average *J*_comp_ in group 2 greater than 0.200 and a fold change of *J*_comp_ greater than 2 are selected. For LR interaction that shows a decrease in group 2 compared to group 1, LR pairs with an average *J*_comp_ in group 1 greater than 0.200 and a fold change of *J*_comp_ less than 0.500 are selected.

To search cell type-specific LR interactions in barcode-based SRT, modified CCs are defined as intersecting subregions between CCs obtained from LR interaction analysis and the colocalized tissue domain of the two cell types extracted by STopover. *J*_local_ and *J*_comp_ are calculated between the modified CCs of the two features. In the case of image-based SRT, the cell type-specific (cell types A and B) log-normalized count is first computed, and spatial overlap patterns between ligand expression of cell type A and receptor expression of cell type B are extracted to predict spatial cell–cell interactions.

#### STopover: calculating the statistical significance of detected colocalization

There is no definitive threshold for deciding whether the observed spatial colocalization between a pair of features is statistically significant. To address this issue, whole transcriptome profiles in each spot or grid (spot: barcode-based SRT; grid: image-based SRT) were shuffled while keeping the spatial coordinates fixed, resulting in the loss of local distribution patterns. The *J*_comp_ between pairs of CCs was calculated in all 1000 cases of permutation, generating a null distribution of *J*_comp_. The proportion of cases where *J*_comp_ from the null distribution exceeded that from the original SRT was defined as the *p*-value. *P*-values less than 0.05 were considered statistically significant.

#### Comparison with other methods for investigating spatial heterogeneity of tumors

Other computational tools that analyze tumor heterogeneity using SRT were applied to lung cancer tissues and compared with STopover. The first tool, ATHENA, builds a graph based on the spatial distance between cells in image-based SRT [33]. It calculates infiltration scores to determine the overall extent of tumor infiltration and identify subregions of cancer where immune cells are infiltrated. In ATHENA, cells are treated as nodes, and cells within a distance of approximately half the size of the grid defined in the STopover analysis are connected with edges. The cell type annotations obtained during the preprocessing of the CosMx SMI data were also used in the ATHENA analysis. The infiltration score for a particular cell type A is calculated as the ratio of the number of connections between the tumor and A cells to the number of connections between A cells only. Global scores are obtained from a graph that encompasses the entire tissue, while local scores are obtained from a subgraph containing only one-hop neighbors. The second tool, Squidpy [34, 35], calculates the LR interaction in image-based SRT by randomly shuffling cell labels and calculating the mean of the average expression of the receptor in the receiver cell cluster and the ligand in the sender cell cluster. The null distribution generated by the random shuffling was used as a reference to calculate the statistical significance of the LR interaction. In addition to the LR interaction, the neighborhood enrichment score between cell type X and Y is calculated by counting the pair of proximal cells belonging to X and Y. Then, the cell labels were randomly shuffled while maintaining the cell connectivity based on spatial distance, and the *Z*-score was calculated for statistical evaluation. The default parameters suggested in the user guide were applied for the above analyses. The third tool, spatialGE, segments cancer cell regions in the tissue and computes spatial heterogeneity scores from the high-resolution spatial map of each cell type using barcode-based SRT [36]. The cell composition predicted by CellDART was directly applied to spatialGE, and high-resolution spatial maps of the tumor and other cell types were created and visualized to assess the immune phenotype of the given cancer tissue. The fourth tool, SpaCET ranks the degree of cell–cell colocalization in barcode-based SRT using Spearman’s correlation coefficient and finds the colocalized regions by selecting the spots with cell fraction within the top 15% [37]. In addition, it calculates the top ligand-receptor interaction of the cell type pair by calculating correlation coefficient of ligand and receptor expression inside colocalized region of the cell type pair. The cell fraction predicted from reference single-cell dataset was used and the cell–cell colocalization results were compared between SpaCET and STopover. The final tool, stLearn, calculates cell type diversity and ligand-receptor coexpression scores in each spot of the barcode-based SRT and then incorporates both scores to capture areas with a high probability of intercellular interaction [38].

## Results

### STopover captures colocalized regions of a feature pair in a simulation dataset

STopover was designed to quantify the topological colocalization of two given features using Morse filtration (Fig. 1 and Additional file 1: Fig. S2). The method gradually reduces the threshold of features in tissue regions to identify CCs based on spatial proximity, and after refining and analyzing these CCs with set-based and weighted approaches, it visualizes the spatial interactions between different cell types using both barcode- and image-based SRT data. However, there is no clear threshold exists for determining whether spatial colocalization scores observed between CCs indicate significant colocalization. To address this issue, we simulated a scenario in which local distribution patterns of the features were lost and compared colocalization scores from this simulation to the original SRT data. By shuffling transcriptomic profiles and considering feature values in the tissue unit region (spots or grids) as separate observations, a null distribution of spatial overlap scores was generated. The significance of colocalization, represented by *p*-values, was calculated as the proportion of shuffled scores in the null distribution that exceeded the original score.

**Fig. 2:**
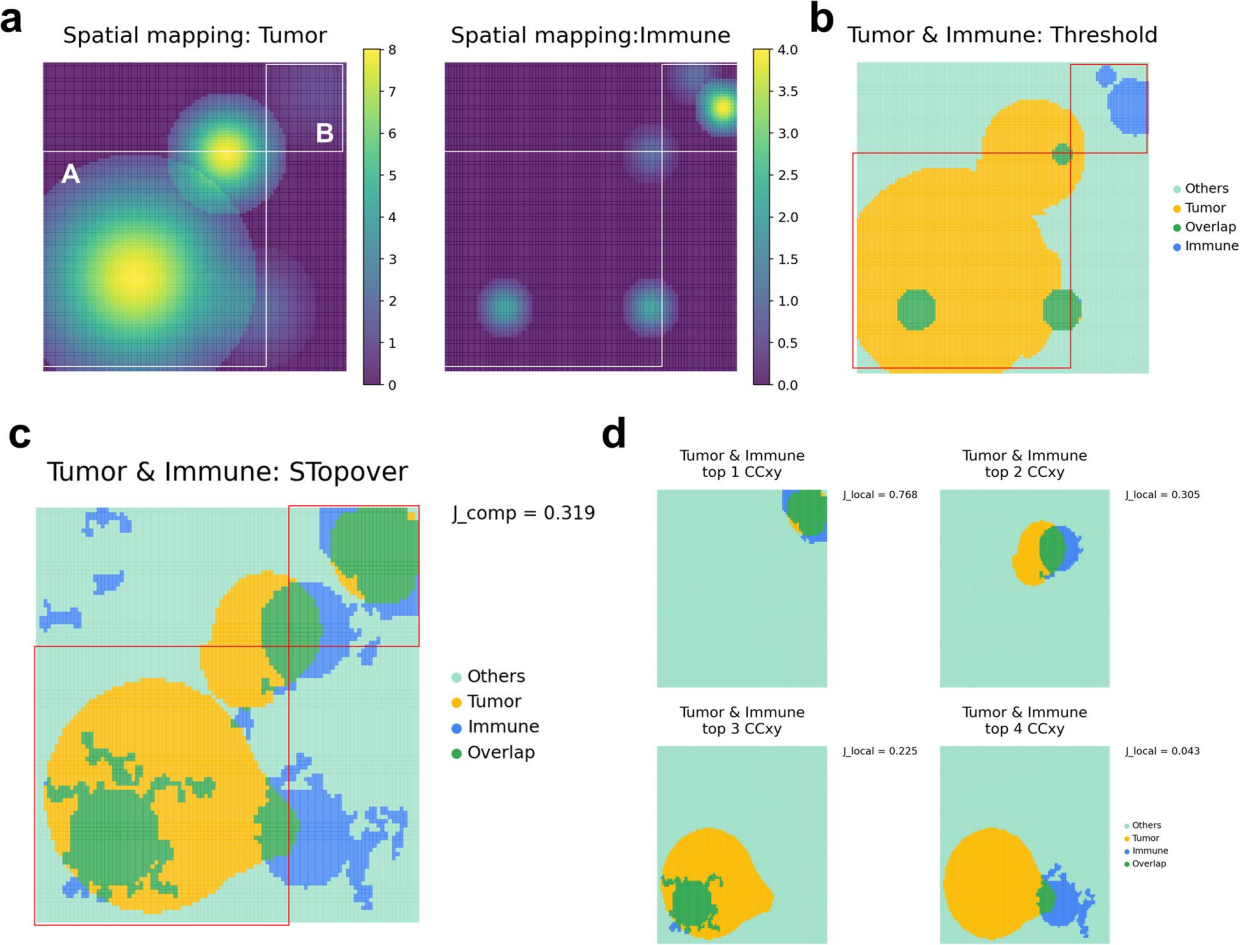
STopover reveals a subregional colocalization pattern in the simulation dataset. A simulation dataset was created to examine the ability of STopover to capture small but highly colocalized subregions. Trimmed 2D Gaussian functions were applied multiple times to a 100 by 100 grid, and the activity of tumor and immune cells was simulated. **a** The spatial maps of tumor and immune cell activity were visualized with a colormap. **b** A threshold-based approach was applied, and the regions where the activity was above 20% of the maximum were filtered to segment the key regions of the tumor (yellow) and immune cells (blue). Then, spatially overlapping domains between the two cell types are highlighted in green. **c** STopover was applied to segment the main patterns of tumor (yellow) and immune cell (blue) activity as CCs. The CCs for the tumor (yellow) and immune cells (blue) and the intersecting subregions (green) between the two aggregated CCs were visualized on the grid. The set-based Jaccard index was computed between these combined CCs (*J*_comp_). **d** The top 4 CC pairs between tumor and immune cells showing the highest spatial overlap represented by the *J*_local_ score were visualized. The CCs from the tumor (yellow), immune cells (blue), and intersecting subregions (green) were visualized on the grid. The set-based Jaccard indexes were computed for each CC pair (*J*_local_). Overall, STopover was superior to the conventional threshold-based method in capturing locally active subregions where tumor cell activity is low but immune cell activity is high (Region B).

First, the utility of STopover, which extracts topographically overlapping tissue domains between the cell type pairs, was examined in a simulation dataset and compared with the conventional threshold-based approach. As an example of the threshold-based approach to delineating the overlapping tissue regions of the two cell types, a threshold filter that removes the value below 20% of the maximum was applied to segment the main tissue domain where the two cell types are colocalized. The threshold-based method could capture cancer and immune cell overlap in the region where both cell types showed high abundance (Region A in Fig. 2a, b). However, that method could not accurately delineate the key location where cancer cells are scarce while immune cells are highly abundant (Region B in Fig. 2a, b). Thus, the arbitrary threshold to localize cell type-rich regions can miss the locally active regions of cancer and immune interactions. In contrast, STopover could precisely capture the colocalized tissue domains between cancer and immune cells (Region B in Fig. 2a, c). Moreover, as quantitative information, STopover could rank the degree of overlap (*J*_local_) between the CCs of cancer and immune cells with the Jaccard index and indicate subregions where high cancer-immune interactions are expected (Fig. 2d). There were no significant differences between the set-based and weighted methods of the Jaccard index in ranking the top locations of the tumor-immune colocalization (Fig. 2 and Additional file 1: Fig. S5). Jaccard indexes were consistently lower in the weighted method, and the difference between *J*_local_ values in the top first and second subregions was larger (Fig. 2d and Additional file 1: Fig. S5b). This implies that while the weighted method considers the spatial patterns inside the captured subregions represented by CCs, noise in the features can result in an overall reduction in the Jaccard index.

### STopover reveals spatial overlap patterns between lung cancer cell types in barcode-based SRT

To assess the effectiveness of the method in cancer tissues, STopover was applied to the human lung ADC Visium data. First, reference single-cell data obtained from human lung cancer tissue [12] were integrated with spatial data using CellDART [20] to estimate the spatial distribution of the major cell types. Then, the localization patterns of the tumor cell type (‘tS2’), which is related to lung ADC progression [12], and other main cell types (‘Fibroblasts’, ‘Endothelial cells’, ‘Myeloid cells’, ‘MAST cells’, ‘B lymphocytes’, and ‘NK_T cells’) were extracted by STopover. The composite overlap score was calculated by measuring the set-based Jaccard index between the combined CC aggregates between the two cell types (*J*_comp_) (Fig. 1). In addition, the local overlap score (*J*_local_) was computed by measuring the set-based Jaccard index between every CC pair of the two cell types. *J*_comp_ represents the degree of global overlap between the two cell types, while *J*_local_ shows tissue subregions where the cell distribution is highly colocalized.

**Fig. 3:**
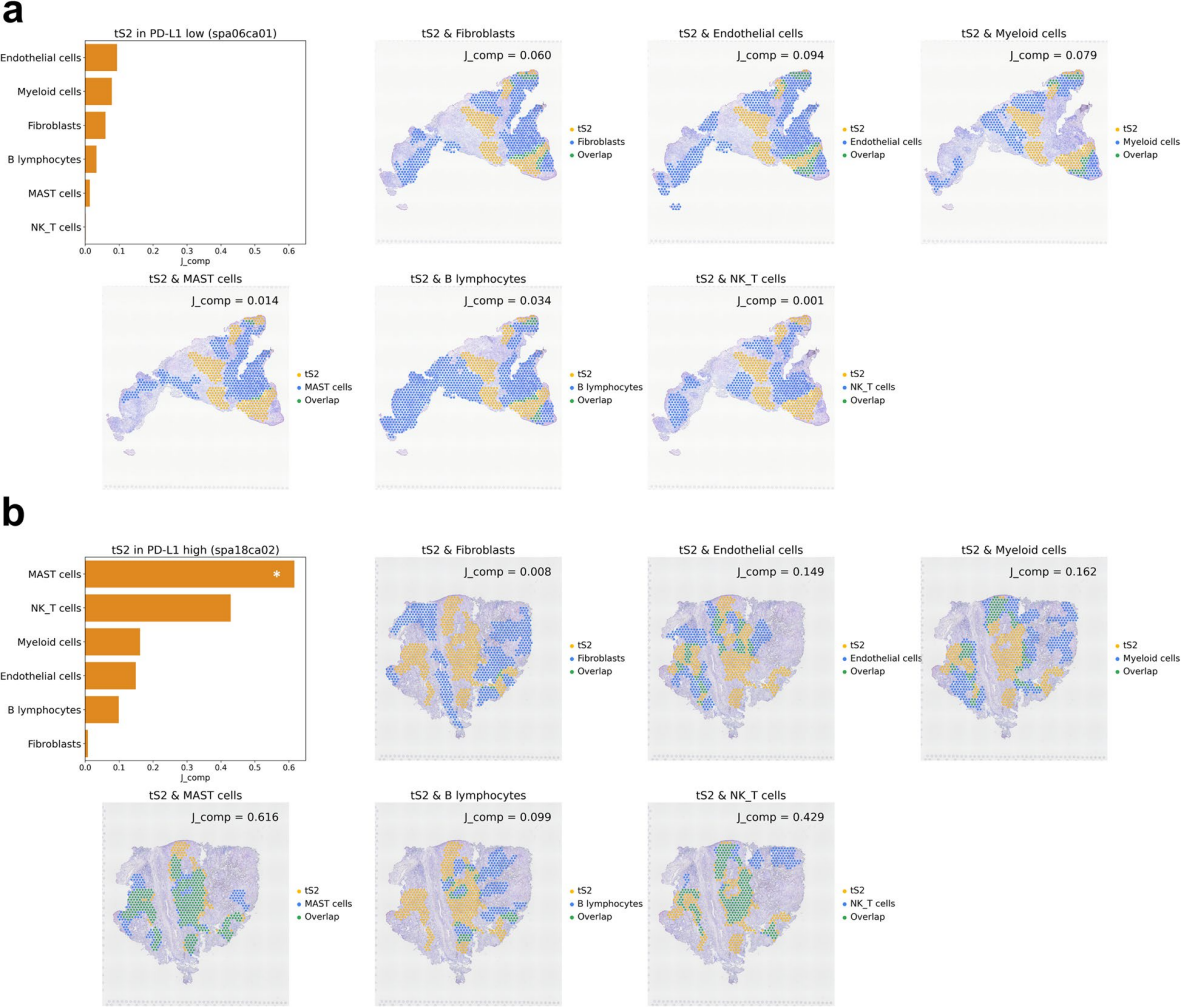
STopover explains the spatial configuration of the TME in lung cancer tissues using barcode-based SRT. STopover was applied to barcode-based SRT of lung cancer tissues with high PD-L1 expression (*spa06ca01*, 0%) and low PD-L1 expression (*spa18ca02*, 100%). The spatial colocalization patterns between tS2, one of the cancer epithelial subtypes associated with the progression of cancer, and other main cell types (fibroblasts, endothelial cells, myeloid cells, MAST cells, B lymphocytes, and NK/T cells) were investigated in both tissues. **a** The set-based *J*_comp_ values between the tumor cell type (tS2) and the other cell types in *spa06ca01* tissue were visualized as a bar plot in the top left corner of the plot. Additionally, the aggregated CCs for tS2 (yellow) and other main cell types (blue) were mapped to the tissue, and the intersecting tissue domain was highlighted in green. **b** The set-based *J*_comp_ values for tS2 and other cell types in *spa18ca02* tissue were also visualized with a barplot, and the CC locations were mapped to the tissue. In both barplots in **a** and **b**, statistically significant colocalization between tS2 and other cell types is visualized as a white asterisk (*p*<0.05). The two selected PD-L1 high and low tissues showed converse patterns of cell infiltration in the tumor, and the extent of infiltration could be measured as *J*_comp_

Two representative slides were selected among 11 tissue sections to evaluate whether STopover accurately extracts the spatial overlap patterns of cells in the TME. One of the tissues (‘*spa06ca01*^*’*^) had low PD-L1 expression (0%) and showed immune-excluded patterns by visual inspection (Additional file 1: Fig. 6a). The other tissue (‘*spa18ca02*’^*’*^) had high PD-L1 expression (100%) and presented immune-inflamed patterns (Additional file 1: Fig. S6b). When the spatial distribution of cell types was extracted as CCs and mapped to the tissues, the CC distribution was concordant with spatial cell type patterns (Fig. 3 and Fig. S6). The spatial overlap pattern between cell types in two lung ADC samples exhibited disparate patterns of tumor, immune, and stromal distribution (Fig. 3). In the PD-L1 low tissue, the spatial patterns of the tumor cell type (tS2) and immune cells, including MAST and NK/T cells, were highly exclusive (Fig. 3a). Accordingly, the immune cell subtypes were among the top 2 cell types with the lowest *J*_comp_ values. In contrast, in the PD-L1 high tissue, the patterns of the tumor cell type (tS2) and MAST or NK/T cells overlapped in several tissue subregions (Fig. 3b), with *J*_local_ ranging from 0.108 to 0.848 in MAST cells and 0.024 to 0.632 in NK/T cells (Additional file 1: Fig. S7). The tissue subregions with the top *J*_local_ scores were shared between the two immune cell types. In addition, MAST and NK/T cells belonged to the top 2 cell types with the highest *J*_comp_ (Fig. 3b), with MAST cells showing significant spatial overlap with tumor cells (*p* < 0.001; marked with a white asterisk). Notably, in the PD-L1 low tissue, fibroblasts colocalized with the tumor cell type (tS2) at the border of the tumor defined by CCs of tS2; however, in PD-L1 high tissue, the two cell types showed little overlap.

To further dissect the tumor and T cell interactions in PD-L1 high and low tumors, the spatial relationship between tumor cells and T cell subtypes was investigated. Spatial overlap patterns of the tumor cell type (tS2) with T cell subtypes, including naïve CD4 + T cells, CD4 + helper T cells (Th), CD8 + /CD4 + mixed Th cells, exhausted follicular Th (Tfh) cells, regulatory T cells (Treg), naïve CD8 + T cells, cytotoxic CD8 + T cells, exhausted CD8 + T cells, CD8low T cells, and other nonspecified T cells (T lymphocytes_ns), were calculated. Overall, PD-L1 low tumors did not show significant spatial overlap between various T cell subtypes and tS2 (*J*_comp_: 0.000–0.142), except for exhausted T cell subtypes, which were colocalized at the border of tS2 (Fig. S8a, c). Conversely, PD-L1 high tumor, naïve, effector, and exhausted T cell subtypes were highly colocalized with tS2 (*J*_comp_: 0.318–0.530) (Additional file 1: Fig. S8b, d). Interestingly, Tregs showed the lowest overlap with tS2 (*J*_comp_: 0.101), while cytotoxic CD8 + T cells presented the highest overlap with tS2 (*J*_comp_: 0.530). This finding implies that the TME is less suppressive to T cells in the selected PD-L1 high tissue (*spa18ca02*) compared to the PD-L1 low tissue (*spa06ca01*).

**Fig. 4:**
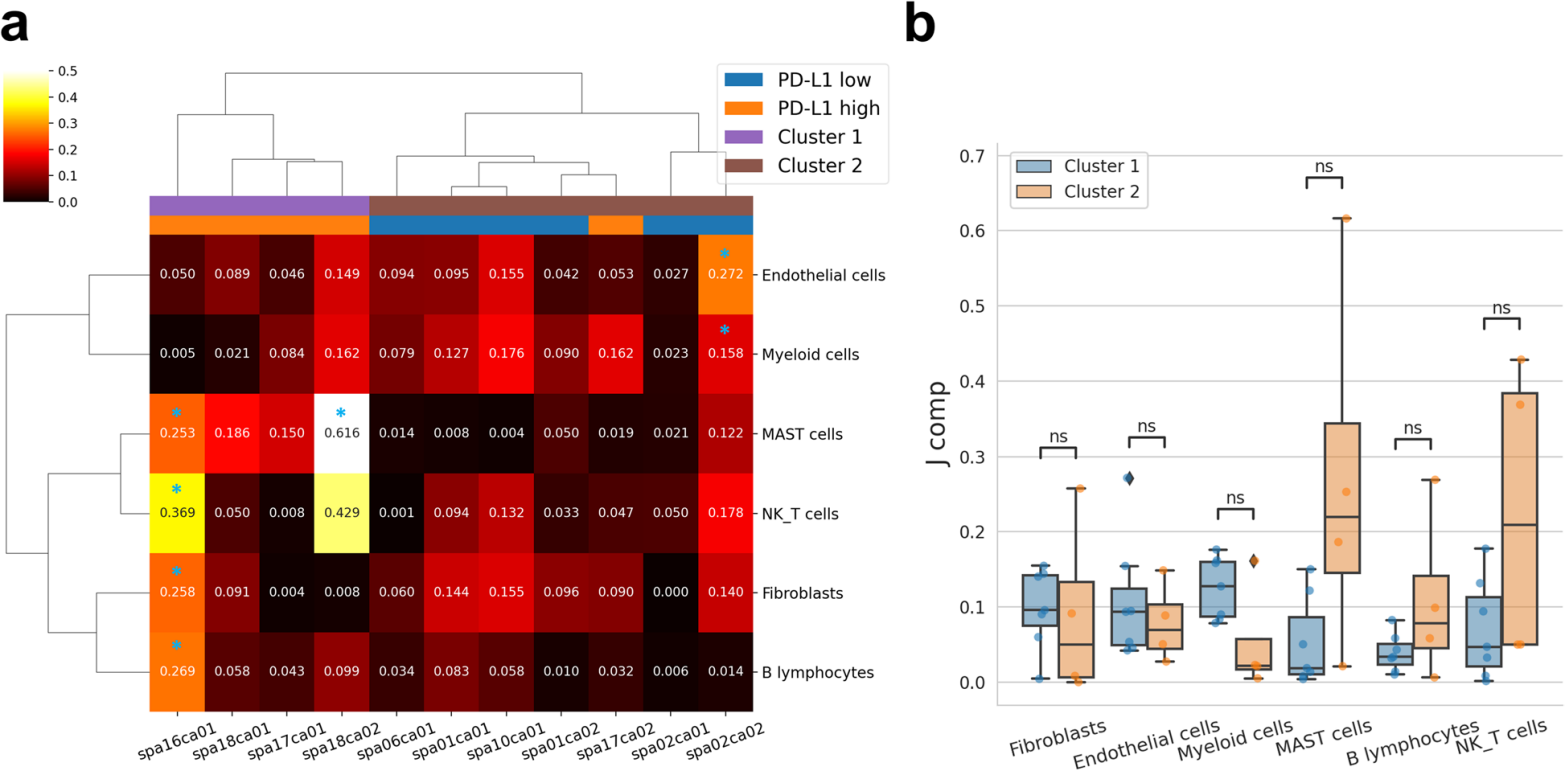
STopover clusters multiple lung cancer tissues based on the cell colocalization pattern in the TME using barcode-based SRT. The barcode-based SRTs of eleven lung cancer tissues were analyzed with STopover. The extent of spatial overlap between the tumor cell type (tS2) and other main cell types was represented by the set-based *J*_comp_ scores. **a** *J*_comp_ values between tS2 and other main cell types in 11 lung cancer tissues were visualized with a heatmap. The Pearson correlation distances were computed across all cell type pairs and all tissue pairs, and hierarchical clustering was performed. The tissues were classified into two clusters: Clusters 1 and 2. In the heatmap, statistically significant colocalization between tS2 and other cell types is visualized as a blue asterisk (*p*<0.05). **b** The *J*_comp_ values were compared between Clusters 1 and 2 in every cell type and visualized with a boxplot. Wilcoxon rank-sum tests were performed, and the Bonferroni method was applied for multiple comparison corrections. In summary, STopover could classify multiple tissues into two distinct TME profiles. ns: not significant

Then, STopover analysis was performed across 11 lung ADC data, and the spatial configuration of the TME in various lung cancer tissues was explored. A total of 6 tissues (*spa01ca01*,* spa01ca02, spa02ca01, spa02ca02, spa06ca01, spa10ca01*) had low PD-L1 expression (0% in all samples), and the other 6 tissues (*spa16ca01, spa17ca01, spa17ca02, spa18ca01, spa18ca02*) had high PD-L1 expression (range: 80–100%) (Additional file 2: Table S1). First, the correlation between pseudobulk expression of ICI response biomarkers (PD-L1 and MHC class I) and spatial overlap of the tumor cells (tS2) and T cells represented by set-based *J*_comp_ as a quantitative value for representing topological T cell infiltration was examined. The expression of *HLA-B* and *HLA-C*, which encode MHC class I, showed a significant positive correlation with *J*_comp_; however, *CD274*, which encodes PD-L1, and *PDCD1*, which encodes PD-1, did not show a significant correlation (Additional file 1: Fig. S9). Second, *J*_comp_ was calculated between the tumor cells (tS2) and other main cell types and was visualized with a heatmap (Fig. 4a). Among the 7-cell types, MAST and NK/T cells showed a similar trend of *J*_comp_ values across the tissue samples and were clustered together. Meanwhile, the 11 tissue samples were clustered into two groups (“Cluster 1” and “Cluster 2”) according to the trend of spatial overlap between tS2 and other cell types. The significance of colocalization was determined by performing a permutation and calculating the *p*-value based on the null distribution. In the case of Cluster 1, two tissues with significant cell types commonly showed significant colocalization between tumor and MAST cells (*spa16ca01*, *p* = 0.011; *spa18ca02*, *p* < 0.001), whereas in Cluster 2, one tissue with significant cell types showed colocalization between tumor-myeloid (*spa02ca02*, *p* = 0.016) and tumor-endothelial (*spa02ca02*, *p* = 0.015) cell pairs (Fig. 4a; marked with blue stars). Furthermore, compared to Cluster 1, Cluster 2 showed higher median *J*_comp_ scores in MAST, B, and NK/T cells and lower scores in fibroblasts, endothelial cells, and myeloid cells (Fig. 4b). Thus, Cluster 2 represents the TME with high infiltration of lymphocytes, especially MAST cells, and low involvement of myeloid cells and fibroblasts. Lastly, to quantify subregional cell infiltration patterns in TME within each CC, the weighted Jaccard index was additionally applied. The weighted Jaccard index exhibited a strong positive correlation with the set-based Jaccard index between tumor cells and other cell types. The differences in the extent of cell infiltration were more pronounced across the 11 lung cancer tissues when using the weighted Jaccard index (Additional file 1: Fig. S10). In summary, STopover can describe and quantify the spatial relationship between cancer-immune and cancer-stromal components in lung cancer, particularly in barcode-based SRT.

### STopover estimates spatial cell–cell interactions in barcode-based SRT of lung cancer

**Fig. 5:**
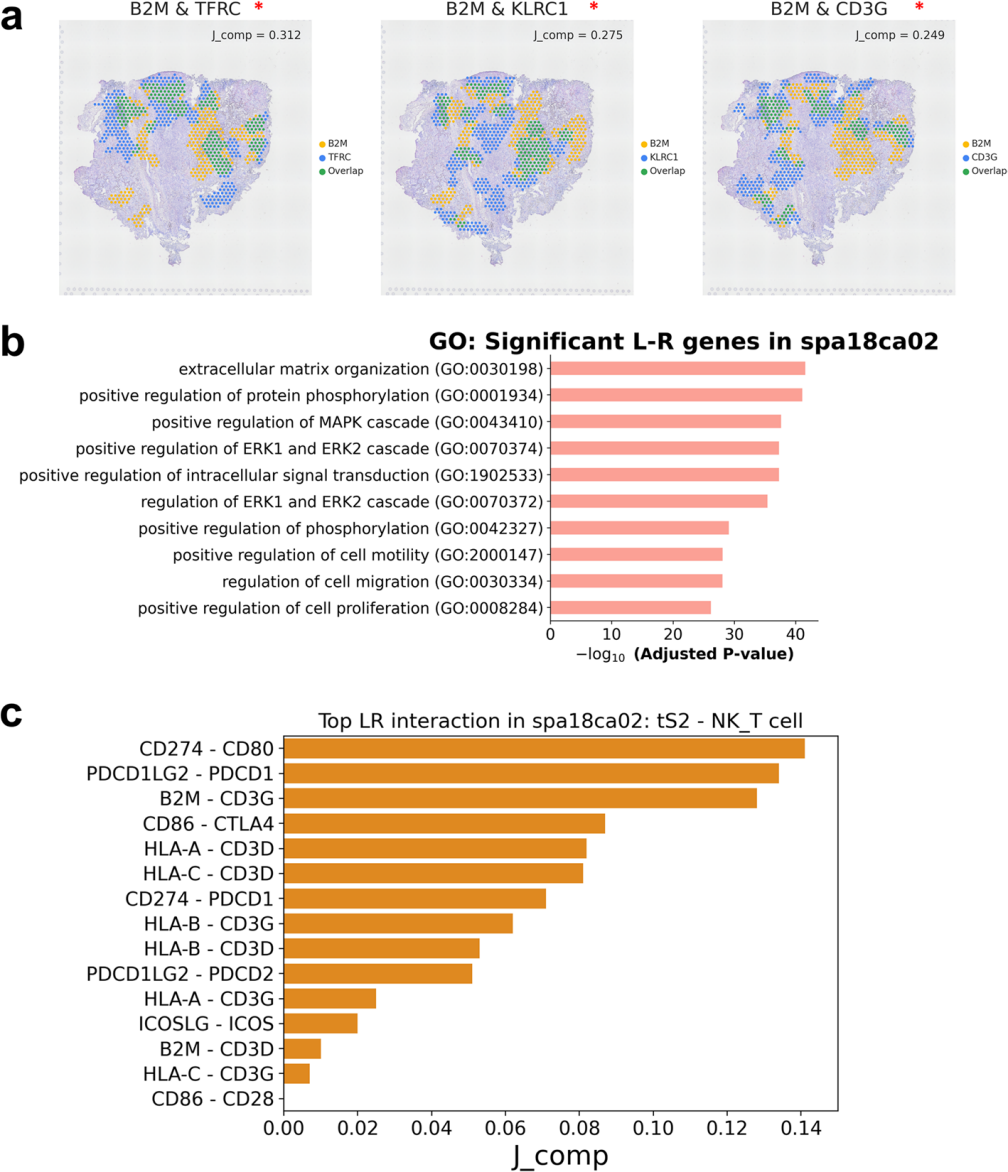
STopover predicts dominant cell–cellinteractionsin lung cancer tissue using barcode-based SRT. Based on the presumption that cell–cell communication mediated by LR interaction occurs in close proximity, spatial overlap patterns between the LR pairs were searched based on the CellTalkDB database [26]. The top LR pairs showing a high overlap score represented by the set-based *J*_comp_ were considered dominant cell–cell interactions in the given tissue. The LR pairs with a *J*_comp_ score over 0.2 were selected. **a** Among the filtered LR pairs, the location of CCs for the top 3 pairs showing the highest average ligand gene expression in the tissue and the highest *J*_comp_ value was mapped to the tissue. CC locations for features *x* and *y* are colored yellow and blue, respectively, and intersection locations are shown in green. In the spatial plots, statistically significant colocalization between tS2 and other cell types is visualized as a red asterisk (*p*<0.05). **b** Gene Ontology (GO) analysis was performed for all of the filtered LR pairs, and the enriched biological process terms are listed in ascending order of adjusted *p* values. To further investigate the cell–cell communication that occurs specifically between tS2 and T cells, 15 LR pairs closely related to T cell action were chosen, and their spatial colocalization patterns were extracted with STopover. Then, extracted CCs for LR pairs were intersected with the colocalized domain between tS2 and NK/T cells, and the modified CCs were presumed to represent key locations for interaction between tS2 and NK/T cells. **c** The set-based *J*_comp_ scores were calculated between the modified CCs, and the 15 LR pairs were listed in descending order of *J*_comp_. As a result, STopover could be adopted as a tool to screen dominant cell–cell interactions and their functional implications in cancer tissue

STopover can be applied to capture the overlapping location of ligand-receptor (LR) expression. Based on the assumption that LR interaction mostly occurs between cells in proximity, the colocalized tissue domain extracted between LR pairs can be considered a key location for cell–cell interaction. The LR gene pairs provided by CellTalkDB [26] and their expression profiles were utilized to estimate the key location and extent of the cell–cell interaction. Last, the strength of the interaction was ranked by *J*_comp_ values between LR pairs. A representative tissue slide with high PD-L1 expression (*spa18ca02*) was selected, and the top LR pairs with *J*_comp_ over 0.200 were extracted (Additional file 2: Table S2). The top 3 significant LR pairs with the highest ligand gene expression and *J*_comp_ were *B2M*-*TFRC* (*p* < 0.001), *B2M-KLRC1* (*p* < 0.001), and *B2M*-*CD3G* (*p* = 0.005) (Fig. 5a and Additional file 1: Fig. S11). The main location of the LR pairs corresponded more to myeloid cells than to the tumor cell type (tS2) distribution (Additional file 1: Fig. S12). Next, an enrichment analysis was performed for the extracted LR pairs with significant *p*-values (*p* < 0.05), and the top 10 Gene Ontology (GO) terms associated with LR pairs were extracellular matrix, ECM organization, and MAPK/ERK pathway (Fig. 5b), which are closely related to cancer cell proliferation and metastasis [39]. To comprehend the common and distinct LR interactions in 11 lung ADC tissues, the common LR interactions within Clusters 1 and 2 were calculated and compared. In the case of Cluster 1, the top 10 GO terms related to LR interaction were ECM organization and kinase signaling, while in Cluster 2, ECM organization was prominent along with cell–matrix adhesion and cell migration (Additional file 1: Fig. S13). The common LR interaction between Clusters 1 and 2 was *SPINT4*-*ST14*. Since cell–cell interaction analysis relies on prior information from LR databases, we applied a different database, OmniPath [27], to investigate how the choice of database affects the ranking of the top LR pairs. Similar to the results derived from CellTalkDB, LR pairs with *J*_comp_ over 0.200 were associated with GO terms such as protein phosphorylation and cytokine-mediated signaling pathway. However, it also revealed slightly different GO terms, including regulation of the apoptotic process and protein ubiquitination (Fig. 5b and Additional file 1: Fig. S14a). While a larger LR database may yield more reliable results by considering all possible LR pairs, the top 10 LR pairs with the highest ligand expression were similar between the two databases (Additional file 1: Fig. S14b and Additional file 2: Table S2).

To focus on the tumor-immune interaction in the selected high PD-L1 tissue, LR pairs related to T cell activation or suppression were chosen [40]. The CCs for the selected LR pairs were computed, and *J*_comp_ was calculated for the spots corresponding to the tissue region where tS2 and NK/T cells were colocalized. Among the LR pairs related to T cell action, *CD274*-*CD80* (*J*_comp_: 0.141, *p* = 0.383), *PDCD1LG2-PDCD1* (*J*_comp_: 0.134, *p* = 0.231), *B2M*-*CD3G* (*J*_comp_: 0.128, *p* = 0.121), and *CD86*-*CTLA4* (*J*_comp_: 0.087, *p* = 0.495) were the top gene pairs estimated to have high spatial overlap (Fig. 5c and Additional file 1: Fig. S15), although none of the pairs showed statistically significant colocalization. This finding suggests that T cell activation and inhibition signals both exert action at the T cell infiltrating tissue domain of the selected PD-L1 high tissue. In short, STopover can provide unbiased information on dominant spatial cell–cell interactions in lung cancer tissue and list the key components of cancer and T cell interactions.

### STopover extracts the spatial configuration of the lung cancer TME in image-based SRT

To prove the scalability of STopover in image-based SRT, the method was applied to the CosMx SMI platform in which the predefined RNA transcripts are detected by fluorescence signal, and cell-level RNA expression is calculated by cell segmentation. Lung cancer tissue was selected to investigate the TME. For convenience, the FOV of CosMx data was divided into 100 by 100 grids, and RNA transcripts were assigned to each grid based on the 2D coordinates (Additional file 1: Fig. S1a). The fraction of a certain cell in a grid was determined by the ratio of RNA counts in each grid belonging to the cell to the total count across the grids that the cell encompasses (Additional file 1: Fig. S1b). Then, by utilizing a cell-level RNA count matrix and a reference lung cancer single-cell dataset [12], each cell could be classified into cell types defined from the single-cell data (Additional file 1: Fig. S1c). The abundance of a certain cell type was obtained by collecting the cells belonging to the cell type on each grid and calculating the sum of the cell fractions (Additional file 1: Fig. S1d). Additionally, cell-type-specific expression was estimated by extracting the RNA transcript in each grid corresponding to the cell type. As a result, the image-based SRT was converted to grid-based expression data, and similar strategies were applied as with barcoding-based SRT to calculate overlapping spatial domains of feature pairs.

**Fig. 6:**
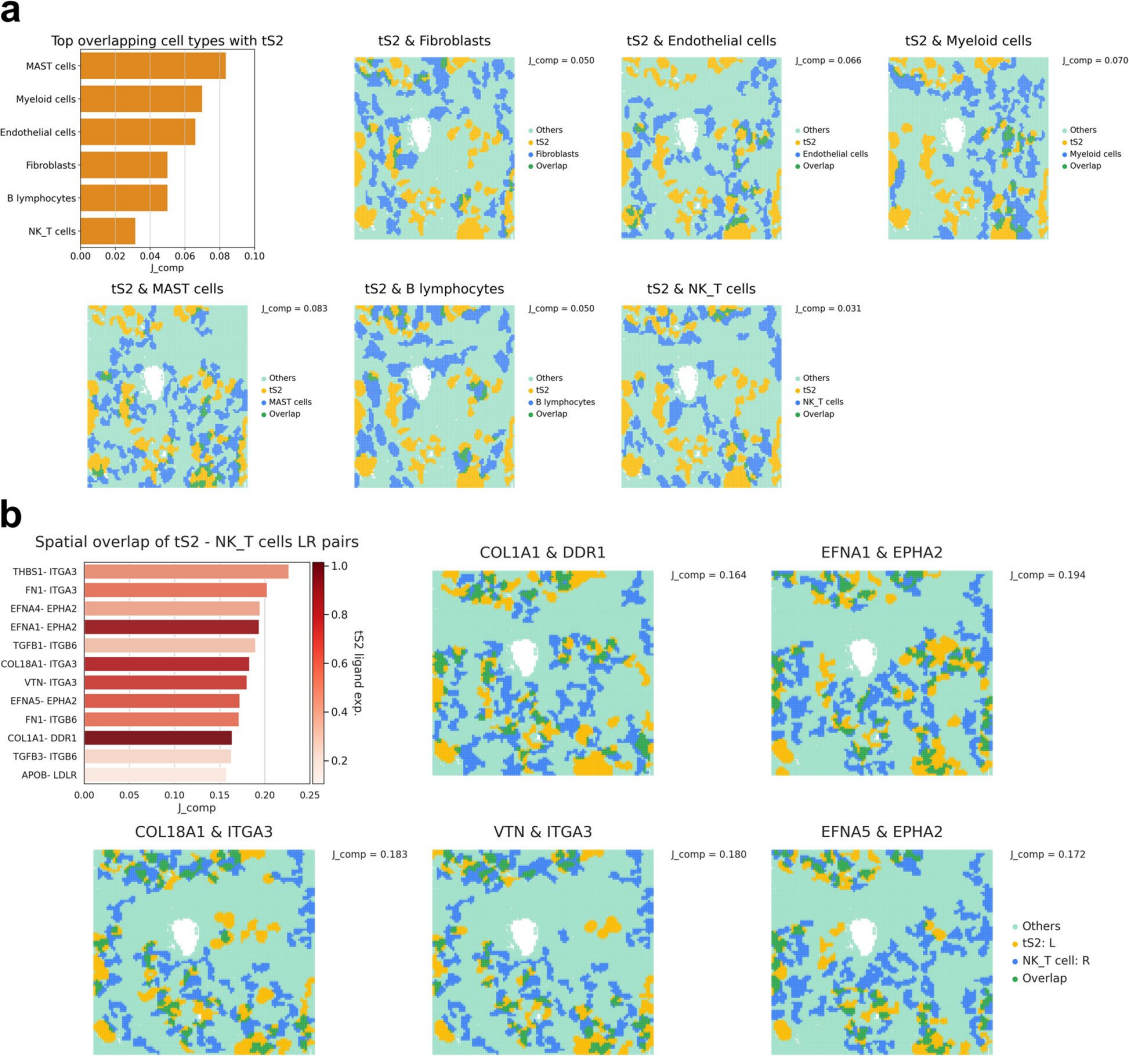
STopover captures cell colocalization patterns and estimates tumor and NK/T cell crosstalk in lung cancer tissue using image-based SRT. The STopover was applied to image-based SRT of lung cancer tissue and deciphered spatial patterns of the cell types and their overlap. **a** The set-based *J*_comp_ scores were calculated between the tumor cell type (tS2) and other main cell types and were visualized with a bar plot at the top left corner. The locations of CCs for tS2 (yellow) and other cell types (blue) were mapped to the tissue, and the overlapping domain is highlighted in green. The cell type-specific RNA counts were extracted from image-based SRT, and LR analyses were implemented using tS2-specific ligand expression and NK/T cell-specific receptor expression. In the bar plot, statistically significant colocalization between tS2 and other cell types is visualized as an asterisk (*p*<0.05). **b** The set-based *J*_comp_ scores were computed for the LR pairs, and the results are presented in a bar plot in descending order of *J*_comp_. The average expression of ligand in tS2 was color-coded on the bar plot. Additionally, the location of CCs for tS2-specific ligand and NK/T cell-specific receptor and their overlapping subregions were visualized on the tissue. In short, STopover can be flexibly applied to image-based SRT to calculate cell–cell colocalization patterns and predict key intercellular communications

First, the spatial abundance of cell types (Additional file 1: Fig. S16) and spatial colocalization patterns between the tumor cell type (tS2) and major lung cancer cell types (Fig. 6a) were computed and visualized. Overall, the extracted CCs matched the spatial distribution pattern of cell types. When calculating the global overlap score, *J*_comp_, the scores were consistently low for all cell types, with fibroblasts exhibiting slightly higher *J*_comp_ than NK/T cells (Fig. 6a). Furthermore, none of the cell types showed statistically significant colocalization with tumor cells. Additional STopover analysis was performed between tS2 and T cell subtypes (Additional file 1: Fig. S17). *J*_comp_ scores were low across all cell subtypes, and CD8 + T cells, including naïve CD8 + T cells and cytotoxic CD8 + T cells, showed the lowest overlap with tS2 (*J*_comp_: 0.007 and 0.022).

### STopover predicts NK/T cell-specific cell–cell interactions in image-based SRT of lung cancer

STopover was applied to the lung cancer CosMx SMI dataset, and tumor cells (tS2) and NK/T cell-specific cell–cell interactions in the TME were explored. The list of LR pairs was refined by searching the intersection between genes present in the CosMx dataset and those from CellTalkDB. Then, ligand gene expression in tS2 cells and receptor gene expression in T cells were utilized to predict tumor-immune-specific interactions. The analysis revealed 12 key interactions between tumor and T cells with a *J*_comp_ score over 0.150 (Fig. 6b and Additional file 2: Table S3). Among the pairs, *COL1A1*-*DDR1* had the highest average ligand expression in the tissue, followed by *EFNA1*-*EPHA2*, *COL18A1*-*ITGA3*, *VTN*-*ITGA3*, and *EFNA5-EPHA2*. However, none of the interactions were statistically significant, indicating low interaction between tumor and NK or T cells. The spatial interaction pattern between tS2 and NK/T cells was compared across the 12 LR pairs. Overall, the estimated location of the interaction between tS2 and NK/T cells was highly overlapped across the top 5 selected LR pairs with high average ligand expression (Fig. 6b and Additional file 1: Fig. S18). In summary, STopover can be extensively used in image-based SRT to decipher the spatial patterns of the lung cancer microenvironment and rank the strong interaction specific to the tumor and NK/T cells.

### STopover unveils spatial characteristics of breast cancer TME using barcode-based SRT

**Fig. 7:**
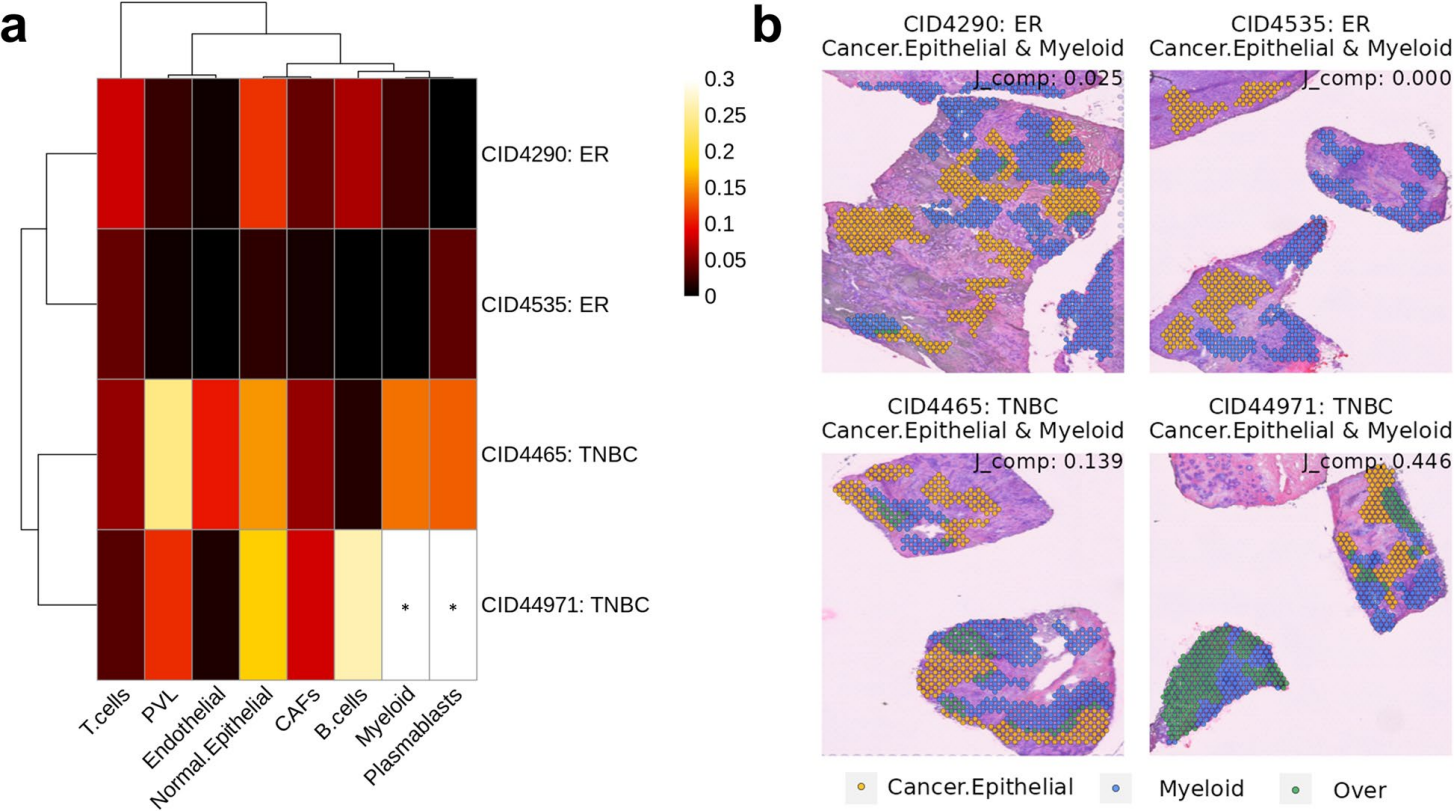
STopover characterizes spatial heterogeneity of breast cancer tissues using barcode-based SRT. The barcode-based SRTs of breast cancer tissues were analyzed using STopover. First, cell–cell colocalization patterns were extracted and compared across the tissues. **a **The set-based *J*_comp_ values between the cancer epithelial cells and other main cell types in four breast cancer tissues were visualized with a heatmap. The Pearson correlation distances were calculated between all cell type pairs and all tissue pairs, and hierarchical clustering was performed. In the heatmap, statistically significant colocalization between cancer epithelial cells and other cell types is visualized as a black asterisk (*p*<0.05). **b** The CCs of the epithelial cancer cells (yellow) and other cell types (blue), and the intersecting subregions (green) between the two aggregated CCs were visualized on the tissue

Although breast cancer is generally considered a less immunogenic tumor, recent studies have highlighted that certain cancer tissues show high levels of immune infiltration and respond well to ICIs [41]. To comprehend the heterogeneity of immune profiles in breast cancer, STopover was applied to barcode-based SRT and spatial interaction was compared across several cancer tissues. The immune phenotype was explored in two different cancer subtypes: estrogen receptor-positive (ER +) and triple-negative breast cancer (TNBC). The spatial composition of cancer, immune, and stromal cells was predicted based on reference single-cell dataset [16], and the colocalization pattern between cancer epithelial cells and other cell types was analyzed. Two ER + samples displayed low immune infiltration and exhibited distinct spatial patterns of TME compared to the TNBC samples (Fig. 7a). Among all cell types, the average fold change of *J*_comp_ was the highest for myeloid cells between the ER + and TNBC subtypes (fold change ratio of *J*_comp_ in TNBC to ER + : 23.511). Besides, myeloid cells showed statistically significant colocalization with cancer epithelial cells in one of the TNBC tissue (*p* < 0.001). Myeloid cells and cancer epithelial cells were highly exclusive in ER + tissues, whereas myeloid cells infiltrated inside cancer epithelial cells in TNBC (Fig. 7b).

Then, the dominant spatial LR interaction and their key niche were predicted in the breast cancer tissues. Differentially upregulated or downregulated LR pairs in ER + compared to TNBC were found (Additional file 2: Table S4), and the enrichment analysis was performed. The top 5 GO terms were identified and the corresponding LR pairs were presented in heatmaps (Additional file 1: Fig. S19a, b). In ER + tissues, the top upregulated LR interaction (*LAMB2*-*RPSA*) was associated with the organization of the extracellular matrix. In contrast, in TNBC tissues, the top LR interaction (*IL18*-*IL1RL2*) was related to the positive regulation of kinase activity and positive regulation of cell adhesion. The main site of the *LAMB2*-*RPSA* interaction was more restricted to the cancer-associated fibroblast (CAF) niche than to the cancer cell niche (Additional file 1: Fig. S20a, c), represented by the fold change (FC) of the average *J*_comp_ of CAF to cancer cell niche in ER + tissues of 2.763. The *IL18*-*IL1RL2* showed a similar level of interaction in both the CAF and cancer cell niche (Additional file 1: Fig. S20b, d), with the FC of CAF to cancer cell niche in TNBC tissues of 1.133. In short, *IL18*-*IL1RL2*, the most upregulated LR interactions in TNBC, showed an expanded interaction over the CAF region, compared to *LAMB2*-*RPSA*. To further explore the top LR interactions upregulated in ER + and TNBC tissues that were more specific to the cancer cell niche than the CAF niche, we calculated the FC of *J*_comp_ between the two niches, in ER + and TNBC tissues, respectively, and ranked the FC values. For the LR pairs upregulated in ER + tissues, the pairs with the higher interaction in the cancer cell niche (FC > 1) were associated with the bone morphogenic protein (BMP) signaling pathways (top 1: *GDF9*-*BMPR1B*). Conversely, for the pairs upregulated in TNBC tissues, the pairs with the highest FC were associated with ECM organization and kinase signaling mediated cell proliferation (top 1: *DLL3*-*NOTCH2*). This comparative analysis is instrumental in identifying functional similarities and differences within the cancer niche according to the cancer subtypes, thereby enhancing our understanding of cell–cell interactions across different breast cancer subtypes. In brief, STopover could be utilized to characterize the TME landscape in diverse tumor tissues with different molecular profiles.

### Comparison with other methods for spatial characterization of the TME

A few computational tools have been developed to enable unbiased profiling of the spatial heterogeneity of the tissue based on SRT. Among the available methods, ATHENA, Squidpy, spatialGE, SpaCET, and stLearn were compared with STopover in terms of their functionality or performance [33, 35–38].

As the cell infiltration score estimated by ATHENA uses the neighbors of each cell instead of overlapping cell density scores in barcode-based ST, it can only be used in image-based SRT data that directly define cell types [42]. ATHENA could rank the cell types that are colocalized with tumor cells, and visualize the key location of the cell–cell overlap (Additional file 1: Fig. S21a, b). The global and local infiltration patterns of cell types into the lung cancer were estimated using ATHENA. MAST cells were identified as the top infiltrating cell type by both ATHENA and STopover (Fig. 6a and Additional file 1: Fig. S21a). However, ATHENA faced difficulties in capturing subregions characterized by low tumor density but high immune cell concentration (Fig. 6a and Additional file 1: Fig. S16, S21b). Also, it was unable to detect molecular interactions that specifically occurred between tumor and immune cells.

In the case of Squidpy, cell colocalization patterns and LR interactions were analyzed in image-based SRT by shuffling the cell labels. In the lung cancer dataset, Squidpy revealed that NK_T cells (− 134.32), B lymphocytes (− 119.07), myeloid cells (− 102.47), fibroblasts (− 61.57), endothelial cells (− 51.47), and MAST cells (−30.65) exhibited increasing colocalization with tumor cells, all with negative *Z*-scores (Fig. 6a and Additional file 1: Fig.S21c). Furthermore, none of the LR interactions between tumor cells and NK_T cells were significant (Fig. 6b). Overall, STopover produced similar results to Squidpy, further highlighting the low immune cell infiltration in the lung cancer tissue. However, Squidpy was not applicable to barcode-based SRT, where the spot is composed of a mixture of cells.

Meanwhile, with the use of spatialGE, the tumor regions in lung cancer could be distinguished, and the immune phenotypes of lung cancer tissues with high or low PD-L1 expression could be visualized using barcode-based SRT (Additional file 1: Fig. S21d, e). However, spatialGE is limited in its ability to quantify the extent of subregional immune infiltration, investigate molecular interaction, and be applied to the image-based SRT platform.

In the case of SpaCET, it reveals the locations of the colocalization between cell type pairs and highlight dominant LR interactions between theses cell types. However, SpaCET evaluates the intensity of cell–cell colocalization utilizing Spearman’s correlation, without taking into account the spatial distances between spots in barcode-based ST. Consequently, it might not precisely identify the spatially clustered distribution patterns of cells, potentially resulting in noisier estimations of cell–cell colocalization regions. For instance, in lung cancer datasets, both STopover and SpaCET captured the overall colocalization patterns between tumor cell type (tS2) and NK/T cells (Fig. 3 and Additional file 1: Fig. S22a, b). Nevertheless, SpaCET failed to identify some of the subregions where NK/T cells were densely concentrated in a small area.

Finally, with stLearn, it is possible to extract spatial colocalization and LR interaction patterns between specific cell type pairs in barcode-based ST. The top LR interaction was *B2M-HLA-F*, *HLA-B-CANX*, and *B2M-LILRB1*, and associated biological processes were similar with those derived from STopover (Fig. 5b and Additional file 1: Fig. S22c, d). However, the ligand-receptor coexpression locations predicted by stLearn tend to exhibit more noise compared to the predictions made by STopover (Additional file 1: Fig. S22e, f). STopover not only captures global colocalization patterns but also identifies and rank subregions, pinpointing where cell–cell or ligand–receptor interactions are most likely to take place.

## Discussion

Decoding the spatial relationship between tumor, immune, and stromal cells in the TME is crucial to understanding the immune cell action on cancer cells and predicting responses to immunotherapy. Recent advances in SRT techniques have allowed for the screening of spatial patterns of multiple cell types and their gene expression in heterogeneous cancer tissue. In the case of barcoding-based spatial transcriptomics, the tissue is divided into small unit regions, and genome-wide RNA expression is investigated. This technique has been widely utilized because it can screen RNA expression across a wide range of tissues without predefined RNAs of interest [43–48]. Meanwhile, image-based spatial transcriptomics allows for spatial profiling of RNA expression in units of cells by identifying the RNA sequence, specifying the location using a fluorescence signal, and drawing the cell boundary [49–54]. If the two complementary spatial transcriptomic technologies are adopted, the spatial composition of the various cells constituting the tissue can be explored, and the complex interaction between the cells can be estimated.

In this regard, we developed STopover, which utilizes barcodes and image-based SRTs to summarize the topological colocalization pattern between cell types and LR pairs from CCs acquired by Morse filtration. By quantifying and visualizing the overlap between CC pairs, we compared the tumor infiltration of fibroblasts and immune cells in multiple cancer tissues. We utilized two different methods to measure the extent of cell infiltration: set-based Jaccard index, which assesses binary cell overlap, and the weighted Jaccard index, which considers subregional distribution patterns. The key locations for cell infiltration were highlighted by ranking the local overlap of CCs between the feature pair. In addition, given that cell–cell interactions mediated by LR interactions occur in close proximity, major intercellular communication in cancer tissue could be estimated by finding the colocalization pattern of LR pairs. As an example, by utilizing cell type-specific RNA counts obtained from image-based SRT, STopover could list key spatial communication between tumor and T cells in lung cancer tissue.

One of the key features of STopover is capturing locally active regions of cell–cell colocalization in the TME. Based on the functionality, STopover could dissect immune-excluded and immune-inflamed environments in one of the PD-L1 low and high lung cancer tissues, respectively (Fig. 3). The TME of the two tissues was characterized by contrastive spatial overlap patterns of tumor-stromal and tumor-immune cells. Also, in breast cancer tissues, STopover was able to capture the distinct immune phenotypes present in ER + and TNBC tumors, with ER + tissues exhibiting immune-excluded patterns and TNBC showing immune-inflamed patterns (Fig. 7). This is consistent with previous reports indicating that the TNBC subtype is more likely to be immunogenic and contains a higher amount of T cells and myeloid cell population than other subtypes [41, 55]. The STopover was applicable not only in barcode-based SRT but also in image-based SRT platforms to understand the TME (Fig. 6). Moreover, the cross-cell type colocalization patterns in the tumor were represented by the global overlap score, *J*_comp_, and the lung cancer tissues could be classified into two clusters with distinct TME profiles (Fig. 4). The two clusters did not completely match the group divided by PD-L1 expression, implying that STopover can describe the configuration of the TME independently of PD-L1 levels in cancer. In addition, STopover offers biologically relevant information regarding immunotherapy response. As an example, the *J*_comp_ score across the 11 lung cancer tissues was positively correlated with the expression of genes coding MHC-class I protein (*B2M*, *HLA-A*, *HLA-B*, and *HLA-C*) (Additional file 1: Fig. S9), which are among the biomarkers for immunotherapy response [5].

Another important functionality of STopover is that it can predict spatial tumor-immune interactions by ranking coexpression patterns of LR pairs in the whole tissue. One similar approach inferred cell–cell communication from the curated LR database by searching LR coexpression in the neighboring regions and measuring the number of distinct cell types [38]. Other approaches split the variability of gene expression into multiple factors [56, 57] or use graph neural networks [58, 59] to model spatial cell–cell interactions. Compared to the suggested methods, STopover is a platform-agonistic method that can be utilized in both image- and barcode-based SRT and segment the key regions of the top-ranked cell–cell interaction. In this regard, STopover was applied to both lung and breast cancer tissues and elucidated spatial cell–cell communication underlying the tumor heterogeneity at the molecular level. In one of the barcode-based SRT datasets for lung cancer, the top LR pairs with high spatial overlap were enriched with GO terms related to the development of the TME [39] (Fig. 5b). In particular, the top 3 pairs were explained by cell–cell interactions via MHC class I molecules (Fig. 5a), and their main location of interaction was highly overlapped with the domain of myeloid cells but exclusive to tS2 (Additional file 1: Fig. S12). This finding implies that the MHC class I-mediated process, which is one of the most activated cell–cell communications, occurs primarily at the myeloid cell niche. In the case of the image-based SRT dataset of lung cancer, tumor- and T cell-specific expression profiles could be extracted, and the tumor and T cell interaction could be more specifically investigated (Fig. 6b). A total of 12 LR pairs were selected as meaningful interactions, and several interactions were reported to be regulators of TME. Integrins and discoidin domain receptor 1 (DDR1) expressed on T cells interact with collagen and induce T cell migration or an inflammatory response [60]. Besides, EphrinA (EFNA), which binds to EphA receptors, is associated with immune cell infiltration in the tumor [61]. Meanwhile, the main location for tumor and T cell communication was redundant across the top LR interactions (Fig. 6b), suggesting the tumor-T cell interaction in the constrained subregions. Lastly, in breast cancer datasets, molecular interaction was explored, and ER + and TNBC subtypes had a different spatial predominance of interaction. The top interaction present in ER + tumors is closely related to the ECM and it is localized in fibroblast regions where the ECM is actively being formed (Additional file 1: Fig. S20c). On the other hand, the main interaction in TNBC is associated with kinase activity, cell adhesion, and phosphorylation and is not restricted to fibroblast regions, but extended to cancer regions where cell proliferation is ongoing (Additional file 1: Fig. S20d). This is consistent with previous studies indicating that cancer cell growth is more enhanced in TNBC than in ER + cancers [41]. The results imply that the molecular interaction captured by STopover well reflects the spatial predominance of these interactions.

The primary limitation of the study lies in the absence of longitudinal clinical data, making it impossible to directly analyze the association between the spatial interaction of cells characterized by STopover and the clinical progression of cancer. Nevertheless, a correlation was found between cell infiltration patterns and interactions, and the molecular profiles of cancer tissue. The discoveries in lung and breast cancer tissues link established molecular-phenotypic associations with spatial interaction profiles, indirectly validating the results. Looking ahead, STopover could be broadly applied to search for biomarkers that faithfully capture the intricate spatial interaction patterns across heterogeneous cancer tissues.

There are several points to consider when applying STopover to dissect the TME in terms of cell–cell interactions. Care must be taken when interpreting the top cell–cell interaction extracted by STopover in barcode-based SRT. Because the LR interaction is searched for all the cell types present in the unit domain of the tissue, the highlighted location does not indicate the specific interaction between the two cell types. An alternative approach to estimate cell type-specific interactions might be constraining the CCs of LR pairs to the spatial overlapping niche between the two cell types of interest. When this alternative approach was applied to image-based SRT, the regions of cell-type-specific interaction were limited to the location of colocalized T cells within the tumor (Additional file 1: Fig. S23a), which is more restricted to small subregions than the actual cell type-specific interaction patterns (Additional file 1: Fig. S23b). In addition, there is a possibility that highly expressed LR pairs are more likely to be selected during LR interaction analysis. To address this concern, regions with high or low feature values are given equal weights when calculating the Jaccard score between two features. The top LR interactions were consistent with those obtained by other methods of LR analysis (Additional file 1: Fig. S22c, d), supporting the biological relevance of results. Nevertheless, caution should be exercised as the bias towards highly expressed genes may not be completely eliminated and further experimental validation is required. It is also important to note that STopover focuses on the LR interaction and does not consider the downstream signaling pathways, similar to many LR analysis methods using SRT. The methods generally assume that highly colocalized expression of two features indicates a high level of interaction via those feature pairs. However, there may be false positives where two cells have high expression of the two features due to secondary causes or coincidence. Extending this analysis to downstream pathways involves multiple assumptions, which increases the susceptibility to false positives; therefore, we focused on LR interaction only.

Meanwhile, when dealing with barcode and image-based SRTs, STopover relies on cell type proportions or cell type annotations predicted by external methods. The choice of the methods can influence the cell–cell colocalization results in both SRT platforms. In the case of barcode-based SRT for lung cancer, the spatial patterns of NK/T cells varied when utilizing different cell type deconvolution methods, consequently modifying the cell–cell colocalization ranking estimated by STopover (CellDART and Cell2location) (Additional file 1: Fig. S24a-d) [20, 21]. Nonetheless, except for NK/T cells, overall immune cells estimated by Cell2location showed higher overlap with tumor cells (tS2) as shown in STopover using CellDART. However, in image-based SRT for lung cancer, the cell infiltration pattern and ranking were not significantly different depending on cell annotation methods (Ingest and TACCO) (Additional file 1: Fig. S24e, f) [19, 22]. It is advisable to apply STopover after comparing the results of several tools and verifying their accuracy, although we believe that the core algorithm of STopover is computing the co-localization patterns of features rather than estimating the feature distribution. Notably, it is compatible with a variety of cell type deconvolution methods, allowing for the analysis of cell type colocalization patterns following deconvolution.

Regarding the choice of the colocalization index, one might ask which of the Jaccard indices, set-based or weighted index, is most optimal for quantifying cell infiltration. Both methods yield similar ranking of cell infiltration into the tumor with a strong positive correlation. Notably, the weighted method allows for more sensitive detection of differences in cell infiltration patterns among cancer tissues (Additional file 1: Fig. S10), although it may be influenced by noise, as demonstrated in the simulation examples (Additional file 1: Fig. S5). Furthermore, *J*_comp_ is one of parameters generated by STopover, and it is possible to derive different values in different cases. For example, when evaluating the degree of infiltration by immune cells (CC1) into the tumor (CC2), it is also possible to have the proportion of overlapped regions among the regions in CC2 as a measure, which is a factor that can be determined thematically according to its biological and clinical significance.

In the barcode-based SRT platform, lateral diffusion, which refers to the diffusion of RNA molecules to adjacent spots, is another issue that degrades the data quality and affects STopover analysis. While STopover employs dendrogram smoothing to mitigate noise arising from the experimental process, it is crucial to rigorously evaluate the influence of RNA diffusion on the analysis. To this end, we utilized SpotClean, a tool devised to counteract the effects of RNA diffusion, and compared LR interaction analysis results before and after implementing the correction. This strategy allowed us to critically assess and illustrate the robustness of STopover in the context of potential RNA diffusion artifacts in spatial transcriptomic data (Additional file 1: Fig. S25) [62]. The overall spatial patterns generated by STopover remained consistent, and there was a positive correlation between *J*_comp_ before and after the correction (*R* = 0.600), suggesting that RNA diffusion has no significant impact on the analysis (Additional file 1: Fig. S25d).

Finally, STopover is primarily a method that focuses on the interaction and colocalization of cells in infiltrated, closely packed structures. However, it is limited in its ability to capture all cell–cell interactions. For example, it may not reflect certain interactions that occur at the borders of different cells, especially at the borders of cancer cells and immune cells, or paracrine effects that may occur over long distances. However, since the diameter of the spot, the basic unit of the Visium dataset, is 55 µm and the size of the generated grid in the CosMx dataset is 49.214 by 39.376 µm, long-range interactions in the CellTalkDB database, such as cytokine and receptor signaling (characteristic length scale: ~ 100 µm) [63], were presumed not to exceed the range of a few spots or grids. Nonetheless, STopover needs to be considered in conjunction with other existing LR interaction methods utilized in single-cell RNA-seq to gain a thorough understanding.

## Conclusions

STopover is a robust tool that utilizes SRT and topological analysis to analyze the spatial infiltration patterns of the TME and highlights the key niche of tumor–immune and tumor–stromal interactions. The proposed tool is expected to be applicable to elucidate immune evasion mechanisms and guide patient-specific strategies to enhance the efficacy of immunotherapy in cancer.

## Supplementary Information

Additional file 1: Supplementary Figures. Fig. S1. Schematic image for converting image-based SRT to grid-based data. Fig. S2. Extracting CCs by Morse filtration and dendrogram smoothing in Visium dataset. Fig. S3. Parameter optimization for STopover. Fig. S4. Aggregated CCs obtained across various parameters in simulated dataset. Fig. S5. Calculation of weighted Jaccard index from the extracted CC pairs in the simulation dataset. Fig. S6. Spatial distribution of tS2 and other main cell types composing the lung cancer tissue in barcode-based SRT. Fig. S7. The location of the top CC pairs between tS2 and NK/T cells in a PD-L1 high lung cancer tissue. Fig. S8. Colocalization patterns between tS2 and multiple T cell subtypes in barcode-based SRT of lung cancer. Fig. S9. Scatter plots between pseudobulk RNA expression and Jcomp value of tS2 and NK/T cells. Fig. S10. Comparison of set-based and weighted Jaccard index across 11 barcode-based SRT of lung cancer. Fig. S11. Spatial expression of the top 3 ligand-receptor pairs in PD-L1 high lung cancer tissue. Fig. S12. The spatial overlap patterns of the top ligand-receptorpairs constrained to the tS2 or myeloid cell domain in PD-L1 high lung cancer tissue. Fig. S13. The common top ligand-receptorinteractions in Clusters 1 and 2 defined by infiltration patterns of immune and stromal cells to tumor cells. Fig. S14. The top ligand-receptorinteractions estimated by STopover using the Omnipath database. Fig. S15. The spatial overlap patterns of the T cell action-related LR pairs constrained to the tS2 and NK/T cell colocalized domain in PD-L1 high lung cancer tissue. Fig. S16. Spatial distribution of tS2 and other main cell types composing the lung cancer tissue in image-based SRT. Fig. S17. The colocalization patterns between tS2 and multiple T cell subtypes in image-based SRT of lung cancer. Fig. S18. Spatial distribution of the top ligand-receptor expression specific to tS2 and NK/T cells. Fig. S19. The top differentially upregulated LR pairs and related Gene Ontologyterms in ER+ or TNBC tissues. Fig. S20. The spatial overlap patterns of the top differential upregulated LR pairs in ER+ or TNBC breast tissues. Fig. S21. Investigation of spatial heterogeneity of the lung cancer using ATHENA and spatialGE. Fig. S22. Investigation of spatial heterogeneity in lung cancer using SpaCET and stLearn. Fig. S23. Evaluation of a method for estimating cell type-specific LR interaction in barcode-based SRT. Fig. S24. Colocalization patterns predicted by STopover using different cell type deconvolution and annotation methods. Fig. S25. STopover analysis before and after correction for RNA diffusion

Additional file 2: Supplementary Tables. Table S1. Clinical information of the lung cancer patients and pathological profiles of the obtained tissues. Table S2. Estimated ligand-receptorinteraction in the PD-L1 high cancer tissuefrom the Visium dataset based on CellTalkDB or Omnipath databases. Table S3. Estimated ligand-receptorinteraction between tS2 and T lymphocytes from CosMx SMI dataset. Table S4. Differentially upregulated ligand-receptorinteraction in TNBC and ER+ breast cancer tissues from the Visium dataset

## Data Availability

Barcode- and image-based spatial transcriptomic datasets were utilized for the analyses. First, Visium spatial transcriptomic datasets for 11 lung cancer tissues were uploaded on https://zenodo.org/record/7306132 [11]. Second, CosMx SMI spatial transcriptomic dataset for lung cancer tissue named Lung 5–1 was downloaded from the NanoString CosMx SMI FFPE data repository (https://nanostring.com/products/cosmx-spatial-molecular-imager/ffpe-dataset/) [15]. Third, single-cell data from lung cancer patients were downloaded from GSE131907 [13]. Lastly, spatial and single-cell data from breast cancer patients were downloaded from https://www.ncbi.nlm.nih.gov/geo/query/acc.cgi?acc=GSE176078 and https://zenodo.org/record/4739739 [17, 18]. Source codes (in Python and R) and an application for STopover are uploaded to https://github.com/bsungwoo/STopover [64].

## Abbreviations

ADC: Adenocarcinoma
BMP: Bone morphogenic protein
CAF: Cancer-associated fibroblast
CC: Connected component
DDR1: Discoidin domain receptor 1
ECM: Extracellular matrix
EFNA: Ephrin A
ER: Estrogen receptor
FC: Fold change
FFPE: Formalin-fixed paraffin-embedded
FOV: Field of view
FWHM: Full-width half maximum
GO: Gene Ontology
ICI: Immune checkpoint inhibitor
IDC: Invasive ductal carcinoma
ILC: Invasive lobular carcinoma
LR: Ligand-receptor
MHC: Major histocompatibility complex
OCT: Optimal cutting temperature
PR: Progesterone receptor
PVL: Perivascular-like cells
SRT: Spatially resolved transcriptomics
tS2: Tumor epithelial cell type defined in lung ADC dataset related to tumor progression
TME: Tumor microenvironment
TNBC: Triple-negative breast cancer
Treg: Regulatory T cells
