# Supplementary material for: STopover captures spatial colocalization and interaction in the tumor microenvironment using topological analysis in spatial transcriptomics data
Source: Genome Med. 2025 Apr 1;17:33. doi: 10.1186/s13073-025-01457-1 (PMC11963362; doi:10.1186/s13073-025-01457-1)
Supplement: Supplementary file 1 — Additional file 1: Supplementary Figures. Fig. S1. Schematic image for converting image-based SRT to grid-based data. Fig. S2. Extracting CCs by Morse filtration and dendrogram smoothing in Visium dataset. Fig. S3. Parameter optimization for STopover. Fig. S4. Aggregated CCs obtained across various parameters in simulated dataset. Fig. S5. Calculation of weighted Jaccard index from the extracted CC pairs in the simulation dataset. Fig. S6. Spatial distribution of tS2 and other main cell types composing the lung cancer tissue in barcode-based SRT. Fig. S7. The location of the top CC pairs between tS2 and NK/T cells in a PD-L1 high lung cancer tissue. Fig. S8. Colocalization patterns between tS2 and multiple T cell subtypes in barcode-based SRT of lung cancer. Fig. S9. Scatter plots between pseudobulk RNA expression and Jcomp value of tS2 and NK/T cells. Fig. S10. Comparison of set-based and weighted Jaccard index across 11 barcode-based SRT of lung cancer. Fig. S11. Spatial expression of the top 3 ligand-receptor pairs in PD-L1 high lung cancer tissue. Fig. S12. The spatial overlap patterns of the top ligand-receptorpairs constrained to the tS2 or myeloid cell domain in PD-L1 high lung cancer tissue. Fig. S13. The common top ligand-receptorinteractions in Clusters 1 and 2 defined by infiltration patterns of immune and stromal cells to tumor cells. Fig. S14. The top ligand-receptorinteractions estimated by STopover using the Omnipath database. Fig. S15. The spatial overlap patterns of the T cell action-related LR pairs constrained to the tS2 and NK/T cell colocalized domain in PD-L1 high lung cancer tissue. Fig. S16. Spatial distribution of tS2 and other main cell types composing the lung cancer tissue in image-based SRT. Fig. S17. The colocalization patterns between tS2 and multiple T cell subtypes in image-based SRT of lung cancer. Fig. S18. Spatial distribution of the top ligand-receptor expression specific to tS2 and NK/T cells. Fig. S19. The top differentially up [file 13073_2025_1457_MOESM1_ESM.pdf]

Additional file 1: Supplementary Figures

STopover captures spatial colocalization and interaction in the tumor microenvironment using topological analysis in spatial transcriptomics data

Sungwoo Bae[1,†], Hyekyoung Lee[2, †], Kwon Joong Na[3], Dong Soo Lee[2,4], Hongyoon Choi[2,4,5*], Young Tae Kim[3,*]

[1]Institute of Radiation Medicine, Medical Research Center, Seoul National University, Seoul, Republic of Korea.; [2]Department of Nuclear Medicine, Seoul National University Hospital, Seoul, Republic of Korea.; [3]Department of Thoracic and Cardiovascular Surgery, Seoul National University Hospital, Seoul, Republic of Korea.; [4]Department of Nuclear Medicine, Seoul National University College of Medicine, Republic of Korea.; [5]Portrai, Inc. Republic of Korea

[†]: The first two authors contributed equally to this work.

Correspondence

Hongyoon Choi, MD., Ph.D.

Department of Nuclear Medicine, Seoul National University Hospital

101, Daehak-ro, Jongno-gu, Seoul 03080, Republic of Korea

Tel: +82-2-2072-2802, Fax: +82-2-745-0345, E-mail: chy1000@snu.ac.kr

Young Tae Kim, MD., Ph.D.

Department of Thoracic and Cardiovascular Surgery, Seoul National University Hospital

101 Daehak-ro, Jongno-gu, Seoul, Republic of Korea, 03080

Tel: +82-2-2072-3161, Fax: +82-2-745-0345, E-mail: ytkim@snu.ac.kr

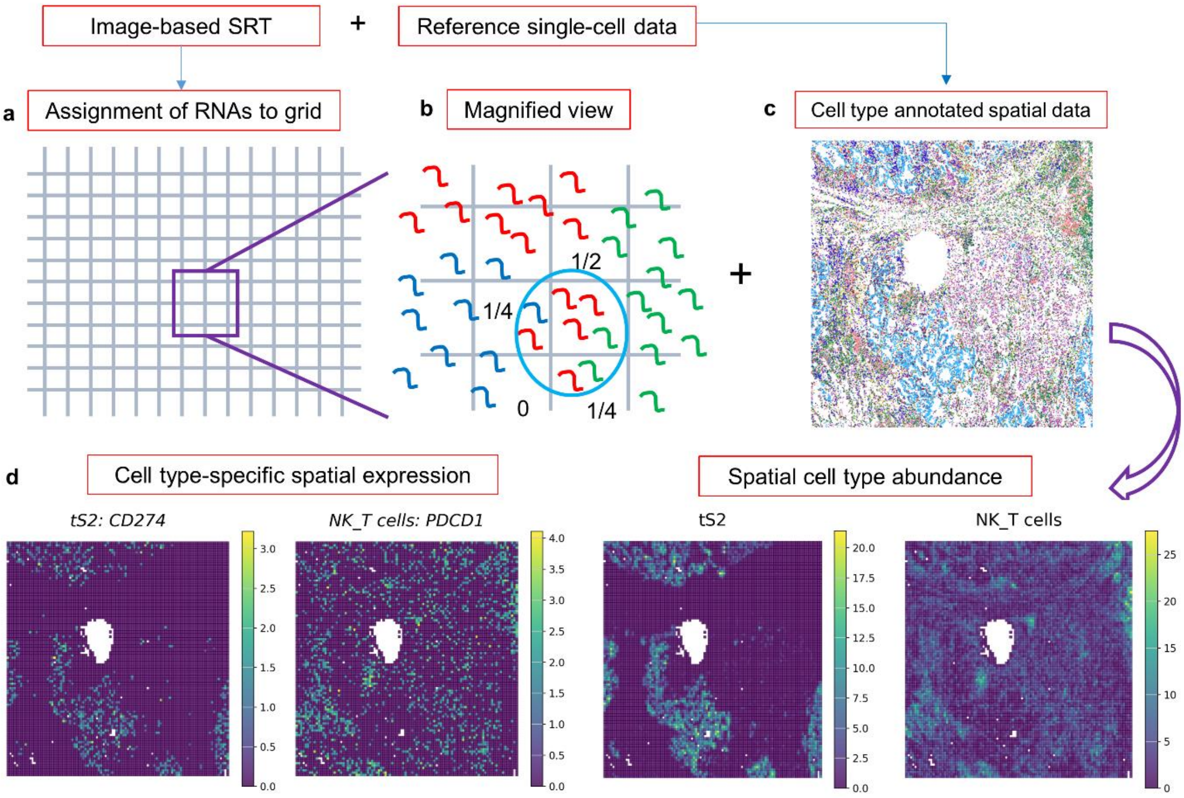

Supplementary Fig. 1. Schematic image for converting image-based SRT to grid-based data

(a) The entire field of view in the image-based SRT is divided into grids (in the case of the CosMx SMI platform, 100 by 100 grids are applied), allowing for adjustment of the spatial resolution to be similar to the Visium spatial transcriptomic dataset. **(b)** Then, RNA transcripts are assigned to the grids, and the fraction of a particular cell in a grid is calculated based on the total number of RNAs belonging to the cell in each grid that the cell covers. **(c)** Next, reference single-cell RNA-seq data is utilized to annotate the cells segmented in the image-based SRT. **(d)** By summing up the fractions of cells corresponding to a specific cell type, a spatial map of cell type abundance can be acquired. Also, cell type-specific spatial expression can be calculated by extracting RNAs belonging to a specific cell type.

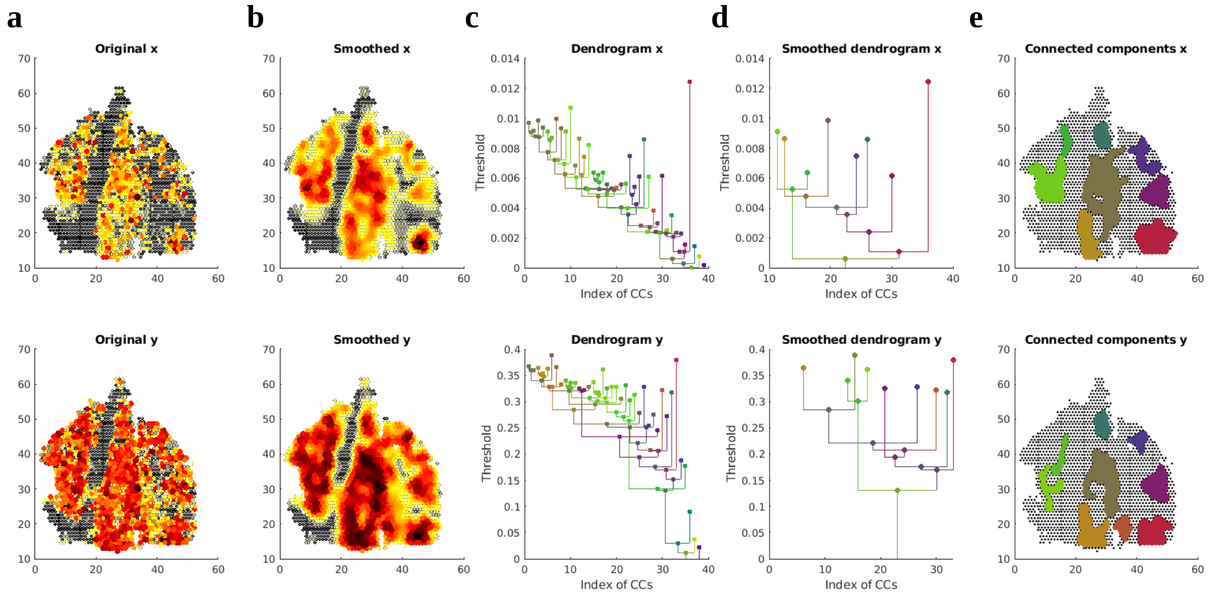

Supplementary Fig. 2. Extracting CCs by Morse filtration and dendrogram smoothing in Visium dataset.

An example of extracting connected components (CCs) from a spatial map of feature x (tS2) and feature y (NK/T cells), which is the core algorithm of STopover. The utilized Visium dataset is one of the PD-L1 high tissues (spa18ca02). **(a, b)** First, the original spatial maps of the features are smoothed by applying a Gaussian filter with a full-width half maximum (FWHM) of 2.5 times the unit distance between the center of the spots. **(c)** Then, the CCs of both features are discovered based on the spatial distance between spots by gradually lowering the feature values from the highest possible threshold value to the lowest value. **(d)** The resulting CCs, expressed as dendrograms, are smoothed based on the minimum size of the CCs and the persistence of each CC during the threshold lowering. **(e)** Finally, reconfigured CCs, represented by the uppermost bars of the dendrograms, are considered to be locally activated regions of the feature x and y.

To remove the noise of the spatial map, the CCs with the feature value below the certain percentile threshold are removed, and the local and composite Jaccard indexes are calculated

between the final CCs of the feature pair (features x and y).

(a)

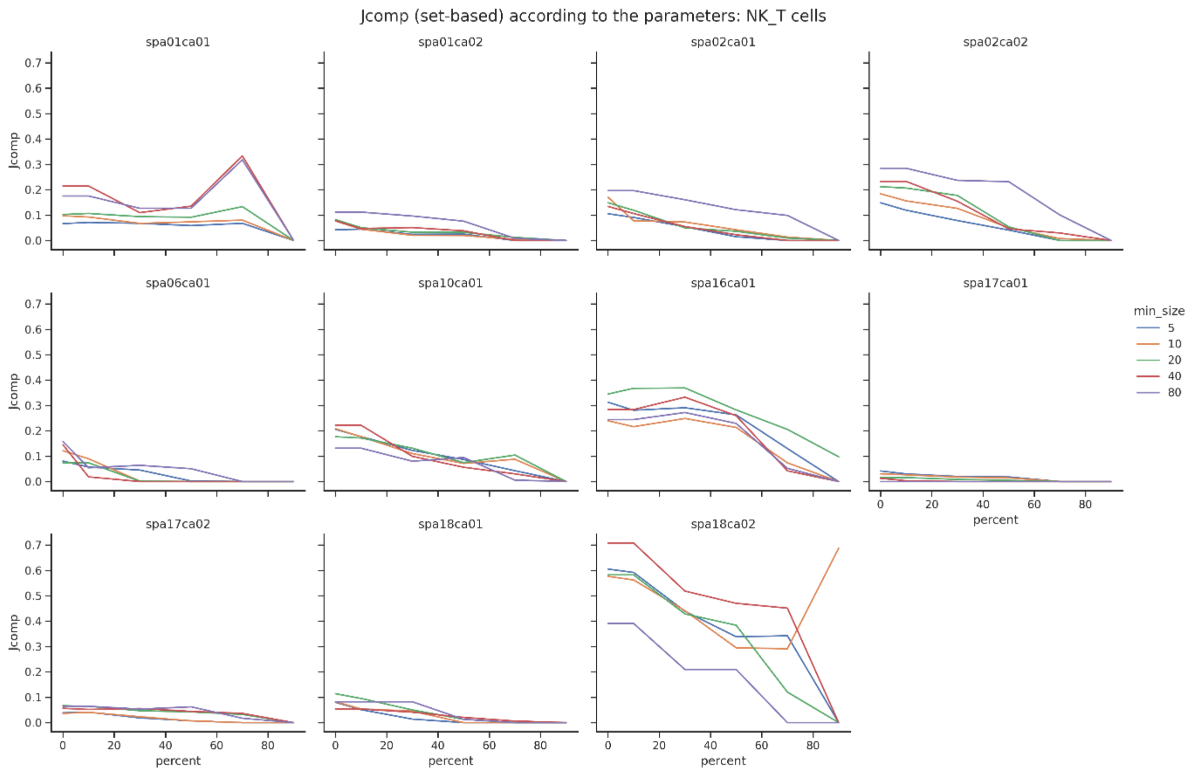
Jcomp (set-based) according to the parameters: NK_T cells
spa01ca01
spa01ca02
spa02ca01
spa02ca02
spa06ca01
spa10ca01
spa16ca01
spa17ca01
spa17ca02
spa18ca01
spa18ca02
Jcomp
percent
min_size
5
10
20
40
80

(b)

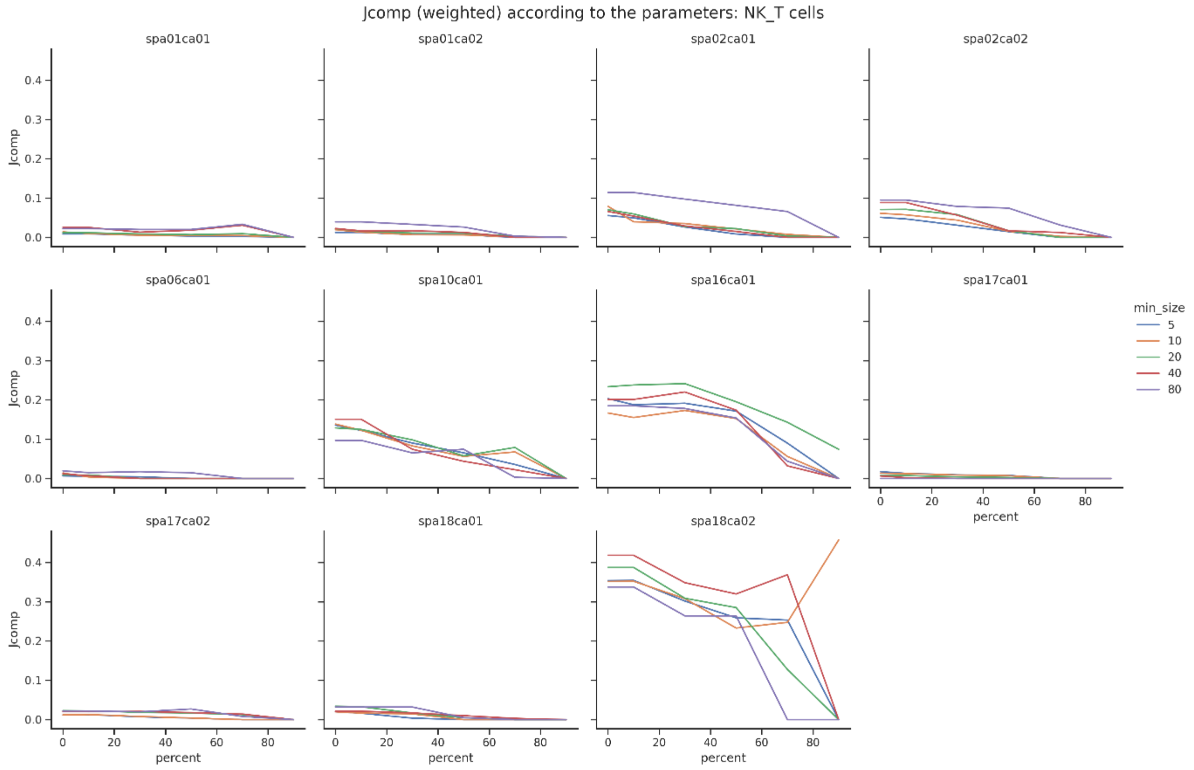
Jcomp (weighted) according to the parameters: NK_T cells
spa01ca01
spa01ca02
spa02ca01
spa02ca02
spa06ca01
spa10ca01
spa16ca01
spa17ca01
spa17ca02
spa18ca01
spa18ca02
Jcomp
percent
min_size
5
10
20
40
80

(c)

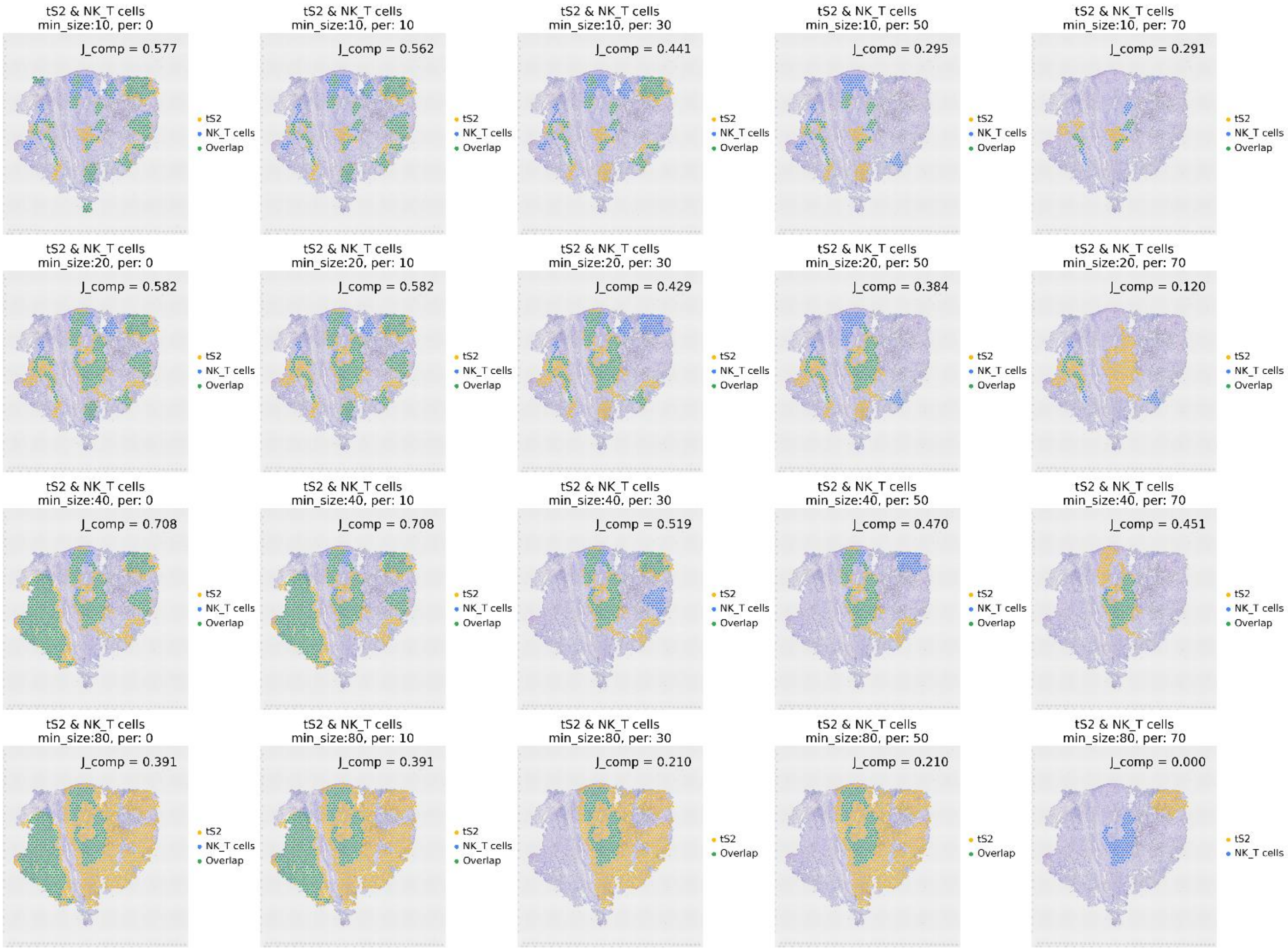
tS2 & NK_T cells
min_size:10, per: 0
J_comp = 0.577
tS2 & NK_T cells
min_size:10, per: 10
J_comp = 0.562
tS2 & NK_T cells
min_size:10, per: 30
J_comp = 0.441
tS2 & NK_T cells
min_size:10, per: 50
J_comp = 0.295
tS2 & NK_T cells
min_size:10, per: 70
J_comp = 0.291
tS2 & NK_T cells
min_size:20, per: 0
J_comp = 0.582
tS2 & NK_T cells
min_size:20, per: 10
J_comp = 0.582
tS2 & NK_T cells
min_size:20, per: 30
J_comp = 0.429
tS2 & NK_T cells
min_size:20, per: 50
J_comp = 0.384
tS2 & NK_T cells
min_size:20, per: 70
J_comp = 0.120
tS2 & NK_T cells
min_size:40, per: 0
J_comp = 0.708
tS2 & NK_T cells
min_size:40, per: 10
J_comp = 0.708
tS2 & NK_T cells
min_size:40, per: 30
J_comp = 0.519
tS2 & NK_T cells
min_size:40, per: 50
J_comp = 0.470
tS2 & NK_T cells
min_size:40, per: 70
J_comp = 0.451
tS2 & NK_T cells
min_size:80, per: 0
J_comp = 0.391
tS2 & NK_T cells
min_size:80, per: 10
J_comp = 0.391
tS2 & NK_T cells
min_size:80, per: 30
J_comp = 0.210
tS2 & NK_T cells
min_size:80, per: 50
J_comp = 0.210
tS2 & NK_T cells
min_size:80, per: 70
J_comp = 0.000
tS2
NK_T cells
Overlap

(d)

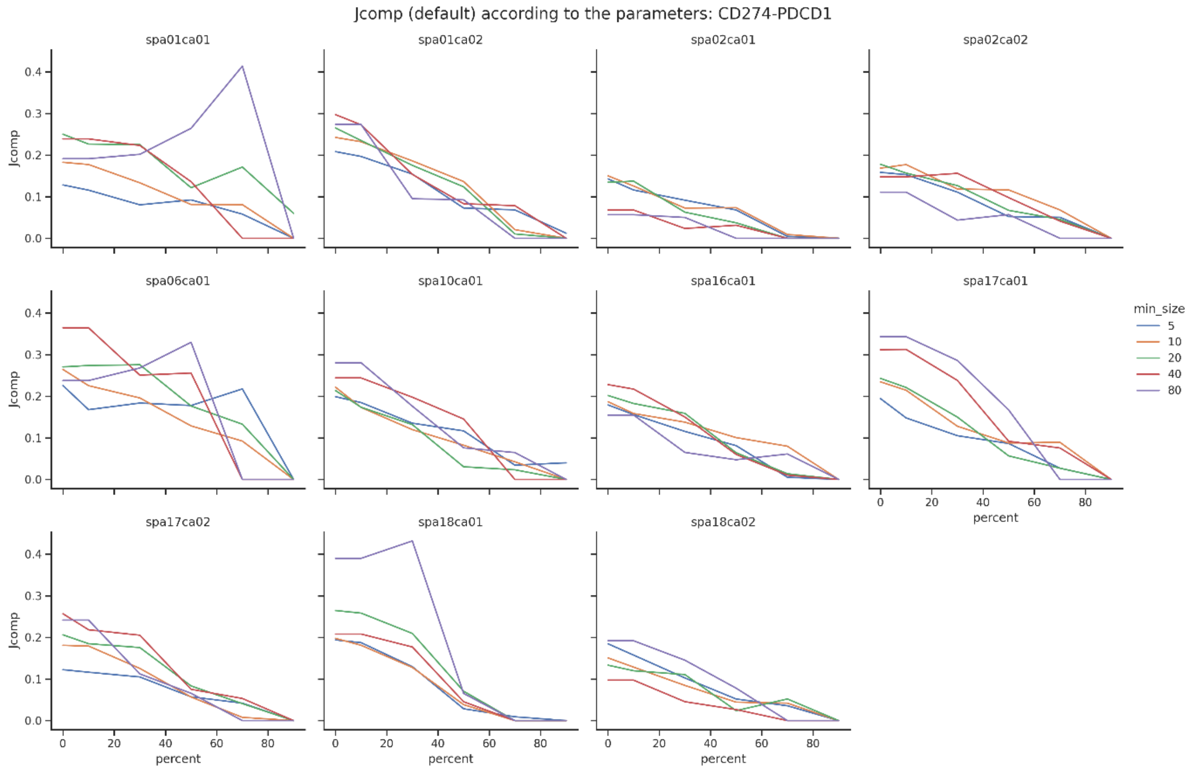

Jcomp (default) according to the parameters: CD274-PDCD1
spa01ca01
spa01ca02
spa02ca01
spa02ca02
spa06ca01
spa10ca01
spa16ca01
spa17ca01
spa17ca02
spa18ca01
spa18ca02
Jcomp
percent
min_size
5
10
20
40
80
0.0
0.1
0.2
0.3
0.4
0
20
40
60
80

(e)

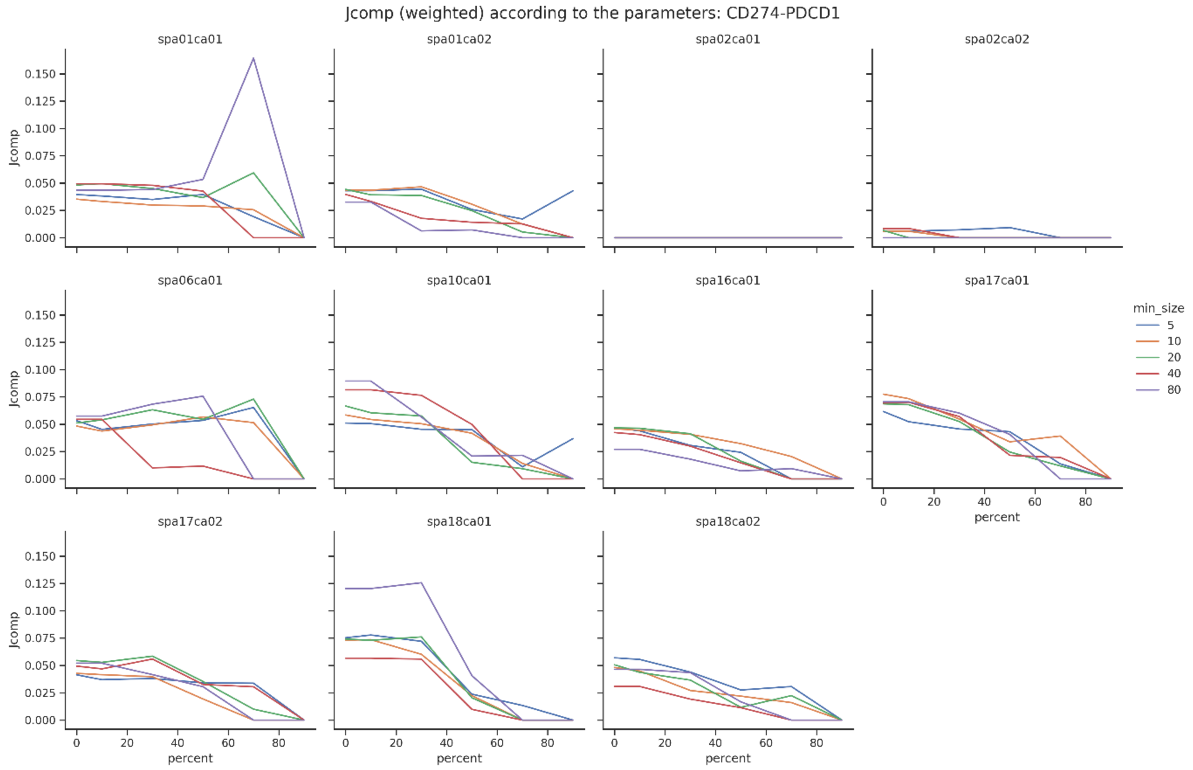

Jcomp (weighted) according to the parameters: CD274-PDCD1
spa01ca01
spa01ca02
spa02ca01
spa02ca02
spa06ca01
spa10ca01
spa16ca01
spa17ca01
spa17ca02
spa18ca01
spa18ca02
Jcomp
percent
min_size
5
10
20
40
80
0.000
0.025
0.050
0.075
0.100
0.125
0.150
0
20
40
60
80

Supplementary Fig. 3. Parameter optimization for STopover

To determine the optimal parameter range for STopover, we computed set-based and weighted Jaccard indexes between tumor cells (tS2) and NK/T cells across a barcode-based dataset comprising 11 lung cancer tissues. We systematically varied two parameters: the minimum size of connected components (smin), represented by the number of spots or grids, and the percentile threshold used to eliminate connected components with low average feature values (pt). The range of minimum connected components extended from 10 to 80 (10, 20, 40, 80), while the percentile value varied from 0 to 70 (0, 10, 30, 50, 70). The line plot visualizes the changes in **(a)** set-based and **(b)** weighted Jaccard indexes as these parameters were adjusted.

(c) In one of the barcode-based SRT datasets of lung cancer (spa18ca02), we calculated the aggregated connected components between tS2 and NK/T cells while varying these two parameters. The set-based Jcomp values are displayed in the top right corner of the plot.

Additionally, we evaluated the optimal parameter range for STopover when applied to the ligand-receptor pair, *CD274-PDCD1*, which exhibits sparse expression patterns. The line plot depicts the changes in **(d)** set-based and **(e)** weighted Jaccard indexes as parameters were adjusted in this specific context.

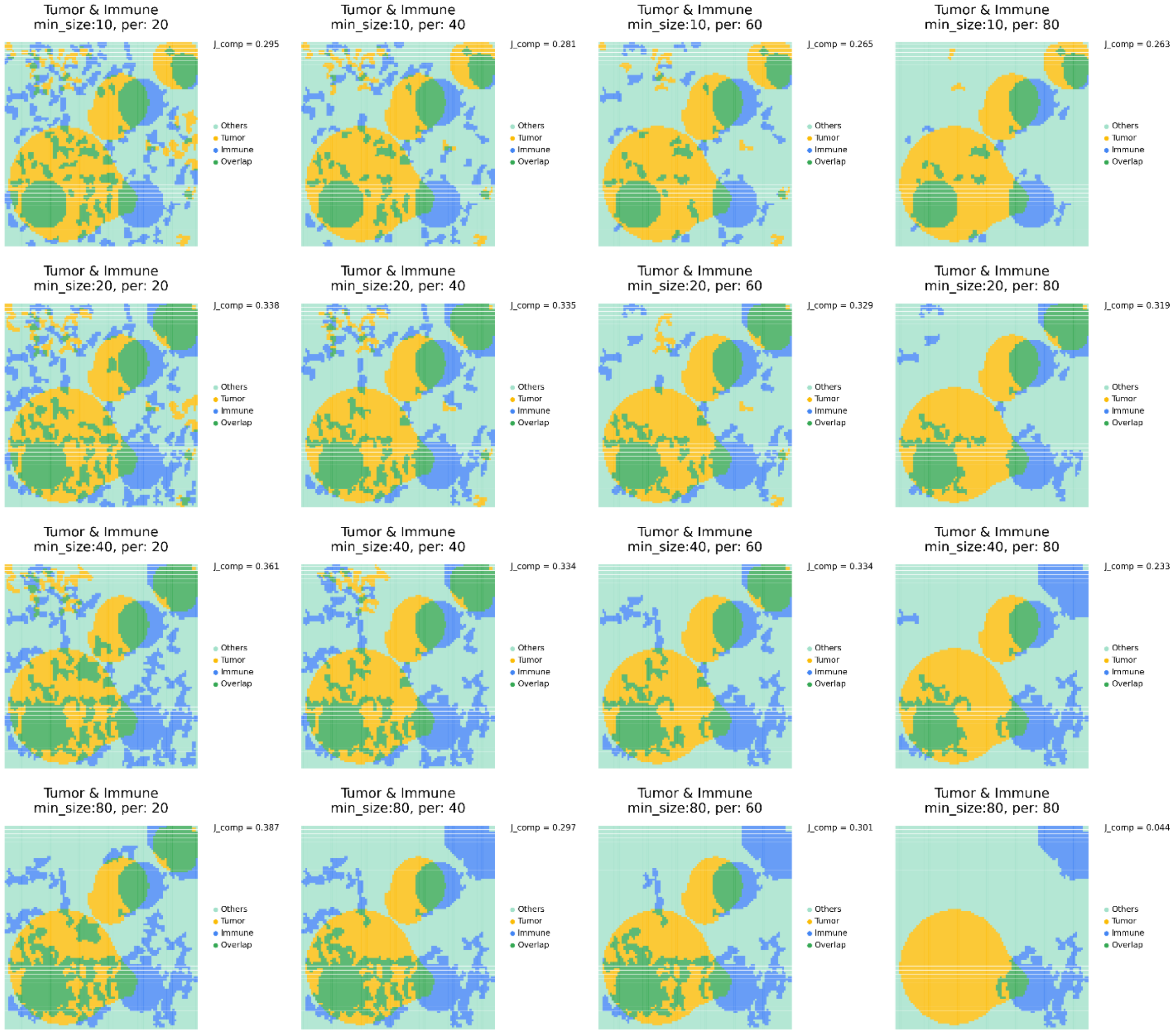

Supplementary Fig. 4. Aggregated CCs obtained across various parameters in simulated dataset

The aggregated CCs were computed between the simulated tumor and immune distributions by changing two parameters: the minimum size of CCs, represented by the number of spots or grids, and the percentile threshold for removing the CCs with low average feature values. The minimum number of CCs was varied from 10 to 80 (10, 20, 40, 80), and the percentile value was varied from 0 to 70 (0, 10, 30, 50, 70). The set-based J_{comp} values are displayed in the top right corner of the plot.

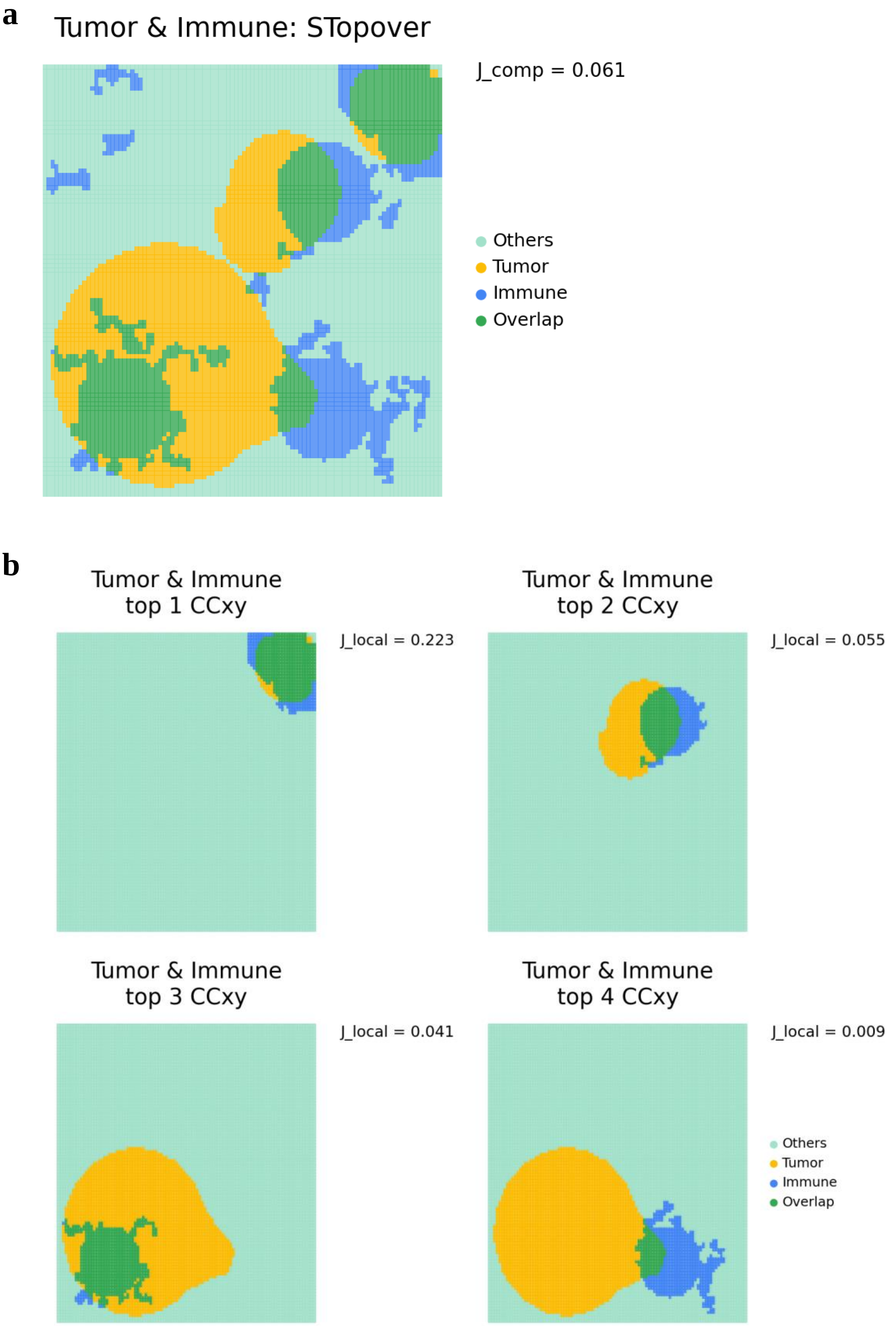
a
Tumor & Immune: STopover
J_comp = 0.061
Others
Tumor
Immune
Overlap
b
Tumor & Immune
top 1 CCxy
J_local = 0.223
Tumor & Immune
top 2 CCxy
J_local = 0.055
Tumor & Immune
top 3 CCxy
J_local = 0.041
Tumor & Immune
top 4 CCxy
J_local = 0.009
Others
Tumor
Immune
Overlap

Supplementary Fig. 5. Calculation of weighted Jaccard index from the extracted CC pairs in the simulation dataset.

(a) Visualization of the connected components (CCs) for tumor cells (yellow) and immune cells (blue), along with the intersecting subregions (green) between the two combined CCs on the grid. The weighted Jaccard index was computed between these combined CCs (J_{comp}).

(b) Visualization of the top 4 CC pairs with the highest spatial overlap, as indicated by the J_{local} score, between tumor and immune cells. CCs from tumor cells (yellow), immune cells (blue), and intersecting subregions (green) are displayed on the grid. The weighted Jaccard indexes were computed for each CC pair.

a **PD-L1 low tissue (*spa06ca01*)**

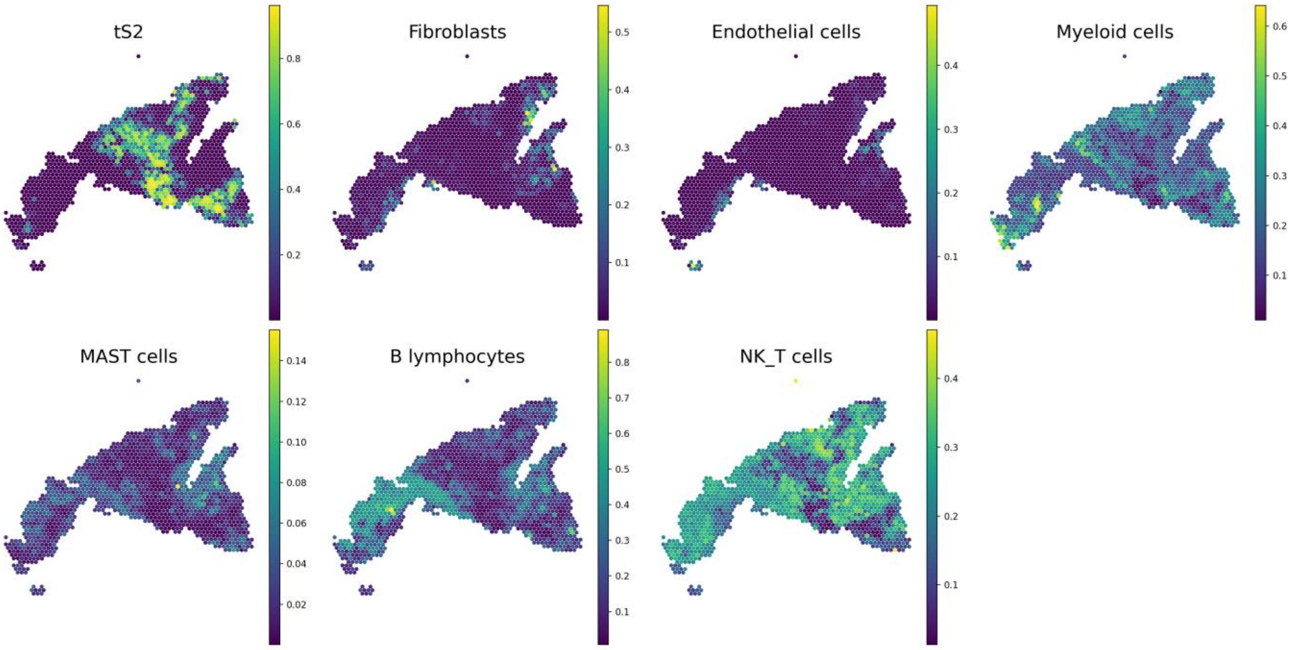

b **PD-L1 high tissue (*spa18ca02*)**

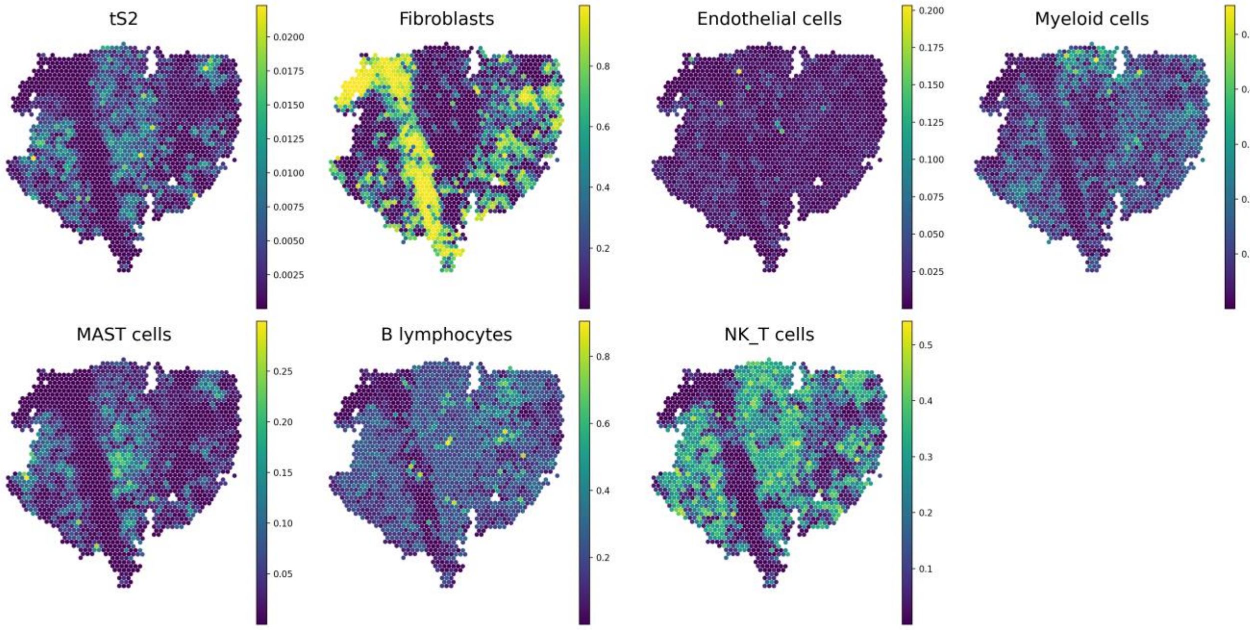

Supplementary Fig. 6. Spatial distribution of tS2 and other main cell types composing the lung cancer tissue in barcode-based SRT.

The spatial composition of tS2 and other main cell types (fibroblasts, endothelial cells, myeloid cells, MAST cells, B lymphocytes, and NK/T cells), in representative **(a)** PD-L1 low (*spa6ca01*) and **(b)** high tissues (*spa18ca02*) were estimated and visualized.

a

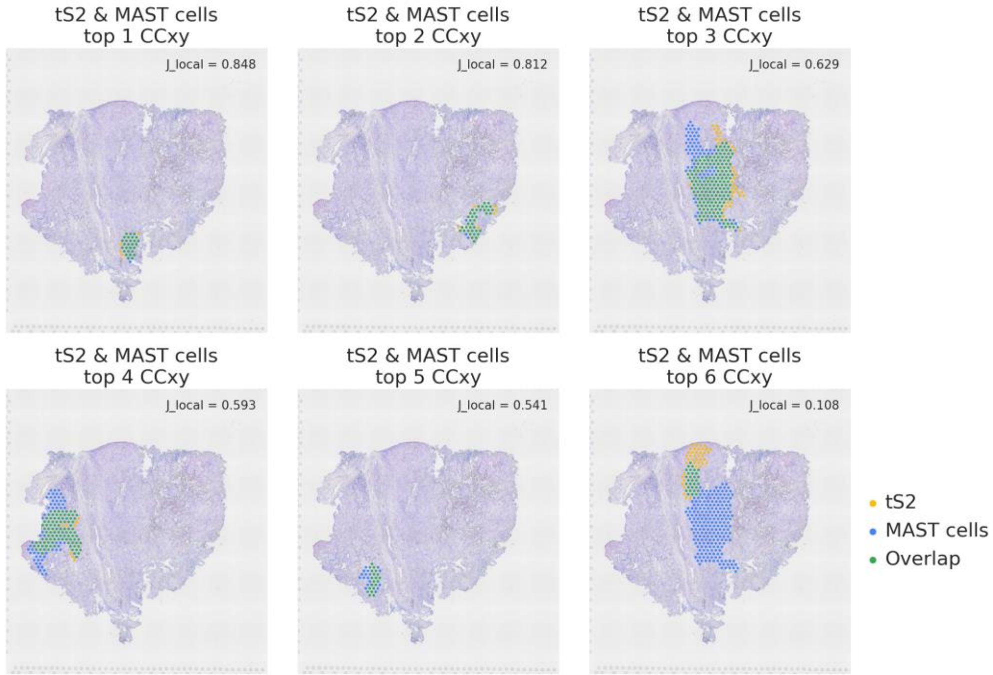
tS2 & MAST cells
top 1 CCxy
J_local = 0.848
tS2 & MAST cells
top 2 CCxy
J_local = 0.812
tS2 & MAST cells
top 3 CCxy
J_local = 0.629
tS2 & MAST cells
top 4 CCxy
J_local = 0.593
tS2 & MAST cells
top 5 CCxy
J_local = 0.541
tS2 & MAST cells
top 6 CCxy
J_local = 0.108
tS2
MAST cells
Overlap

b

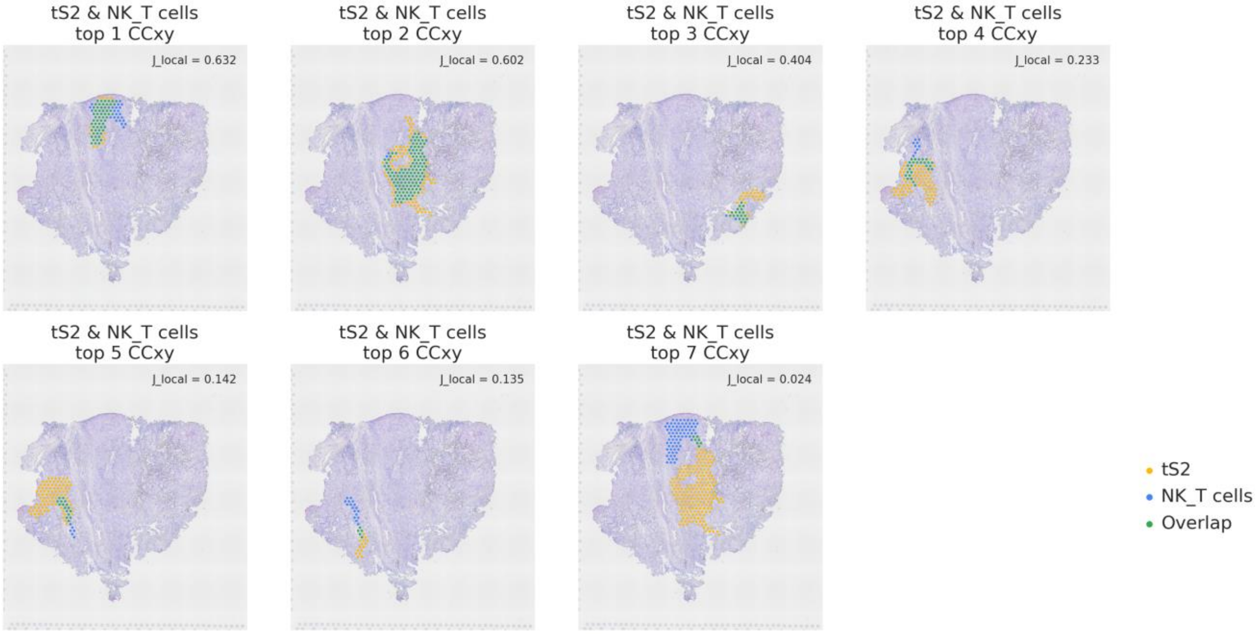
tS2 & NK_T cells
top 1 CCxy
J_local = 0.632
tS2 & NK_T cells
top 2 CCxy
J_local = 0.602
tS2 & NK_T cells
top 3 CCxy
J_local = 0.404
tS2 & NK_T cells
top 4 CCxy
J_local = 0.233
tS2 & NK_T cells
top 5 CCxy
J_local = 0.142
tS2 & NK_T cells
top 6 CCxy
J_local = 0.135
tS2 & NK_T cells
top 7 CCxy
J_local = 0.024
tS2
NK_T cells
Overlap

Supplementary Fig. 7. The location of the top CC pairs between tS2 and NK/T cells in a PD-L1 high lung cancer tissue (*spa18ca02*)

The pairs of CCs between tS2 and either **(a)** MAST cells or **(b)** NK/T cells were extracted using STopover. The top pairs with the highest J_{local} were then visualized in the descending order of J_{local} values.

a **PD-L1 low tissue (*spa06ca01*)**

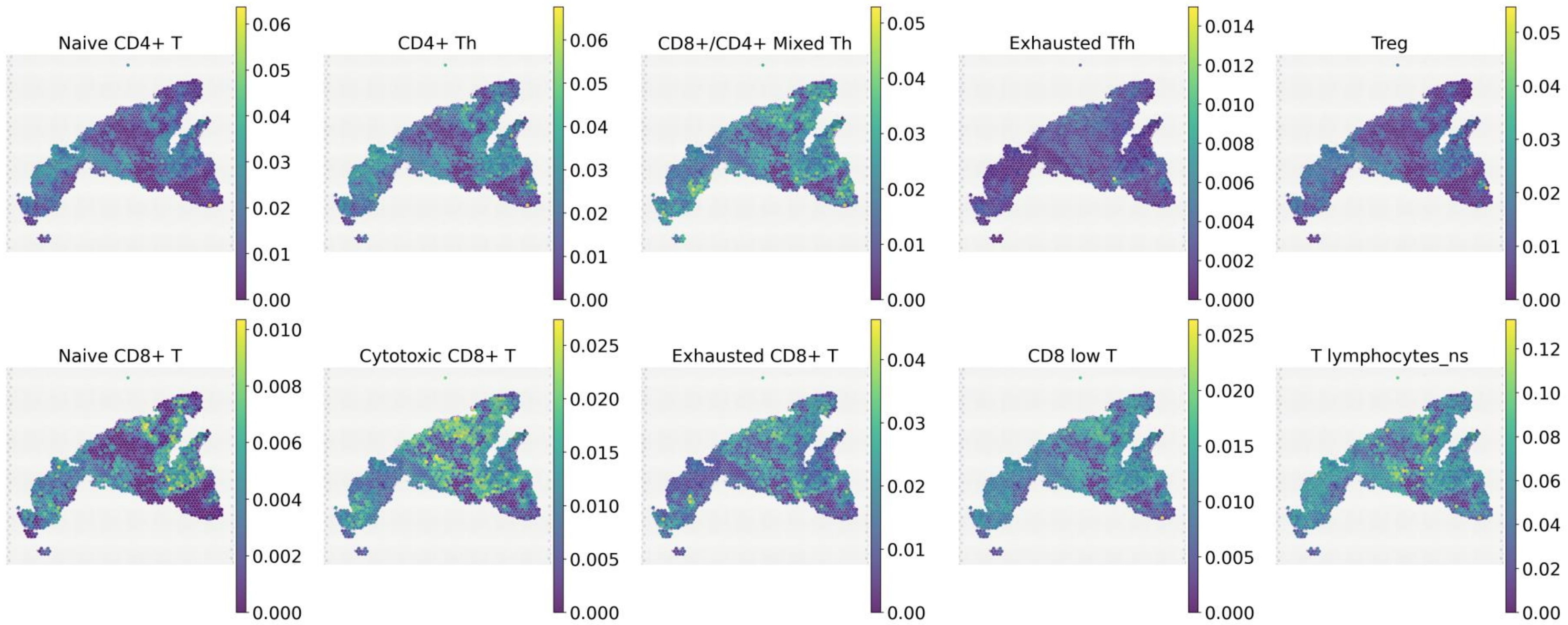

b **PD-L1 high tissue (*spa18ca02*)**

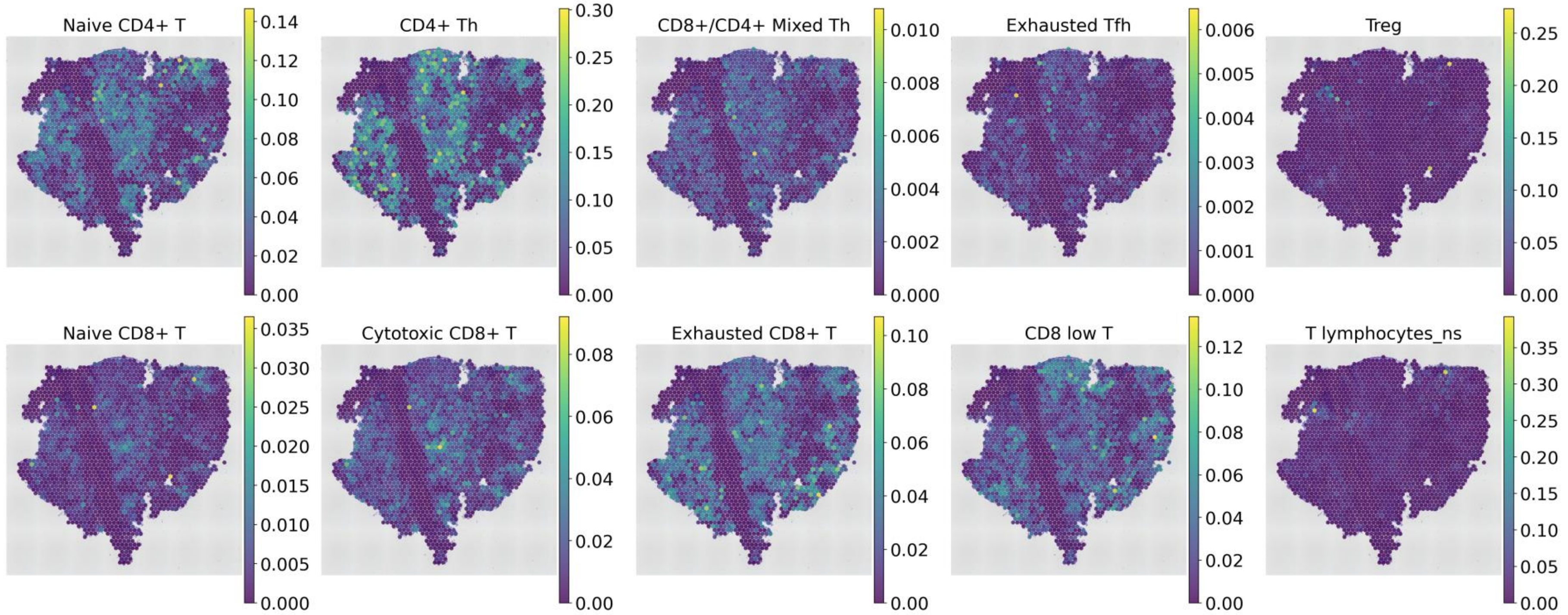

c **PD-L1 low tissue (*spa06ca01*)**

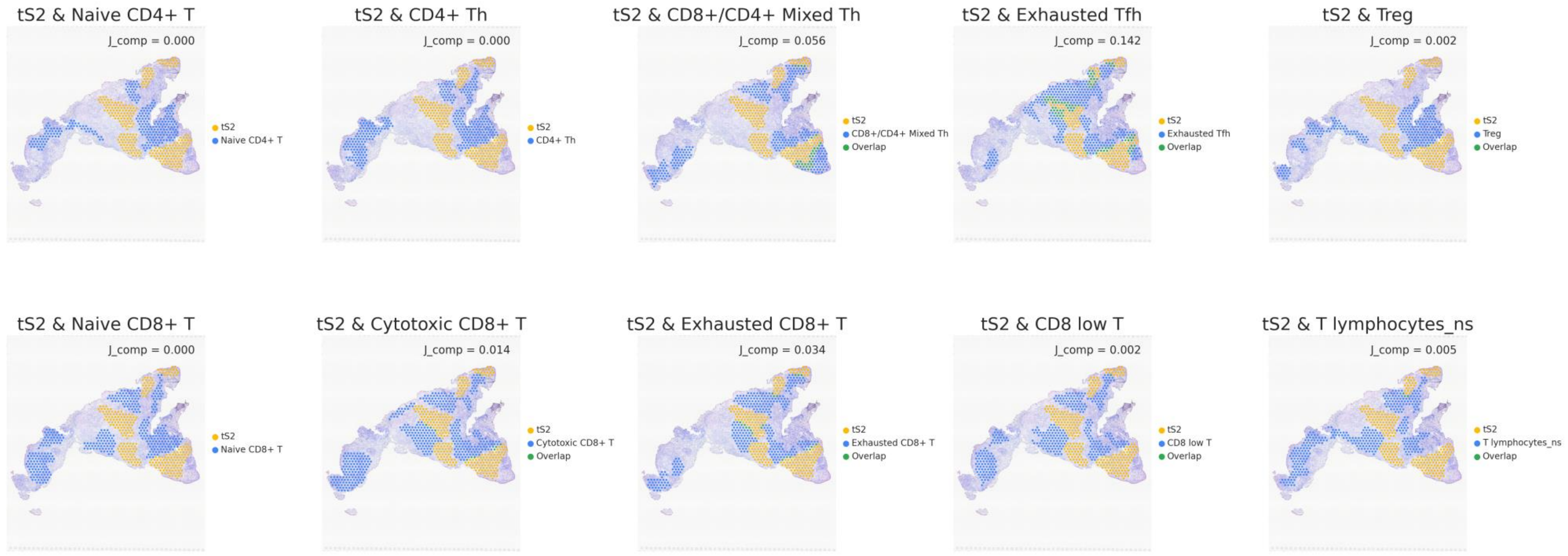

d **PD-L1 high tissue (*spa18ca02*)**

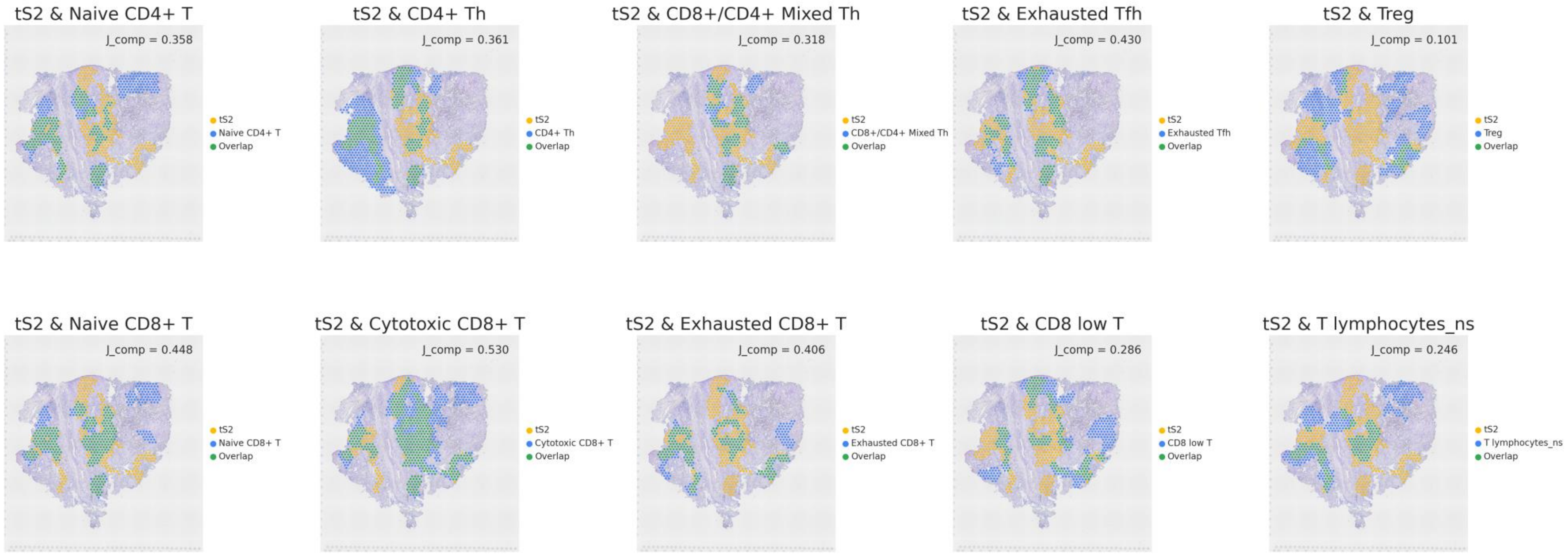

Supplementary Fig. 8. Colocalization patterns between tS2 and multiple T cell subtypes in barcode-based SRT of lung cancer

The spatial composition of multiple T cell subtypes, in representative **(a)** PD-L1 low (*spa6ca01*) and **(b)** high tissues (*spa18ca02*) were estimated and visualized. Then, the key location of tS2 and multiple T cells subtypes were represented by CCs, and the overlapping tissue domains were highlighted as the intersecting subregions between the two aggregated CCs. The analysis was performed in the two representative **(c)** PD-L1 low (*spa6ca01*) and **(d)** high tissues (*spa18ca02*).

a

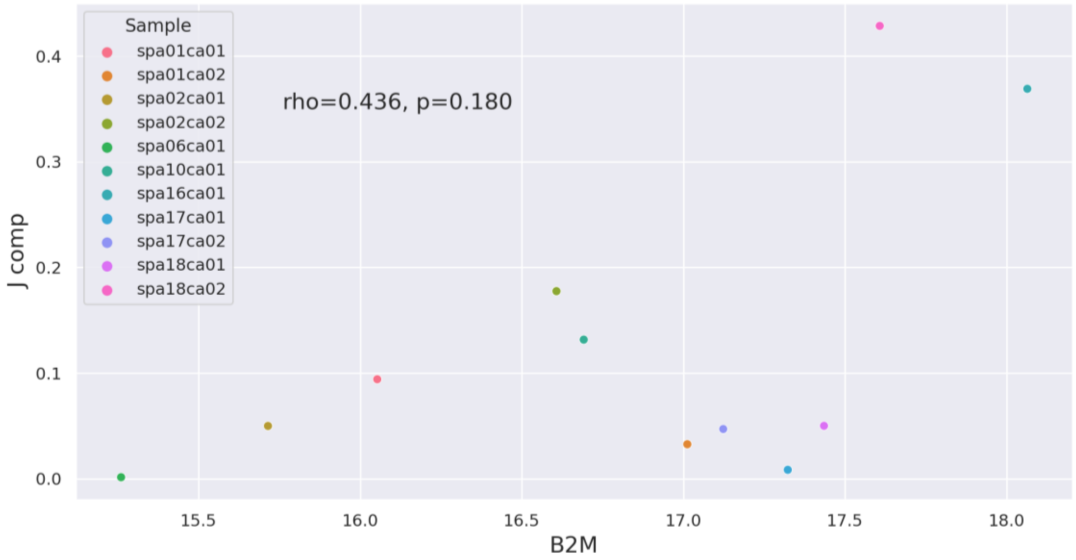
Sample
spa01ca01
spa01ca02
spa02ca01
spa02ca02
spa06ca01
spa10ca01
spa16ca01
spa17ca01
spa17ca02
spa18ca01
spa18ca02
rho=0.436, p=0.180
J comp
0.0
0.1
0.2
0.3
0.4
15.5
16.0
16.5
17.0
17.5
18.0
B2M

b

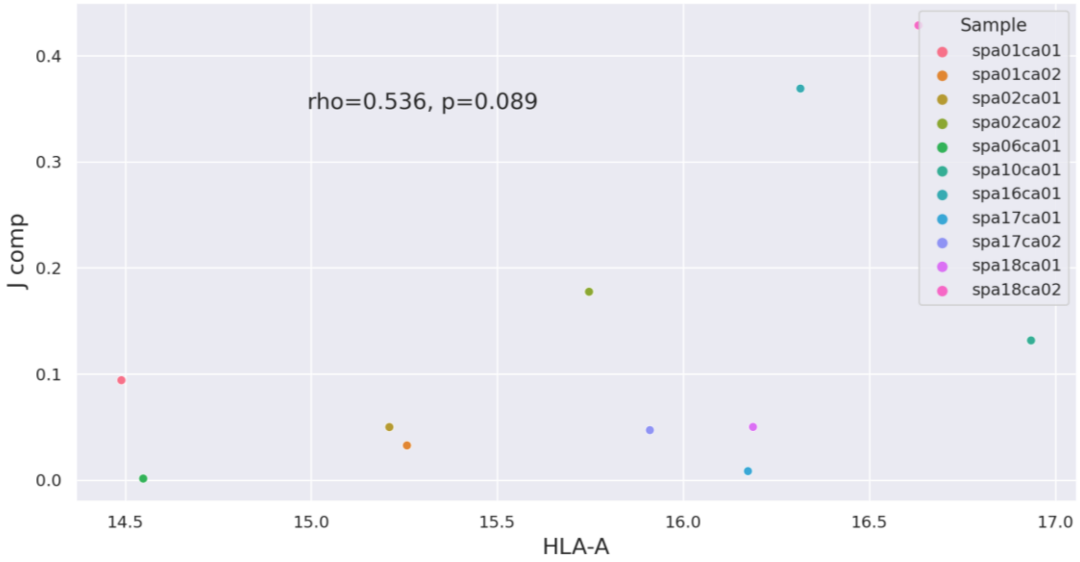
Sample
spa01ca01
spa01ca02
spa02ca01
spa02ca02
spa06ca01
spa10ca01
spa16ca01
spa17ca01
spa17ca02
spa18ca01
spa18ca02
rho=0.536, p=0.089
J comp
0.0
0.1
0.2
0.3
0.4
14.5
15.0
15.5
16.0
16.5
17.0
HLA-A

c

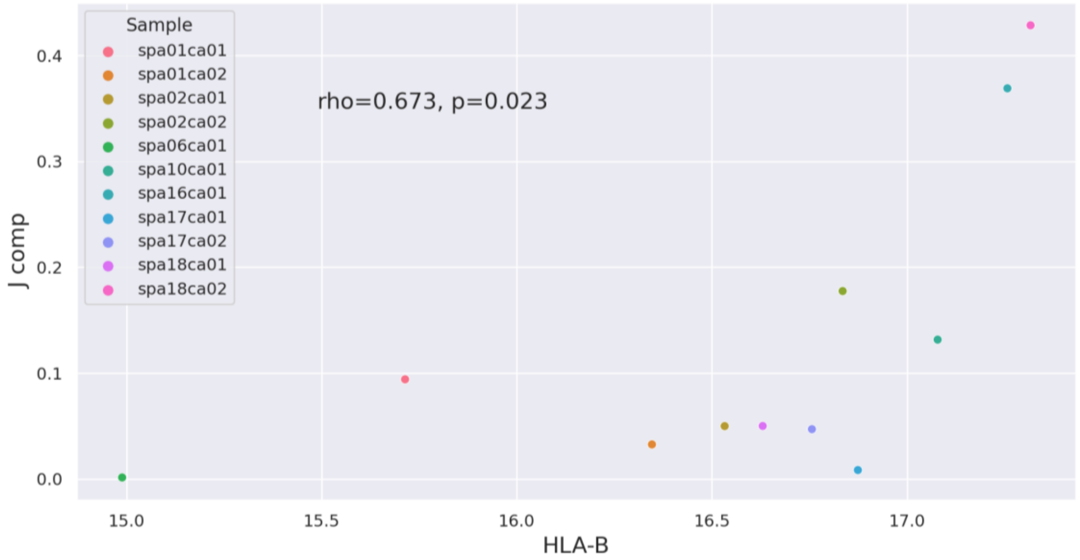
Sample
spa01ca01
spa01ca02
spa02ca01
spa02ca02
spa06ca01
spa10ca01
spa16ca01
spa17ca01
spa17ca02
spa18ca01
spa18ca02
rho=0.673, p=0.023
J comp
0.0
0.1
0.2
0.3
0.4
15.0
15.5
16.0
16.5
17.0
HLA-B

d

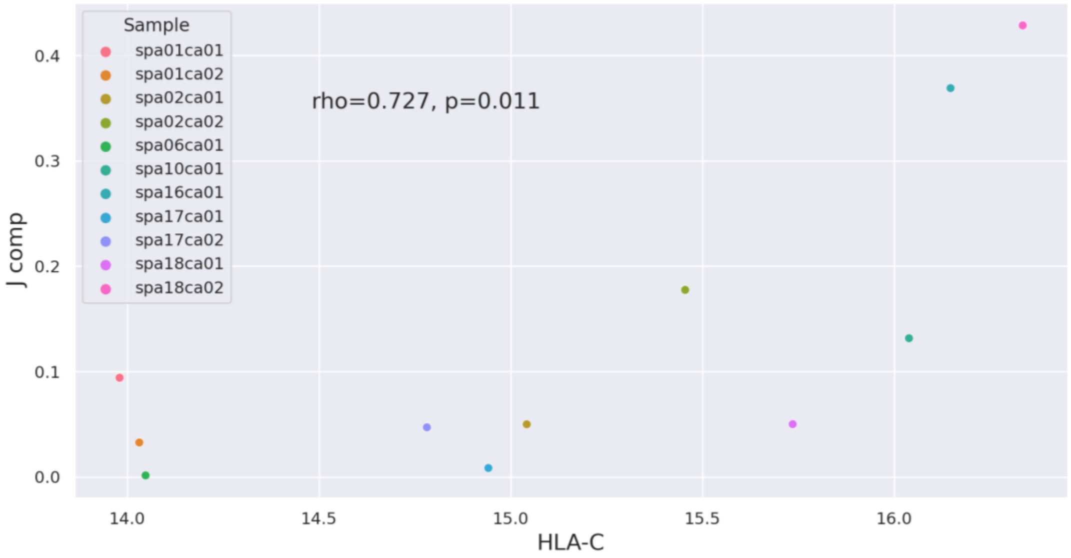
Sample
spa01ca01
spa01ca02
spa02ca01
spa02ca02
spa06ca01
spa10ca01
spa16ca01
spa17ca01
spa17ca02
spa18ca01
spa18ca02
rho=0.727, p=0.011
J comp
0.0
0.1
0.2
0.3
0.4
14.0
14.5
15.0
15.5
16.0
HLA-C

e

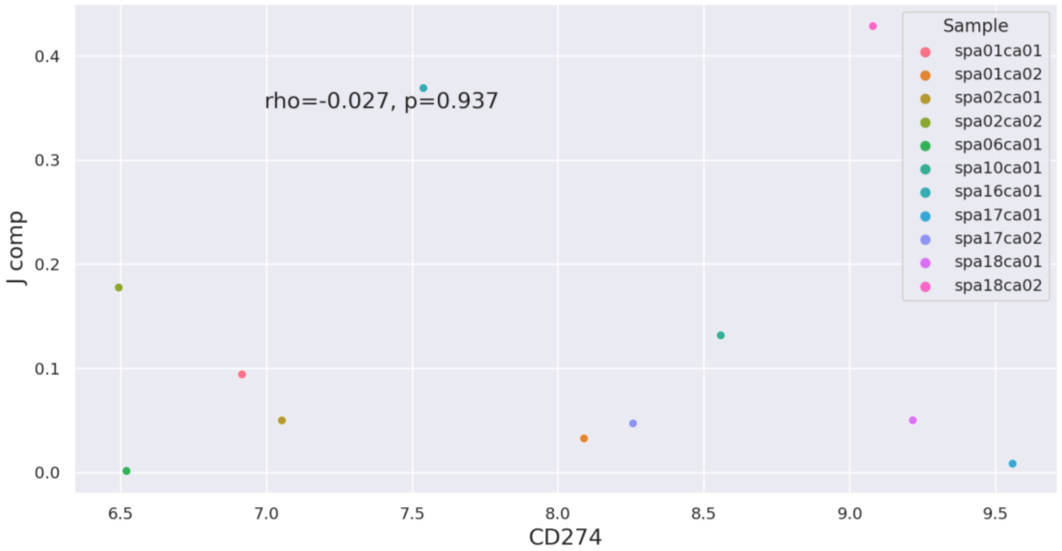
Sample
spa01ca01
spa01ca02
spa02ca01
spa02ca02
spa06ca01
spa10ca01
spa16ca01
spa17ca01
spa17ca02
spa18ca01
spa18ca02
rho=-0.027, p=0.937
J comp
0.0
0.1
0.2
0.3
0.4
6.5
7.0
7.5
8.0
8.5
9.0
9.5
CD274

f

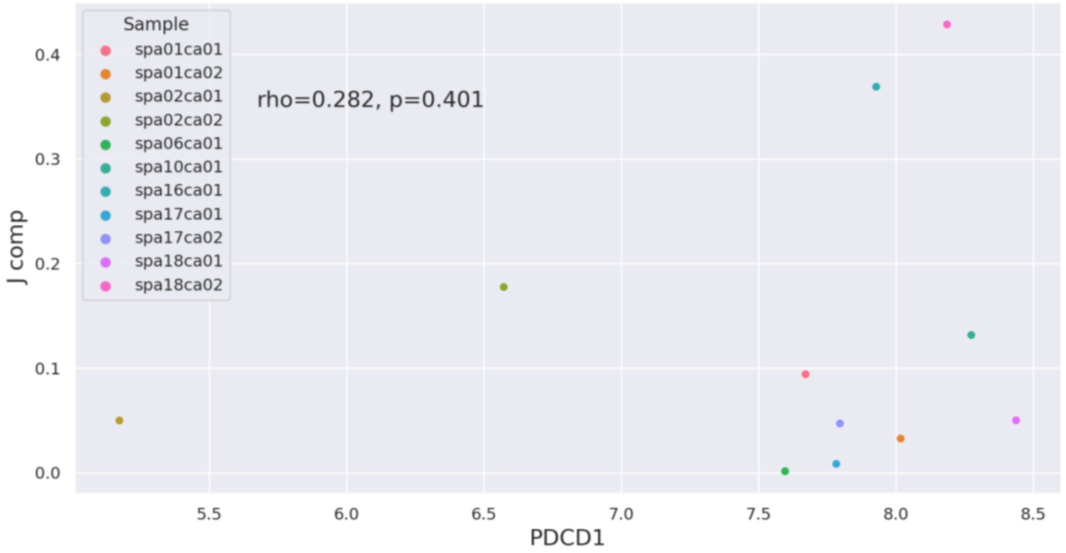
Sample
spa01ca01
spa01ca02
spa02ca01
spa02ca02
spa06ca01
spa10ca01
spa16ca01
spa17ca01
spa17ca02
spa18ca01
spa18ca02
rho=0.282, p=0.401
J comp
0.0
0.1
0.2
0.3
0.4
5.5
6.0
6.5
7.0
7.5
8.0
8.5
PDCD1

Supplementary Fig. 9. Scatter plots between pseudobulk RNA expression and J_{comp} value of tS2 and NK/T cells

The scatter plot displays the correlation between pseudobulk RNA expression of **(a)** B2M, **(b)** HLA-A, **(c)** HLA-B, **(d)** HLA-C, **(e)** CD274, and **(f)** PDCD1, which were calculated by log-transforming the total RNA counts, and the J_{comp} value, which represents the extent of spatial overlap between tS2 and NK/T cells. The plot visualizes the data points obtained from 11 lung cancer tissues. The Spearman's correlation coefficient is calculated and shown in the top right corner of each plot.

(a)

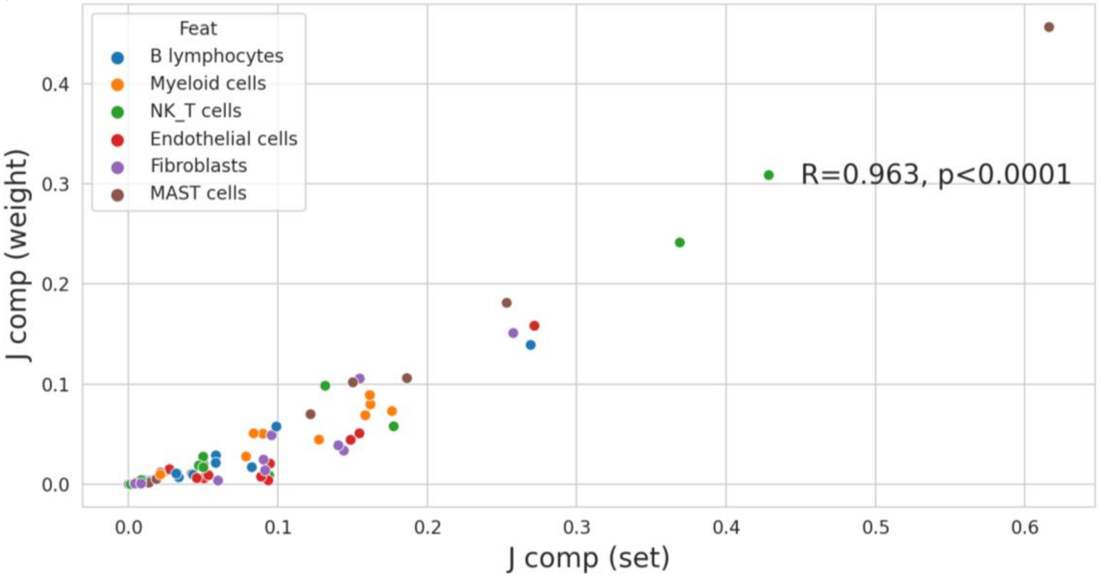

Feat
B lymphocytes
Myeloid cells
NK_T cells
Endothelial cells
Fibroblasts
MAST cells
R=0.963, p<0.0001
J comp (weight)
J comp (set)
0.0
0.1
0.2
0.3
0.4
0.5
0.6

(b)

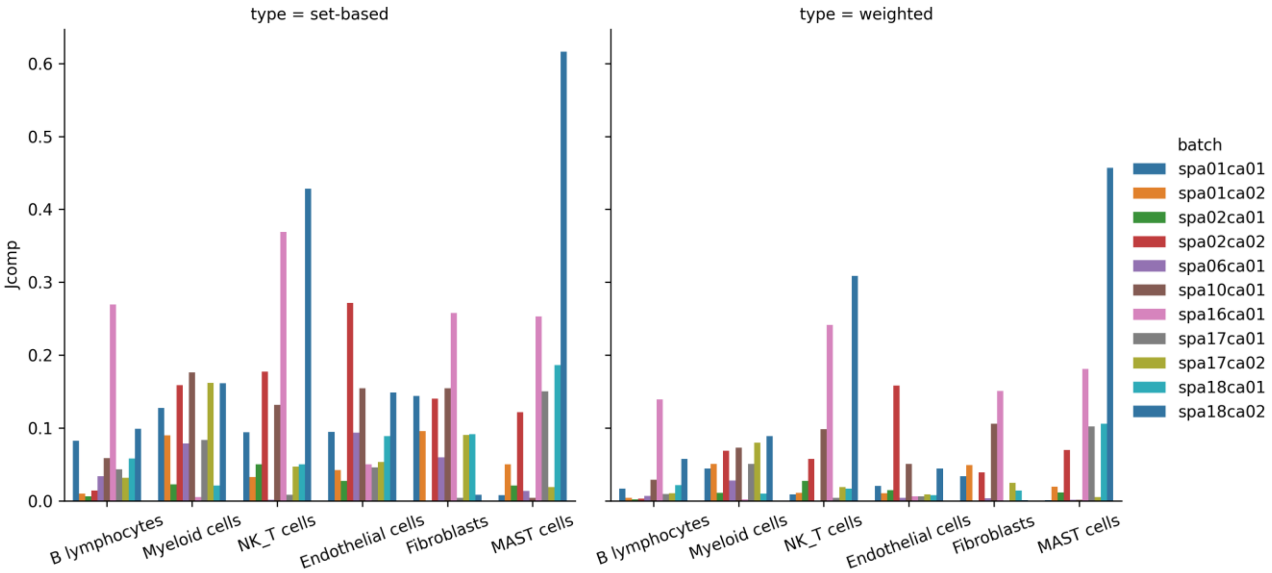

type = set-based
type = weighted
Jcomp
B lymphocytes
Myeloid cells
NK_T cells
Endothelial cells
Fibroblasts
MAST cells
batch
spa01ca01
spa01ca02
spa02ca01
spa02ca02
spa06ca01
spa10ca01
spa16ca01
spa17ca01
spa17ca02
spa18ca01
spa18ca02

Supplementary Fig. 10. Comparison of set-based and weighted Jaccard index across 11 barcode-based SRT of lung cancer

(a) The scatter plot displays the correlation between set-based and weighted Jaccard index between tumor cells (tS2) and other cell types (B lymphocytes, myeloid cells, NK/T cells, endothelial cells, fibroblasts, and mast cells) in 11 lung cancer tissues.

(b) Comparison of set-based (left panel) and weighted (right panel) Jaccard indexes between tumor cells (tS2) and each cell type across 11 lung cancer tissues.

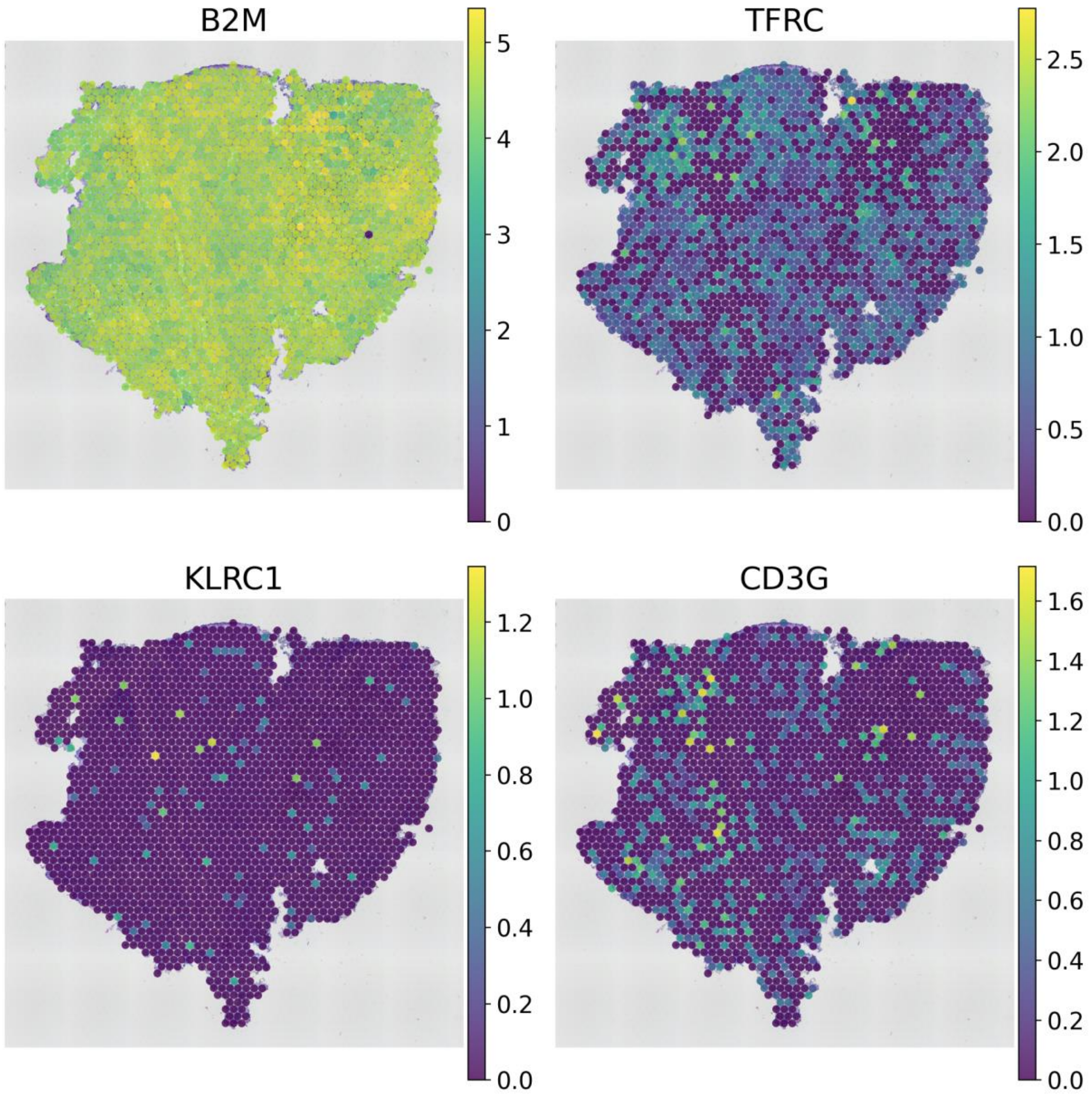

Supplementary Fig. 11. Spatial expression of the top 3 ligand-receptor pairs in PD-L1 high lung cancer tissue (*spa18ca02*)

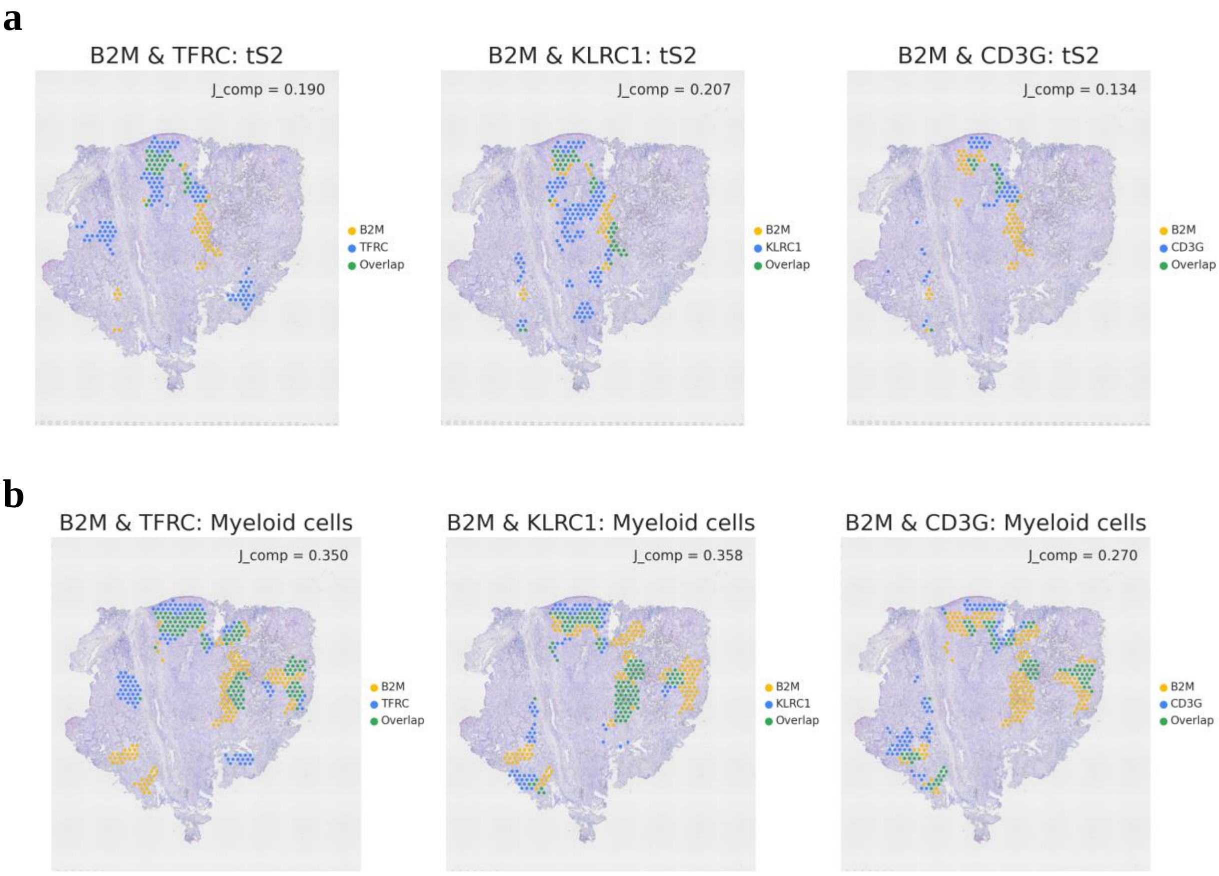

Supplementary Fig. S12. The spatial overlap patterns of the top ligand-receptor (LR) pairs constrained to the tS2 or myeloid cell domain in PD-L1 high lung cancer tissue (*spa18ca02*)

The aggregated CCs of the top three LR pairs ($J_{comp} > 0.2$) with high ligand gene expression and J_{comp} values, were extracted. Then, the intersecting regions between the two aggregated CCs of each LR pair and CCs calculated from either **(a)** tumor cells (tS2) or **(b)** myeloid cells were extracted. The location of modified CCs was mapped to the tissue and J_{comp} values were computed between the two modified CCs.

a

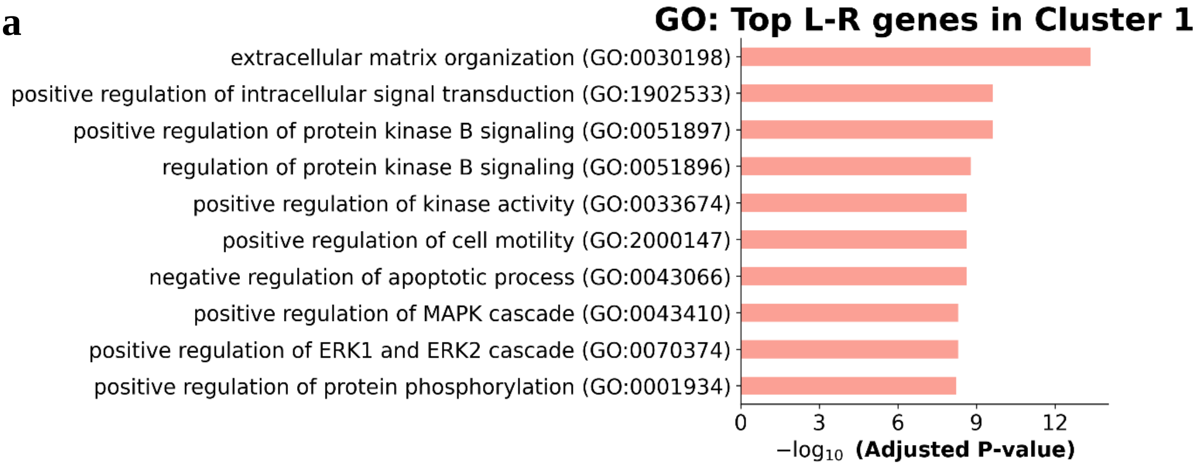

b

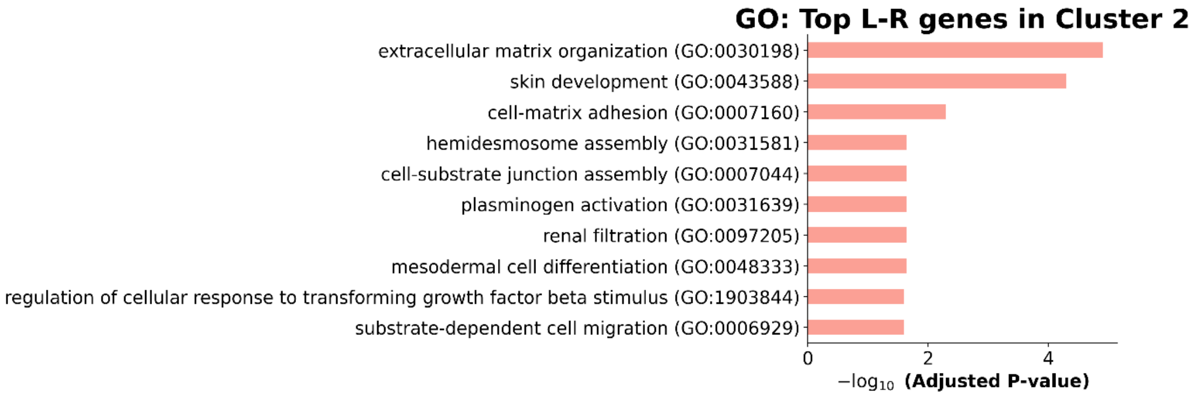

Supplementary Fig. 13. The common top ligand-receptor (LR) interactions in Clusters 1 and 2 defined by infiltration patterns of immune and stromal cells to tumor cells.

Please refer to Fig. 4a for definition of Clusters 1 and 2.

(a) Gene Ontology (GO) analysis was conducted for the top common colocalized LR pairs in Cluster 1 based on a J_{comp} threshold of 0.2. The enriched biological process terms are listed in ascending order of adjusted p-values.

(b) Gene Ontology (GO) analysis was conducted for the top common colocalized LR pairs in Cluster 2 based on a J_{comp} threshold of 0.2. The enriched biological process terms are listed in ascending order of adjusted p-values.

(a)

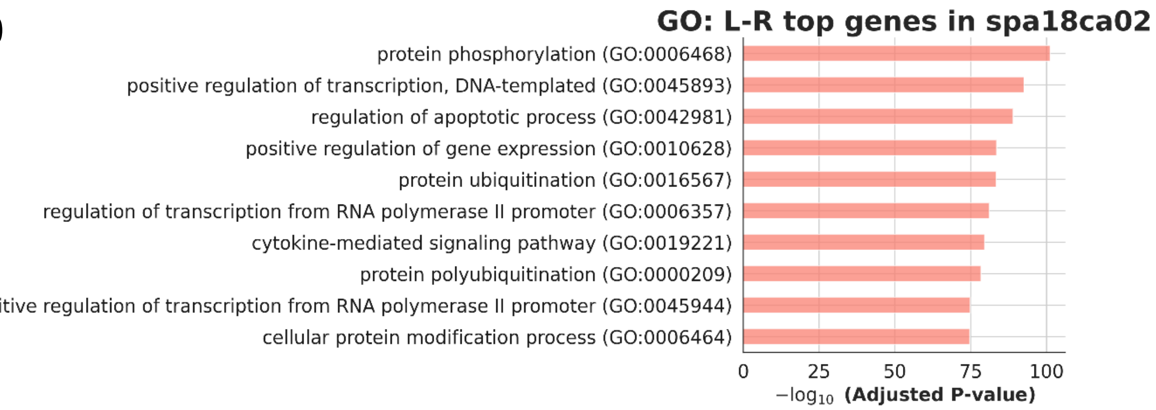

(b)

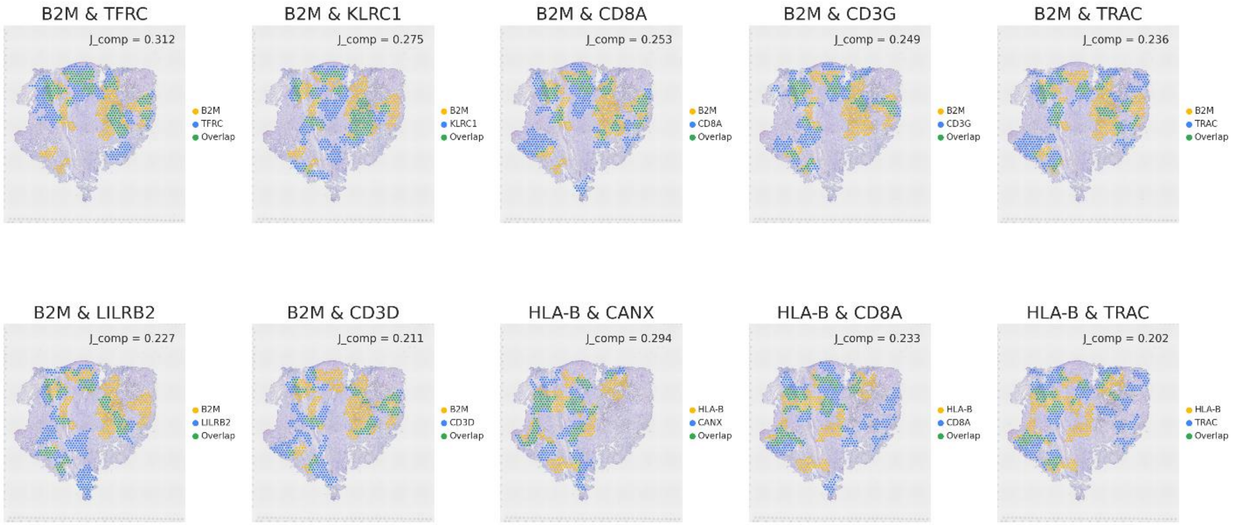

Supplementary Fig. 14. The top ligand-receptor (LR) interactions estimated by STopover using the Omnipath database.

(a) Gene Ontology (GO) analysis was conducted for the top colocalized LR pairs selected based on a J_{comp} threshold of 0.2. The enriched biological process terms are listed in ascending order of adjusted p-values.

(b) The locations of connected components (CCs) for the top 10 LR pairs showing the highest average ligand gene expression in the tissue and the highest J_{comp} value were mapped onto the tissue. CC locations for features x and y are colored yellow and blue, respectively, while intersection locations are shown in green.

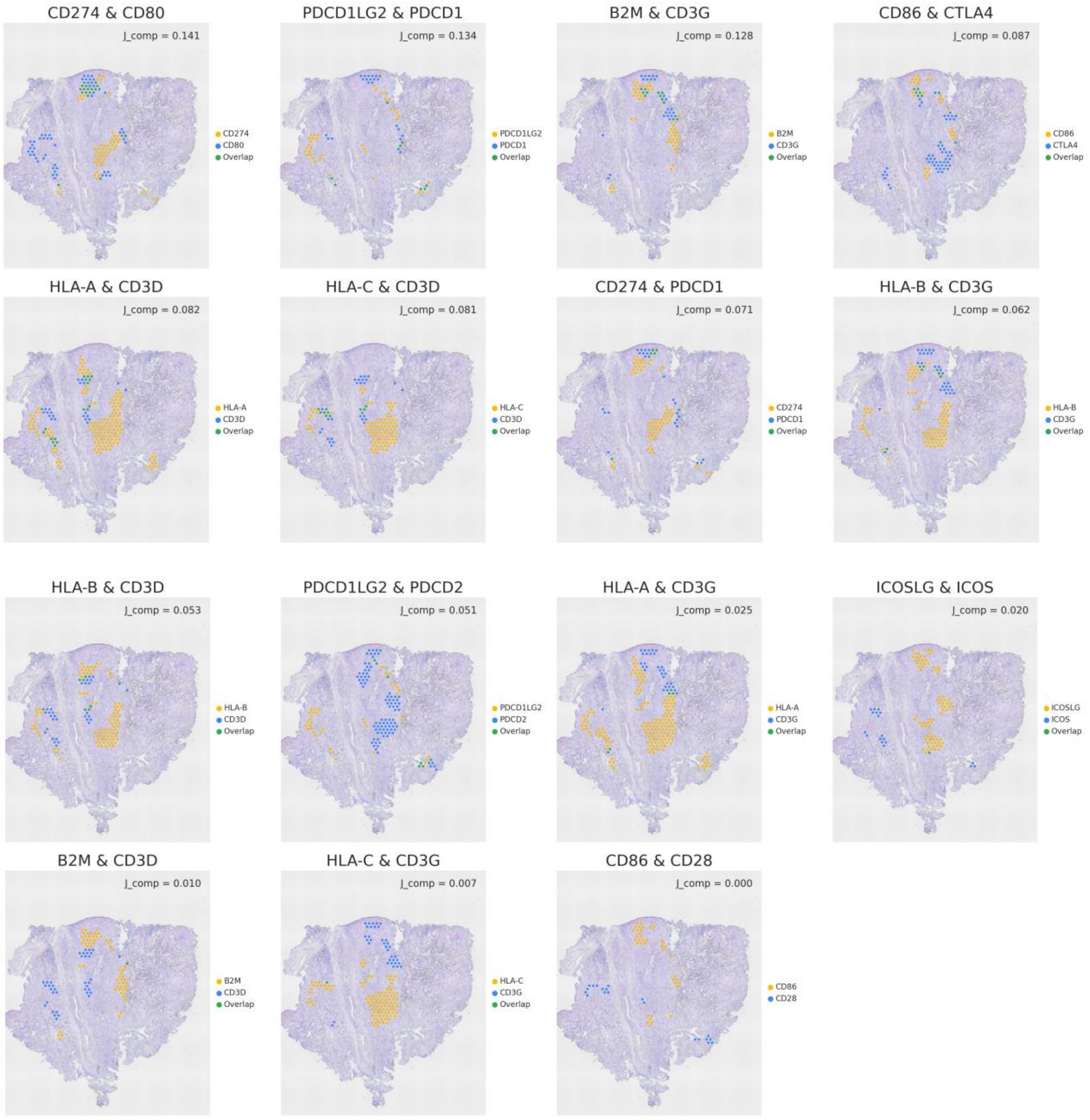
CD274 & CD80
J_comp = 0.141
CD274
CD80
Overlap
PDCD1LG2 & PDCD1
J_comp = 0.134
PDCD1LG2
PDCD1
Overlap
B2M & CD3G
J_comp = 0.128
B2M
CD3G
Overlap
CD86 & CTLA4
J_comp = 0.087
CD86
CTLA4
Overlap
HLA-A & CD3D
J_comp = 0.082
HLA-A
CD3D
Overlap
HLA-C & CD3D
J_comp = 0.081
HLA-C
CD3D
Overlap
CD274 & PDCD1
J_comp = 0.071
CD274
PDCD1
Overlap
HLA-B & CD3G
J_comp = 0.062
HLA-B
CD3G
Overlap
HLA-B & CD3D
J_comp = 0.053
HLA-B
CD3D
Overlap
PDCD1LG2 & PDCD2
J_comp = 0.051
PDCD1LG2
PDCD2
Overlap
HLA-A & CD3G
J_comp = 0.025
HLA-A
CD3G
Overlap
ICOSLG & ICOS
J_comp = 0.020
ICOSLG
ICOS
Overlap
B2M & CD3D
J_comp = 0.010
B2M
CD3D
Overlap
HLA-C & CD3G
J_comp = 0.007
HLA-C
CD3G
Overlap
CD86 & CD28
J_comp = 0.000
CD86
CD28

Supplementary Fig. 15. The spatial overlap patterns of the T cell action-related LR pairs constrained to the tS2 and NK/T cell colocalized domain in PD-L1 high lung cancer tissue (*spa18ca02*)

The aggregated CCs were extracted for the T cell action-related LR pairs. Then, the intersecting regions between the two aggregated CCs of each LR pair and the colocalized domain of tS2 and NK/T cells were extracted. The location of the modified CCs was mapped to the tissue, and J_{comp} values were computed between the two modified CCs.

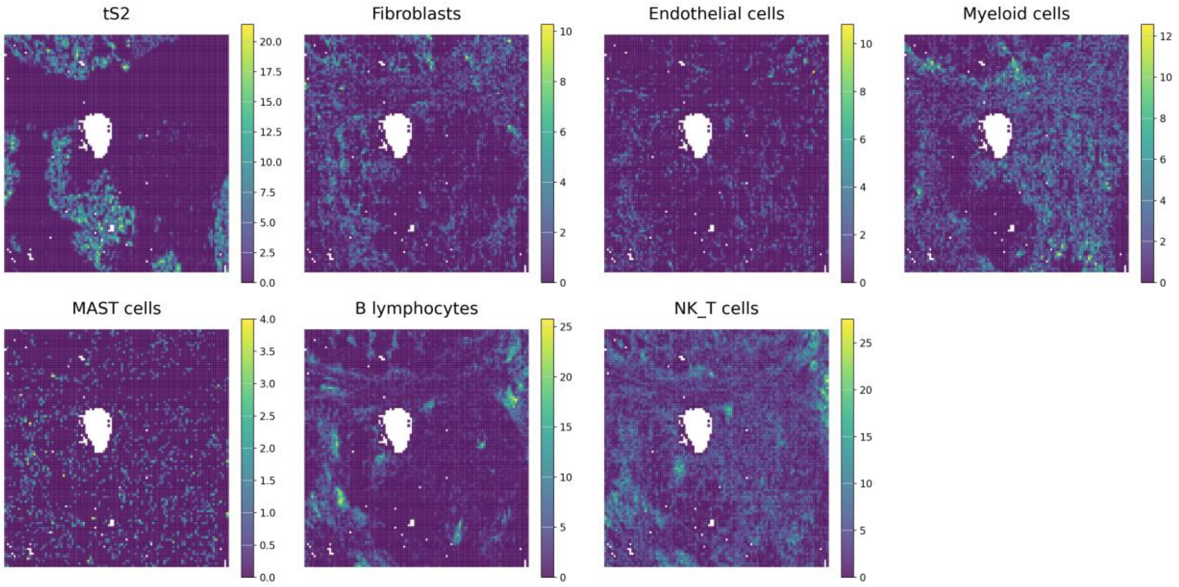

Supplementary Fig. 16. Spatial distribution of tS2 and other main cell types composing the lung cancer tissue in image-based SRT.

The spatial map showing the abundance of tumor cells (tS2) and other main cell types (fibroblasts, endothelial cells, myeloid cells, MAST cells, B lymphocytes, and NK/T cells) were visualized on the transformed grid.

(a)

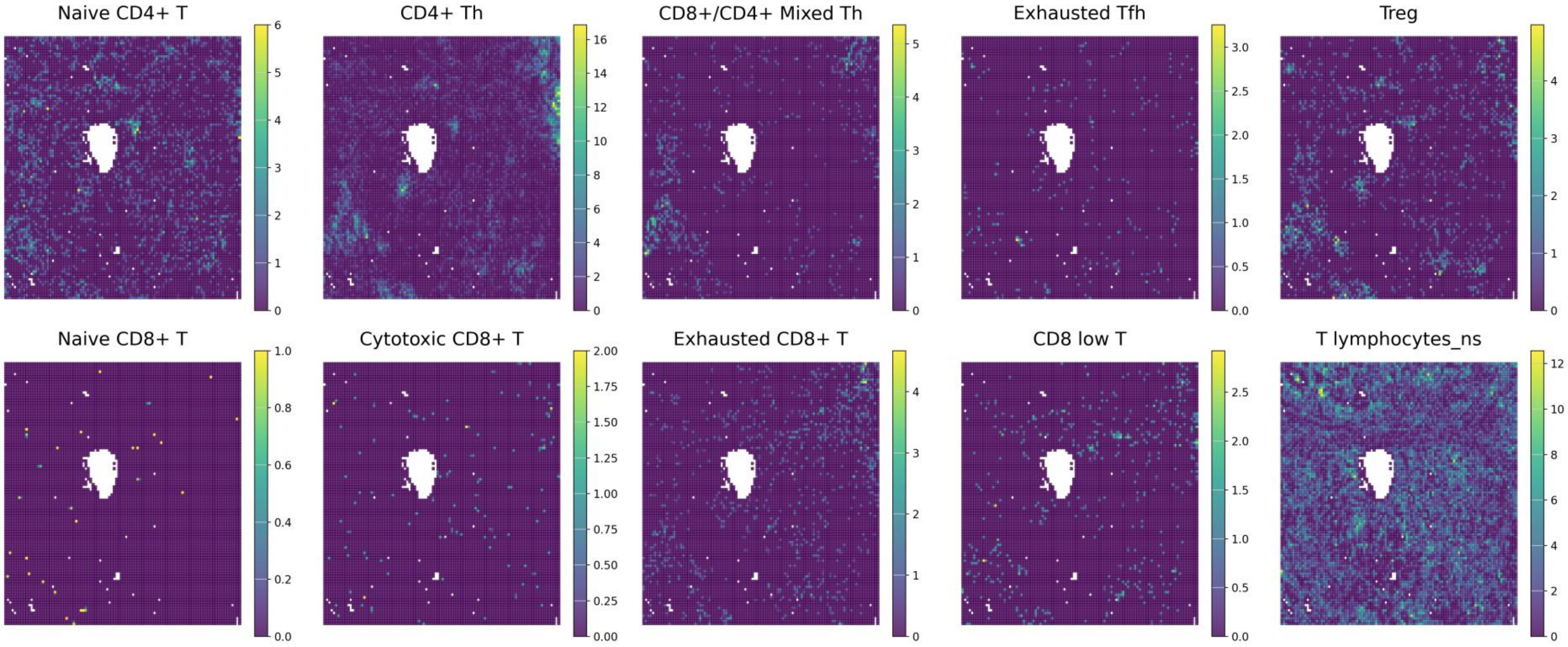
Naive CD4+ T
CD4+ Th
CD8+/CD4+ Mixed Th
Exhausted Tfh
Treg
Naive CD8+ T
Cytotoxic CD8+ T
Exhausted CD8+ T
CD8 low T
T lymphocytes_ns

(b)

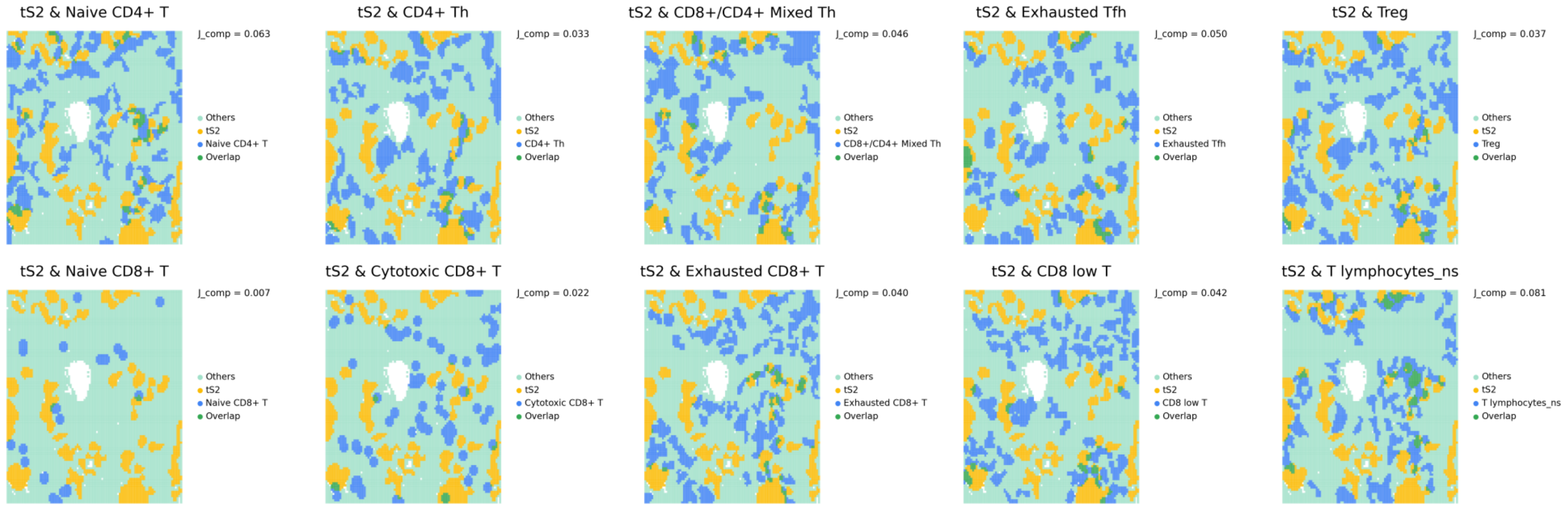

tS2 & Naive CD4+ T
J_comp = 0.063
Others
tS2
Naive CD4+ T
Overlap
tS2 & CD4+ Th
J_comp = 0.033
Others
tS2
CD4+ Th
Overlap
tS2 & CD8+/CD4+ Mixed Th
J_comp = 0.046
Others
tS2
CD8+/CD4+ Mixed Th
Overlap
tS2 & Exhausted Tfh
J_comp = 0.050
Others
tS2
Exhausted Tfh
Overlap
tS2 & Treg
J_comp = 0.037
Others
tS2
Treg
Overlap
tS2 & Naive CD8+ T
J_comp = 0.007
Others
tS2
Naive CD8+ T
Overlap
tS2 & Cytotoxic CD8+ T
J_comp = 0.022
Others
tS2
Cytotoxic CD8+ T
Overlap
tS2 & Exhausted CD8+ T
J_comp = 0.040
Others
tS2
Exhausted CD8+ T
Overlap
tS2 & CD8 low T
J_comp = 0.042
Others
tS2
CD8 low T
Overlap
tS2 & T lymphocytes_ns
J_comp = 0.081
Others
tS2
T lymphocytes_ns
Overlap

Supplementary Fig. 17. The colocalization patterns between tS2 and multiple T cell subtypes in image-based SRT of lung cancer

(a) The spatial map showing the abundance of tumor cells (tS2) and multiple T cell subtypes were visualized on the transformed grid.

(b) The key locations of tS2 and multiple T cells subtypes were represented by CCs, and the overlapping tissue domains were highlighted as the intersecting subregions between the two aggregated CCs.

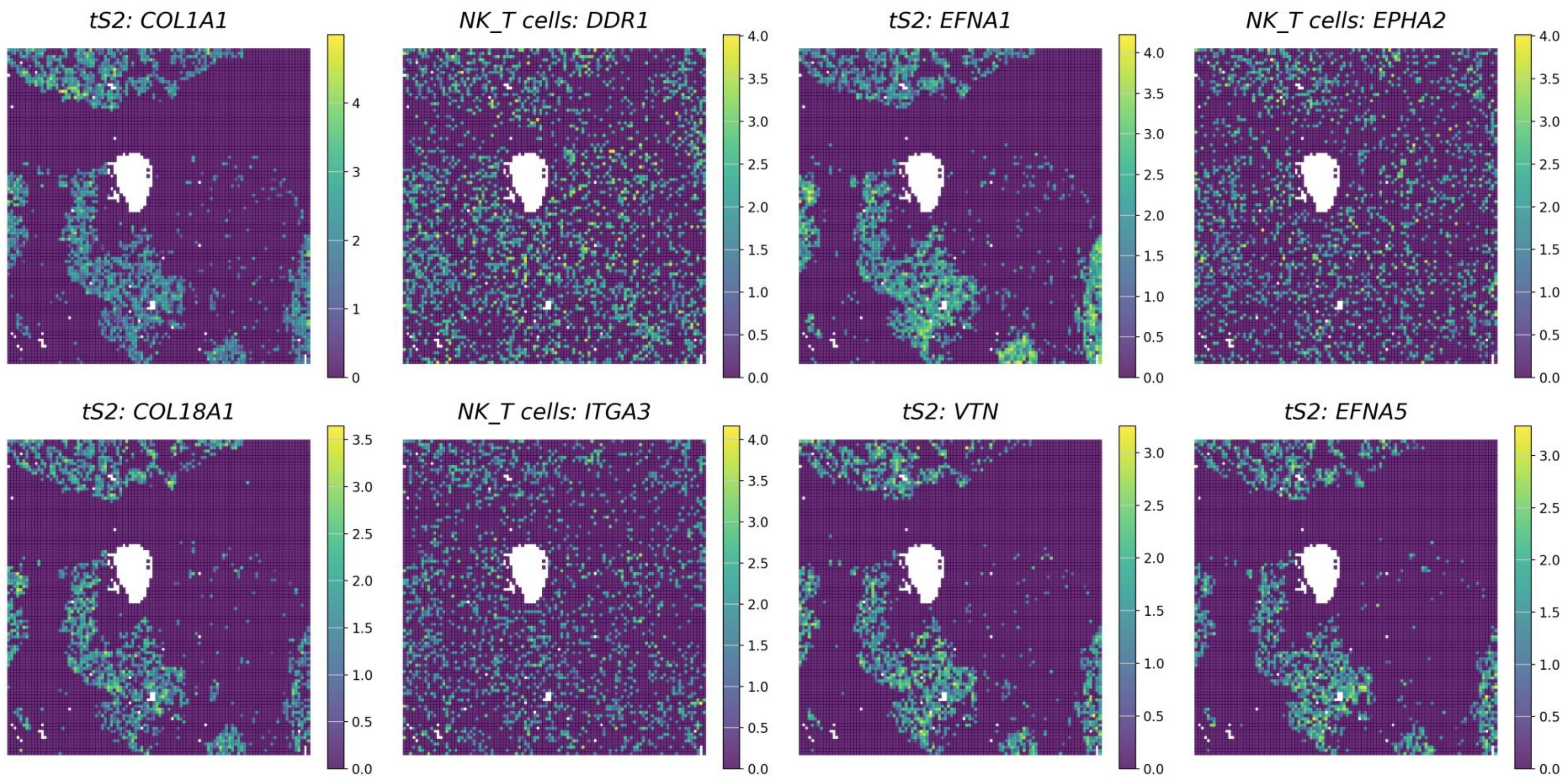

Supplementary Fig. 18. Spatial distribution of the top ligand-receptor expression specific to tS2 and NK/T cells

The spatial map displaying the log-normalized expression of the top ligand and receptor pairs specific to tumor cells (tS2) and NK/T cells, respectively, was visualized on the transformed grid.

a

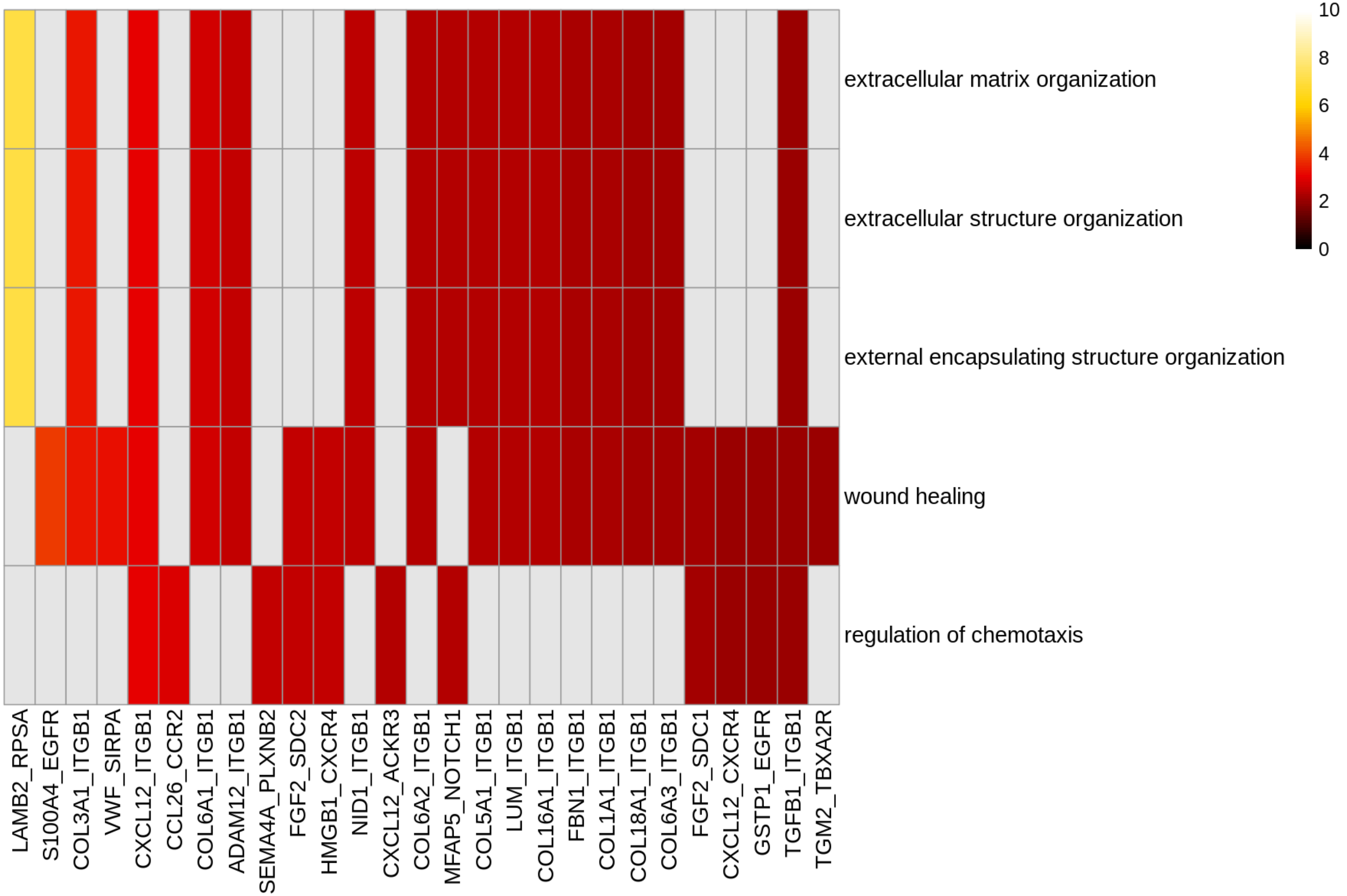

extracellular matrix organization
extracellular structure organization
external encapsulating structure organization
wound healing
regulation of chemotaxis
LAMB2_RPSA
S100A4_EGFR
COL3A1_ITGB1
VWF_SIRPA
CXCL12_ITGB1
CCL26_CCR2
COL6A1_ITGB1
ADAM12_ITGB1
SEMA4A_PLXNB2
FGF2_SDC2
HMGB1_CXCR4
NID1_ITGB1
CXCL12_ACKR3
COL6A2_ITGB1
MFAP5_NOTCH1
COL5A1_ITGB1
LUM_ITGB1
COL16A1_ITGB1
FBN1_ITGB1
COL1A1_ITGB1
COL18A1_ITGB1
COL6A3_ITGB1
FGF2_SDC1
CXCL12_CXCR4
GSTP1_EGFR
TGFB1_ITGB1
TGM2_TBXA2R
10
8
6
4
2
0

b

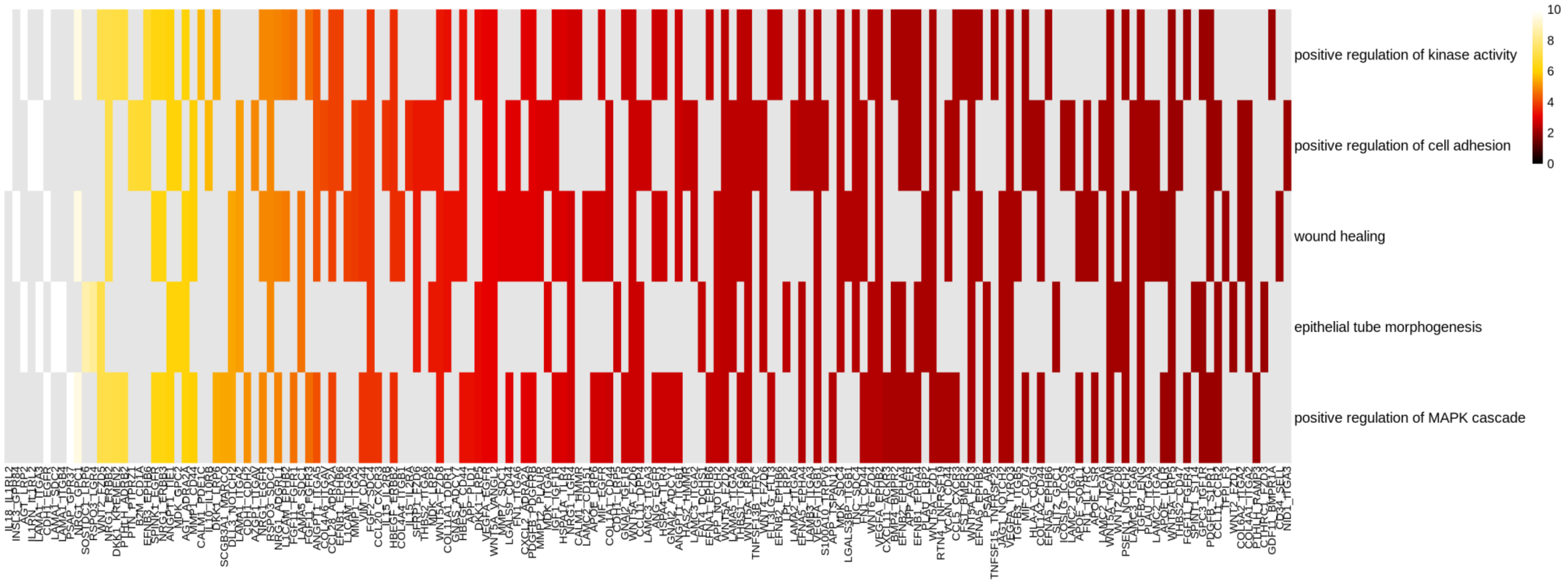
positive regulation of kinase activity
positive regulation of cell adhesion
wound healing
epithelial tube morphogenesis
positive regulation of MAPK cascade
10
8
6
4
2
0

Supplementary Fig. 19. The top differentially upregulated LR pairs and related Gene Ontology (GO) terms in ER+ or TNBC tissues. The spatial LR interaction profiles were estimated by STopover, and differentially upregulated LR interactions in ER+ or TNBC tissues were selected (J_{comp}>0.2 and fold change of J_{comp}>2). The enrichment analysis was performed for the genes in LR pairs and the top 5 enriched GO terms with the lowest adjusted p-values were selected. LR pairs in which either ligand or receptor gene intersected with the genes composing the top GO terms were listed. The upregulated functional terms, the intersecting LR pairs, and the fold change of J_{comp} in **(a)** ER+ and **(b)** TNBC tissues were visualized in heatmaps. The LR pairs were displayed in the descending order of fold change of J_{comp}. In summary, STopover can explain the spatial heterogeneity of TME in breast cancer tissues of different subtypes.

a **The top upregulated LR interaction in ER+ tissues**

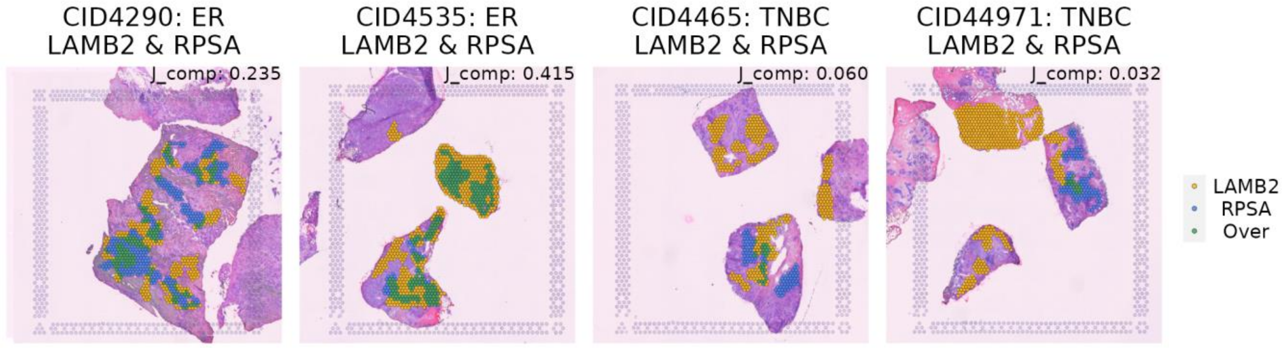

b **The top upregulated LR interaction in TNBC tissues**

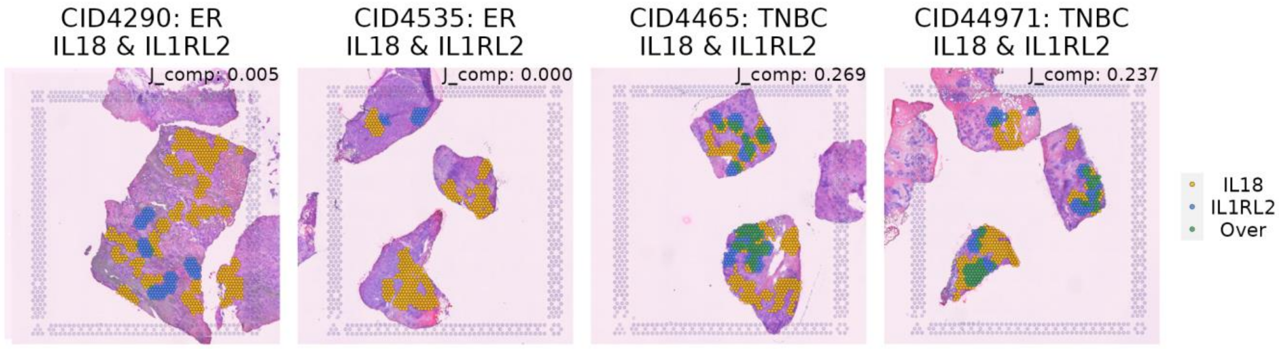

c **Constrained to cancer-associated fibroblasts (CAFs)**

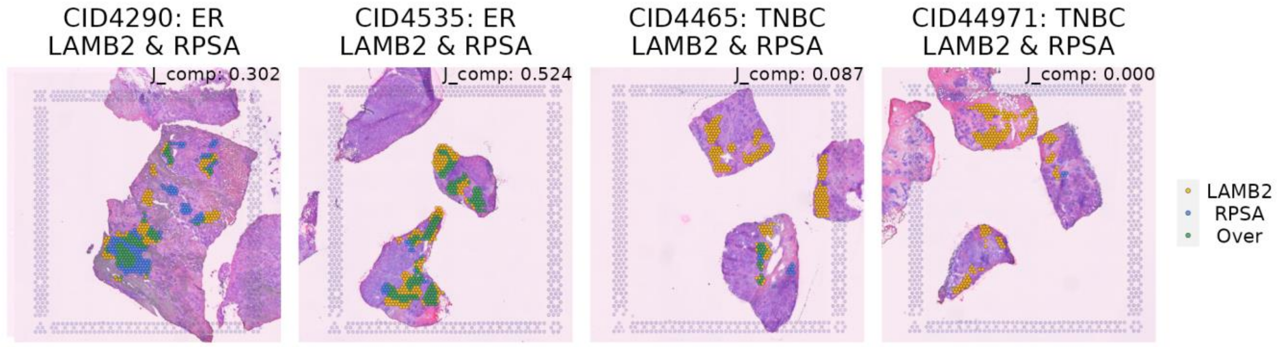

Constrained to cancer epithelial cells

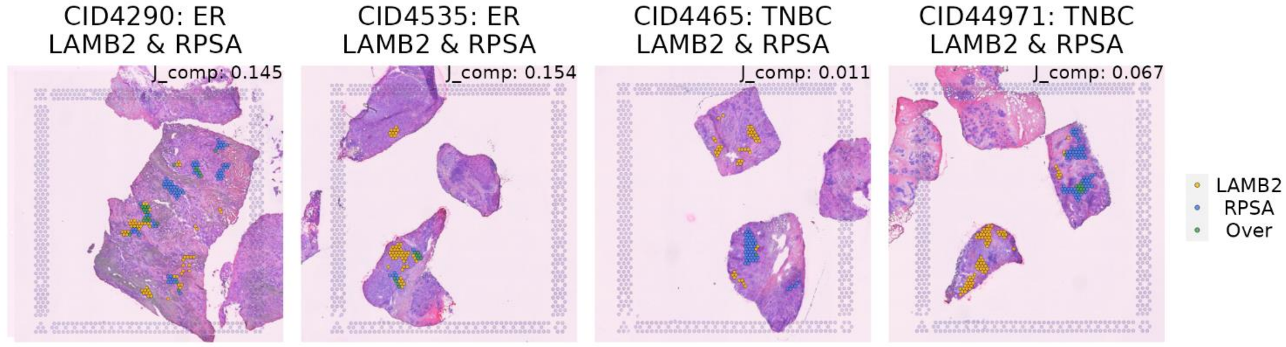

d **Constrained to cancer-associated fibroblasts (CAFs)**

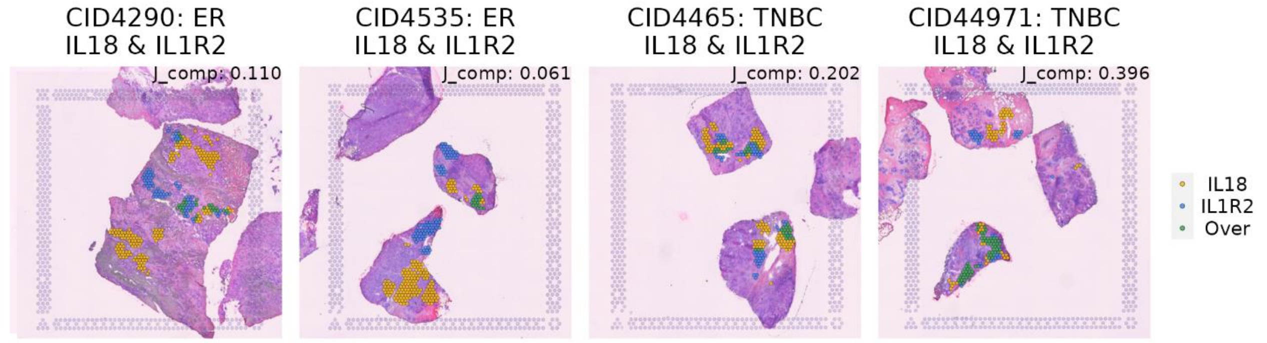

Constrained to cancer epithelial cells

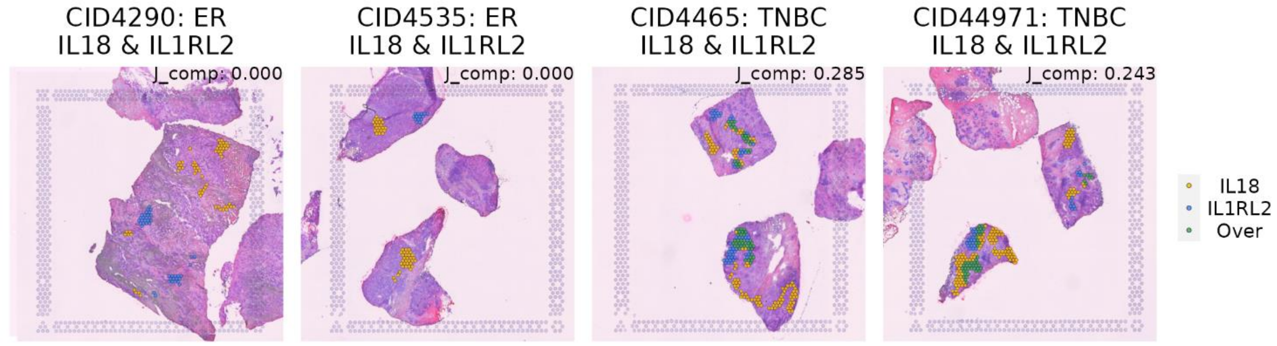

Supplementary Fig. 20. The spatial overlap patterns of the top differential upregulated LR pairs in ER+ or TNBC breast tissues.

The aggregated CCs of the top differentially upregulated LR pairs (J_{comp} >0.2 and average fold change>2) in ER+ and TNBC tissues were extracted. The top LR pair was *LAMB2-RPSA* in ER+ tissues and *IL18-IL1RL2* in TNBC. The key location for (**a**) *LAMB2-RPSA* and (**b**) *IL18-IL1RL2* interactions were represented by CCs, and the overlapping tissue domains were highlighted as the intersecting subregions between the two aggregated CCs. Then, the intersecting regions between the two aggregated CCs of each LR pair and CCs calculated from either cancer-associated fibroblasts (CAFs) or cancer epithelial cells were extracted for (**c**) *LAMB2-RPSA* and (**d**) *IL18-IL1RL2* interactions. The location of modified CCs was mapped to the tissue and J_{comp} values were computed between the two modified CCs.

a

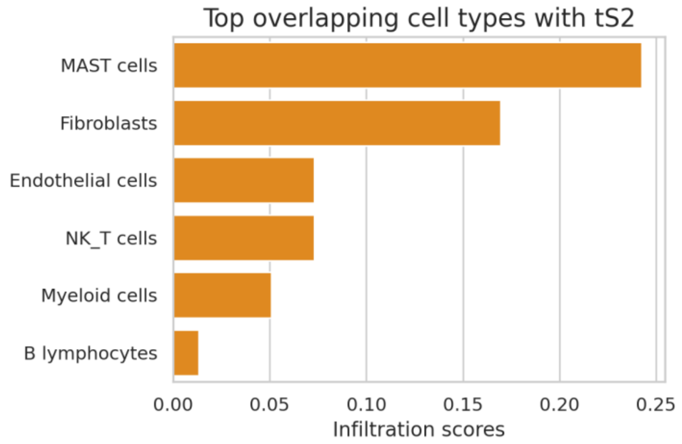
Top overlapping cell types with tS2
MAST cells
Fibroblasts
Endothelial cells
NK_T cells
Myeloid cells
B lymphocytes
0.00
0.05
0.10
0.15
0.20
0.25
Infiltration scores

b

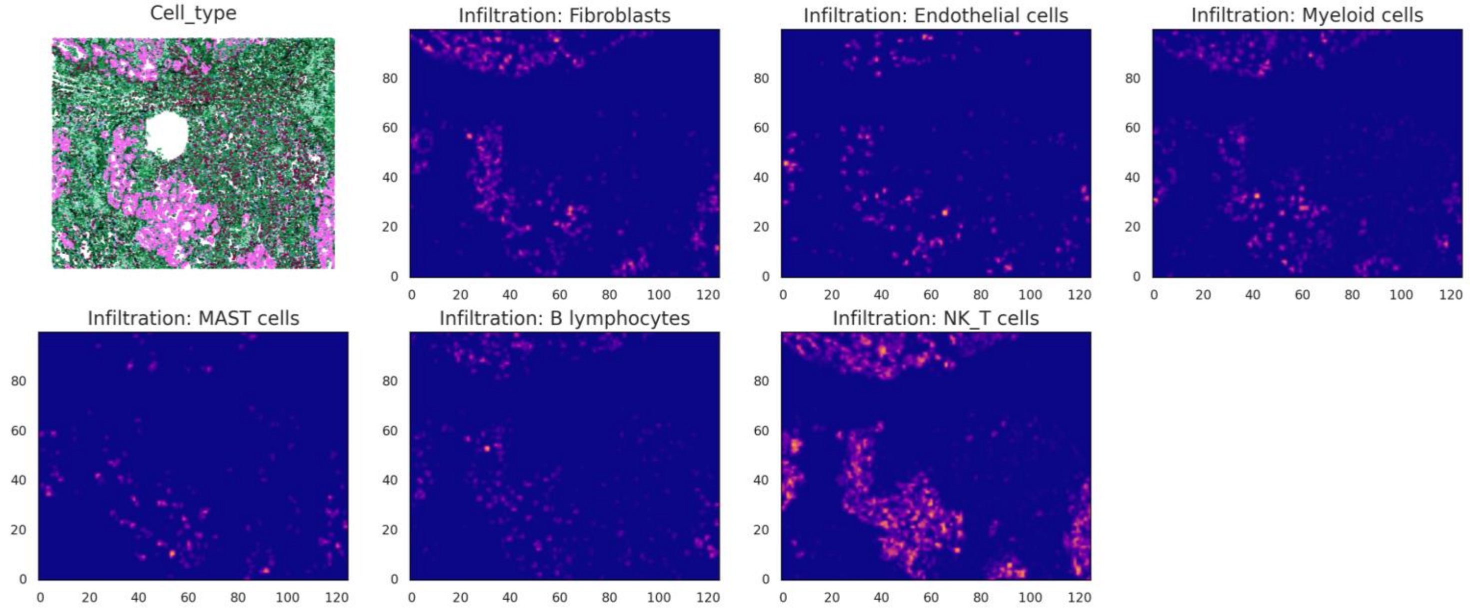
Cell_type
Infiltration: Fibroblasts
Infiltration: Endothelial cells
Infiltration: Myeloid cells
Infiltration: MAST cells
Infiltration: B lymphocytes
Infiltration: NK_T cells

c

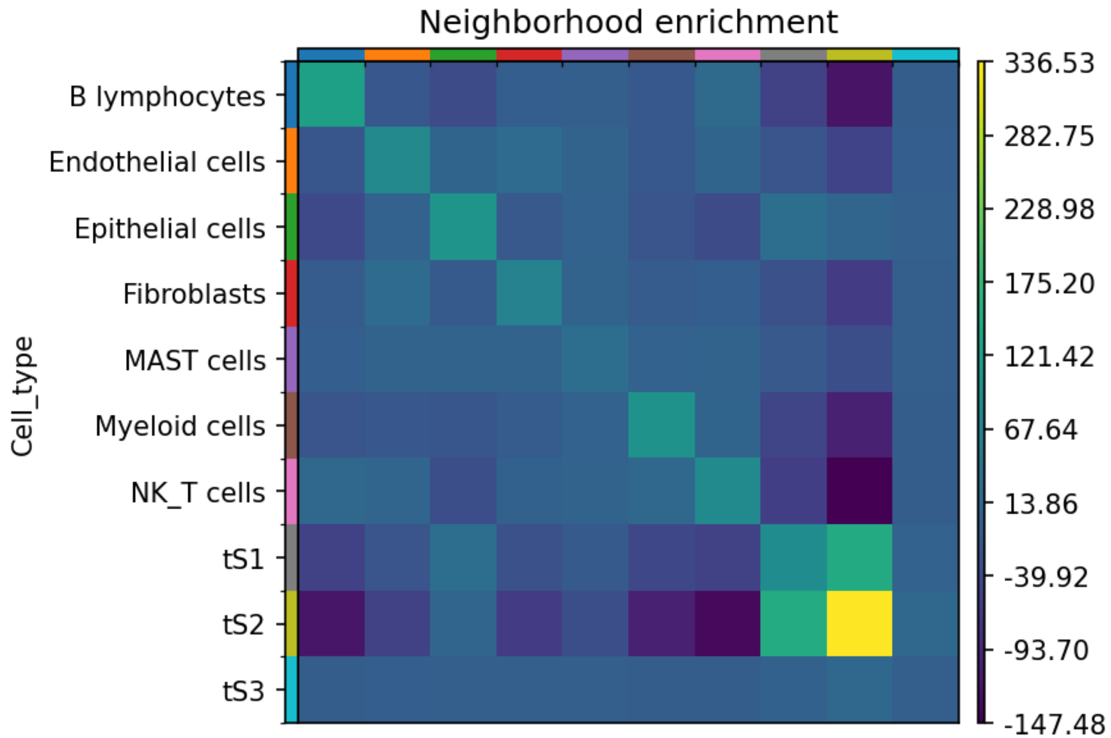

Neighborhood enrichment
Cell_type
B lymphocytes
Endothelial cells
Epithelial cells
Fibroblasts
MAST cells
Myeloid cells
NK_T cells
tS1
tS2
tS3
336.53
282.75
228.98
175.20
121.42
67.64
13.86
-39.92
-93.70
-147.48

d

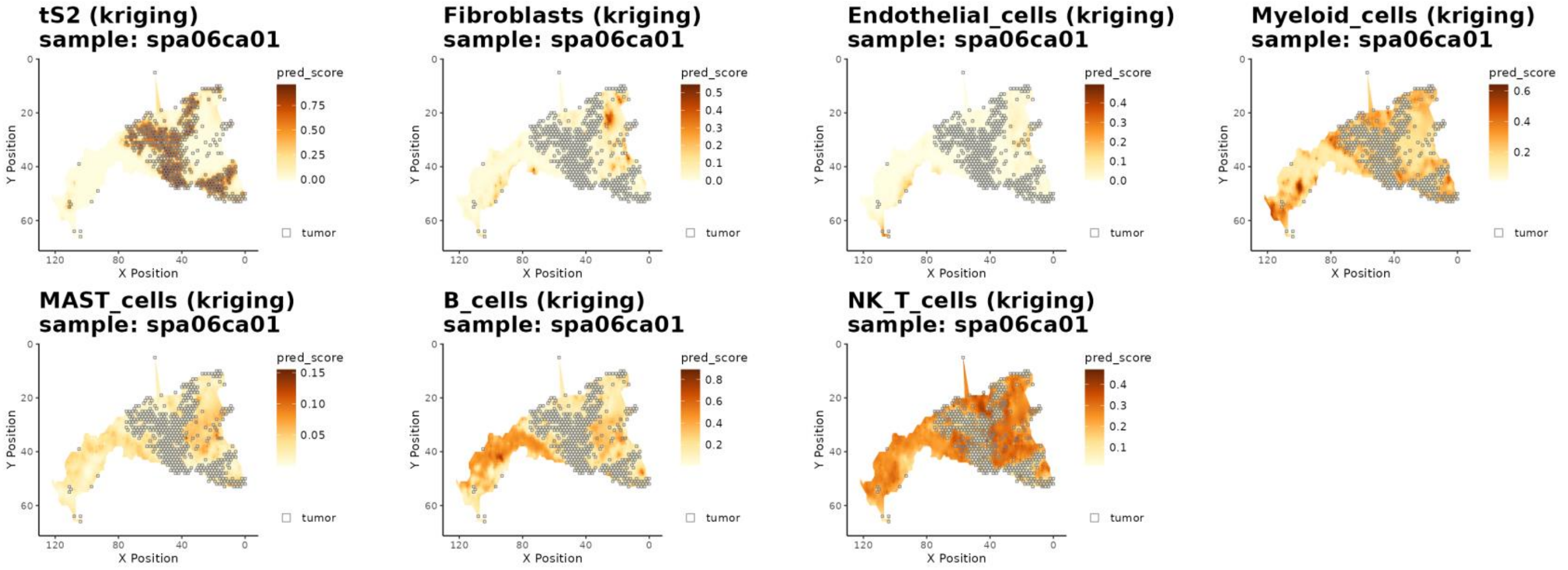
tS2 (kriging)
sample: spa06ca01
Fibroblasts (kriging)
sample: spa06ca01
Endothelial_cells (kriging)
sample: spa06ca01
Myeloid_cells (kriging)
sample: spa06ca01
MAST_cells (kriging)
sample: spa06ca01
B_cells (kriging)
sample: spa06ca01
NK_T_cells (kriging)
sample: spa06ca01
pred_score
tumor
X Position
Y Position

e

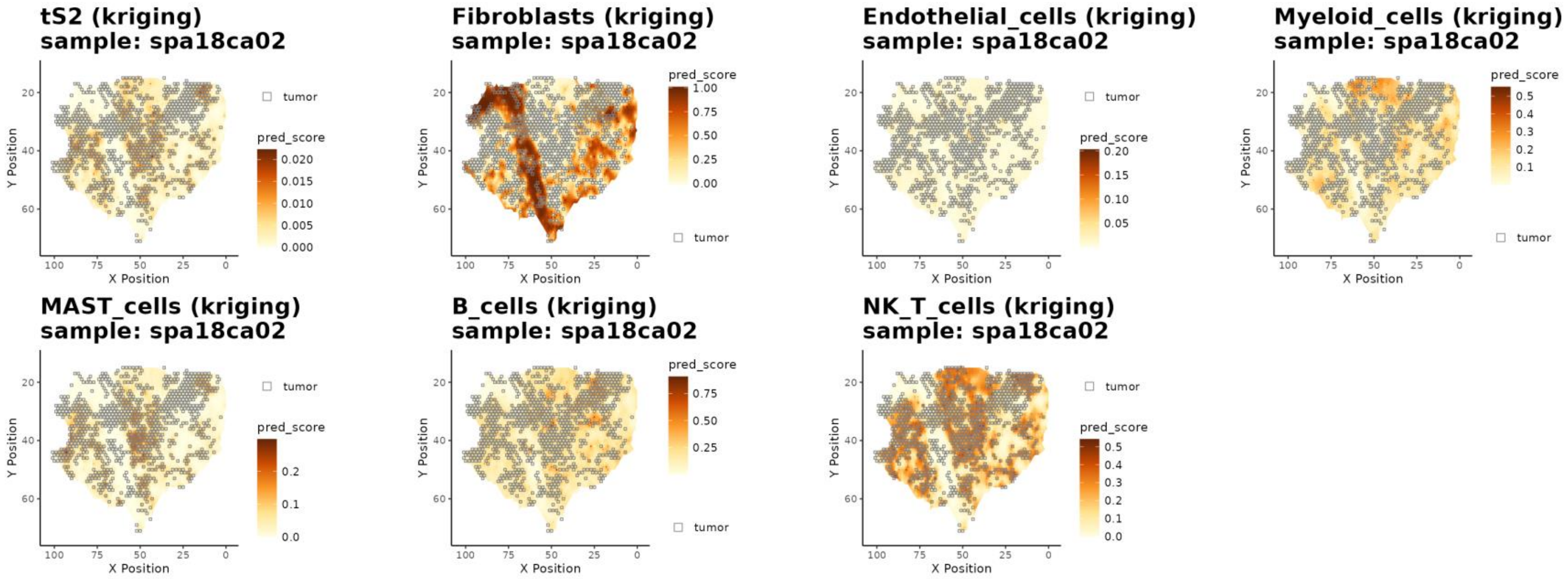
tS2 (kriging)
sample: spa18ca02
Fibroblasts (kriging)
sample: spa18ca02
Endothelial_cells (kriging)
sample: spa18ca02
Myeloid_cells (kriging)
sample: spa18ca02
MAST_cells (kriging)
sample: spa18ca02
B_cells (kriging)
sample: spa18ca02
NK_T_cells (kriging)
sample: spa18ca02
pred_score
tumor
X Position
Y Position

Supplementary Fig. 21. Investigation of spatial heterogeneity of the lung cancer using ATHENA and spatialGE.

First, ATHENA was applied to the image-based SRT of the lung cancer dataset and infiltration scores of six cells types (fibroblasts, endothelial cells, myeloid cells, MAST cells, B lymphocytes, and NK/T cells) to the tumor cells (tS2) were calculated. (**a**) The global infiltration score represents the overall infiltration of a certain cell type into the cancer cell in a given tissue section, and it was visualized as a barplot in the left panel. (**b**) The local infiltration score represents the local extent of infiltration of a certain cell type into the cancer cell and it was visualized on a grid with a unit size (step_size parameter) of 220.

Second, Squidpy was applied to the image-based SRT of lung cancer dataset and the infiltration score of six cell types to the tumor cells was calculated. **(c)** The heatmap shows the neighborhood enrichment (Z-score) of colocalization between all pairs of cell types present in the lung cancer tissue.

Third, spatialGE was applied to barcode-based SRT of lung cancer dataset and the spots were classified into either tumor or stromal spots. The spatial cell fraction predicted by CellDART was adopted, and high-resolution spatial maps of all cell types were generated. Based on the tumor classification results and high-resolution spatial map, the immune phenotypes of the (**d**) PD-L1 low (*spa06ca01*) and (**e**) PD-L1 high (*spa18ca02*) cancer tissues can be qualitatively assessed.

(a) **Epithelial cells vs. NK/T cells in PD-L1 low tissue (*spa06ca01*)**

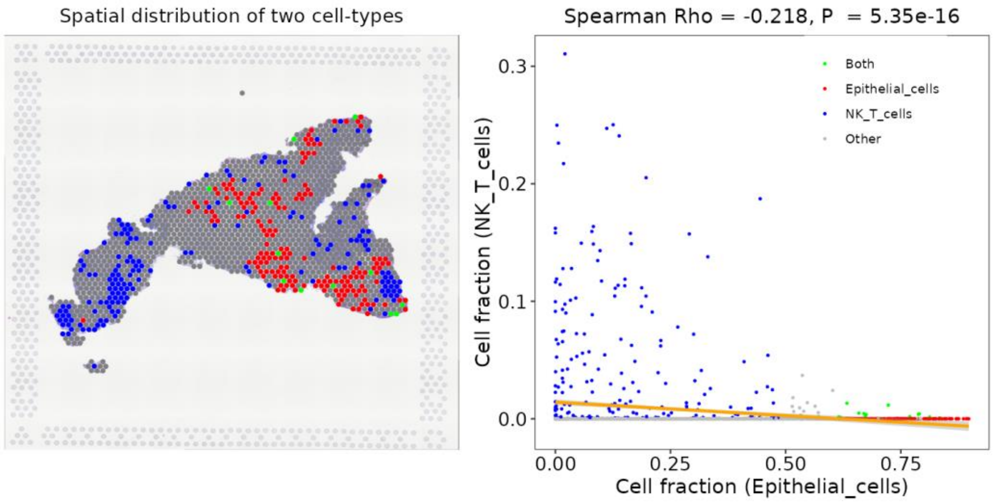

Epithelial cells vs. mast cells in PD-L1 low tissue (*spa06ca01*)

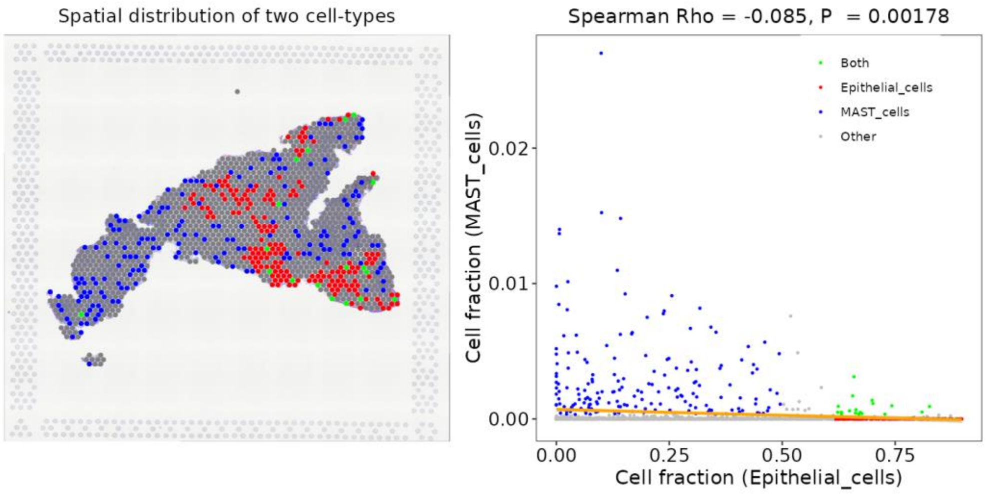

(b) **Epithelial cells vs. mast cells in PD-L1 high tissue (*spa18ca02*)**

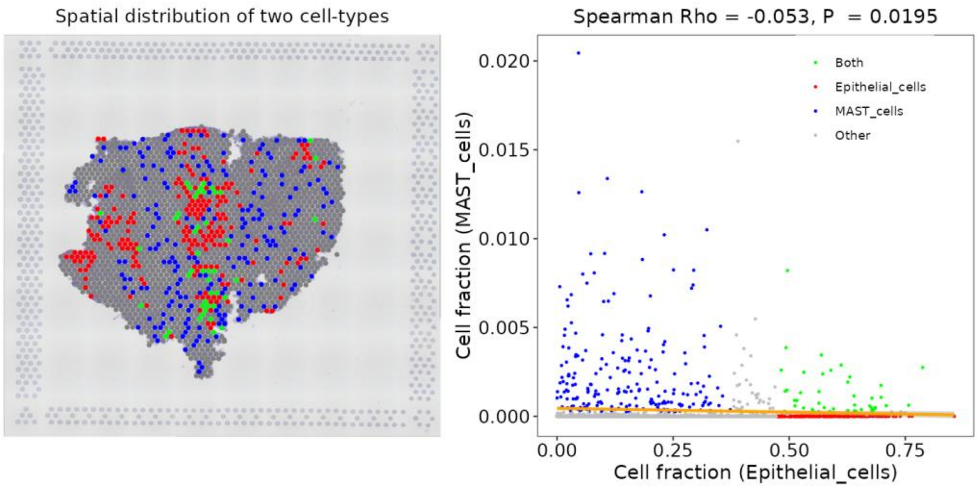

(c)

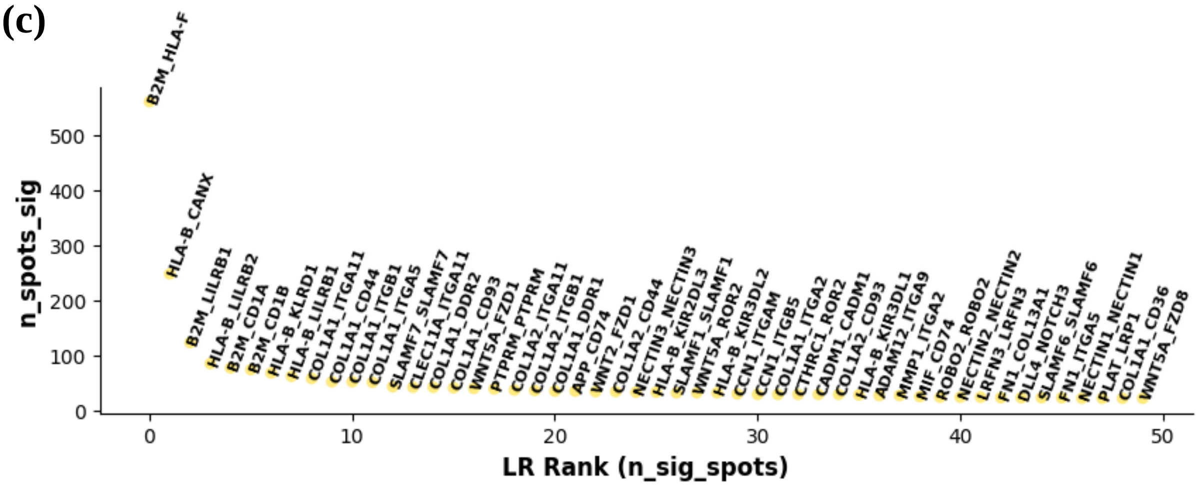

n_spots_sig
0
100
200
300
400
500
LR Rank (n_sig_spots)
0
10
20
30
40
50
B2M_HLA-F
HLA-B_CANX
B2M_LILRB1
HLA-B_LILRB2
B2M_CD1A
B2M_CD1B
HLA-B_KLRD1
HLA-B_LILRB1
COL1A1_ITGA11
COL1A1_CD44
COL1A1_ITGB1
COL1A1_ITGA5
SLAMF7_SLAMF7
CLEC11A_ITGA11
COL1A1_DDR2
COL1A1_CD93
WNT5A_FZD1
PTPRM_PTPRM
COL1A2_ITGA11
COL1A2_ITGB1
COL1A1_DDR1
APP_CD74
WNT2_FZD1
COL1A2_CD44
NECTIN3_NECTIN3
HLA-B_KIR2DL3
SLAMF1_SLAMF1
WNT5A_ROR2
HLA-B_KIR3DL2
CCN1_ITGAM
CCN1_ITGB5
COL1A1_ITGA2
CTHRC1_ROR2
CADM1_CADM1
COL1A2_CD93
HLA-B_KIR3DL1
ADAM12_ITGA9
MMP1_ITGA2
MIF_CD74
ROBO2_ROBO2
NECTIN2_NECTIN2
LRFN3_LRFN3
FN1_COL13A1
DLL4_NOTCH3
SLAMF6_SLAMF6
FN1_ITGA5
NECTIN1_NECTIN1
PLAT_LRP1
COL1A1_CD36
WNT5A_FZD8

(d)

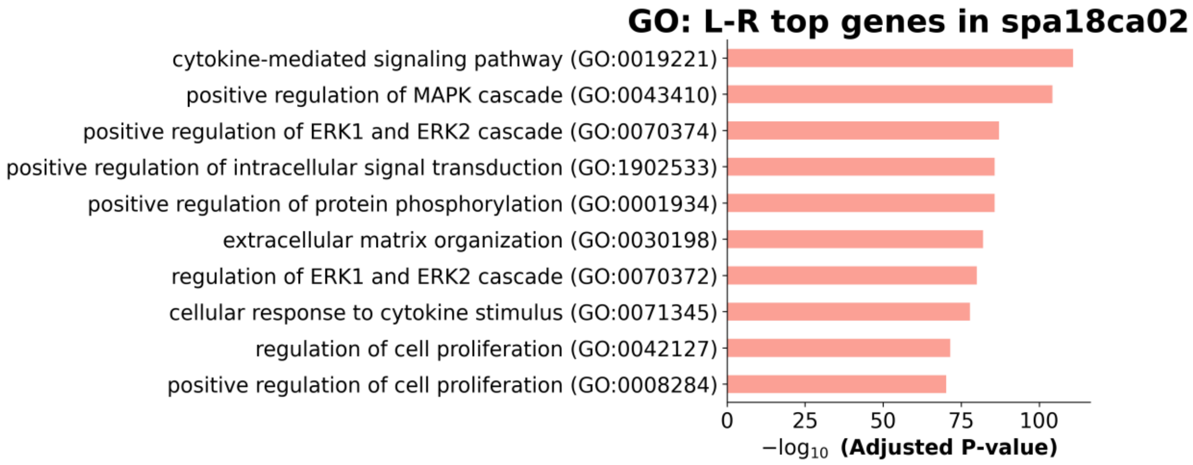

GO: L-R top genes in spa18ca02
cytokine-mediated signaling pathway (GO:0019221)
positive regulation of MAPK cascade (GO:0043410)
positive regulation of ERK1 and ERK2 cascade (GO:0070374)
positive regulation of intracellular signal transduction (GO:1902533)
positive regulation of protein phosphorylation (GO:0001934)
extracellular matrix organization (GO:0030198)
regulation of ERK1 and ERK2 cascade (GO:0070372)
cellular response to cytokine stimulus (GO:0071345)
regulation of cell proliferation (GO:0042127)
positive regulation of cell proliferation (GO:0008284)
0
25
50
75
100
$-\log_{10}$ (Adjusted P-value)

(e)

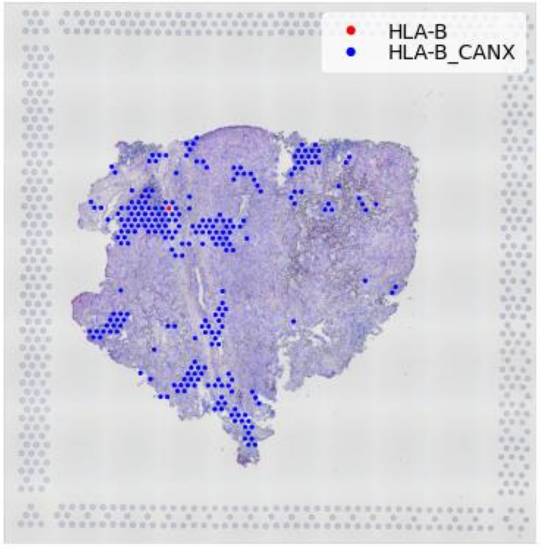

HLA-B
HLA-B_CANX

(f)

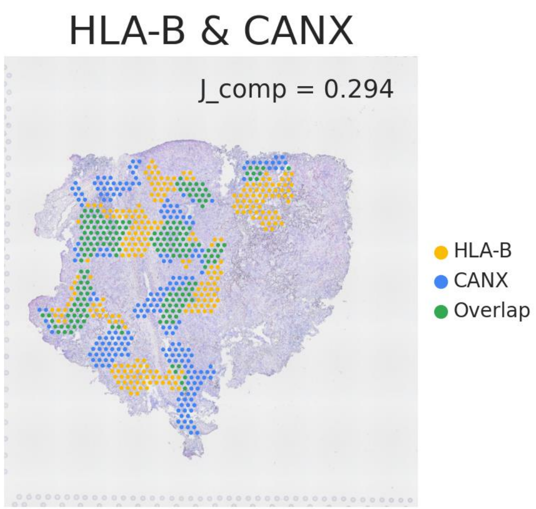

HLA-B & CANX
J_comp = 0.294
HLA-B
CANX
Overlap

Supplementary Fig. 22. Investigation of spatial heterogeneity in lung cancer using SpaCET and stLearn.

First, we applied SpaCET to the barcode-based SRT datasets of lung cancer. We used spatial cell fraction predictions provided by CellDART. Next, we evaluated the cell-cell colocalization patterns of six cell types (fibroblasts, endothelial cells, myeloid cells, MAST cells, B lymphocytes, and NK/T cells) with epithelial cells in both PD-L1 low (spa06ca01) and PD-L1 high (spa18ca02) tissues. **(a)** In the case of PD-L1 low tissue, only NK/T cells and mast cells showed significant interactions, while in **(b)** PD-L1 high tissue, mast cells showed significant interactions. Only the significant cell types are displayed. In the left panel, spatial patterns of epithelial cells (red), cell types of interest (blue), and colocalized regions (green) were visualized on the tissue. In the right panel, scatter plots and Spearman's correlation coefficients between epithelial cells and cell types of interest are displayed.

Second, we applied stLearn to the barcode-based SRT datasets of lung cancer to estimate dominant ligand-receptor (LR) interactions. **(c)** The summary plot illustrates the ranking of LR pairs based on the number of significant spots (x-axis) and the number of significant spots (y-axis). **(d)** Gene Ontology (GO) analysis was conducted for all significant colocalized LR pairs. The enriched biological process terms are listed in ascending order of adjusted p-values. One of the top LR interaction pairs that overlapped with the LR interaction results from STopover (based on Omnipath) was selected: *HLA-B* and *CANX*. Spatial patterns of the ligand, *HLA-B*, and receptor, *CANX*, as well as colocalized regions, were visualized on the tissue using **(e)** stLearn (*HLA-B*: red, *CANX*: green, colocalized: blue) and **(f)** STopover (*HLA-B*: yellow, *CANX*: blue, colocalized: green).

a

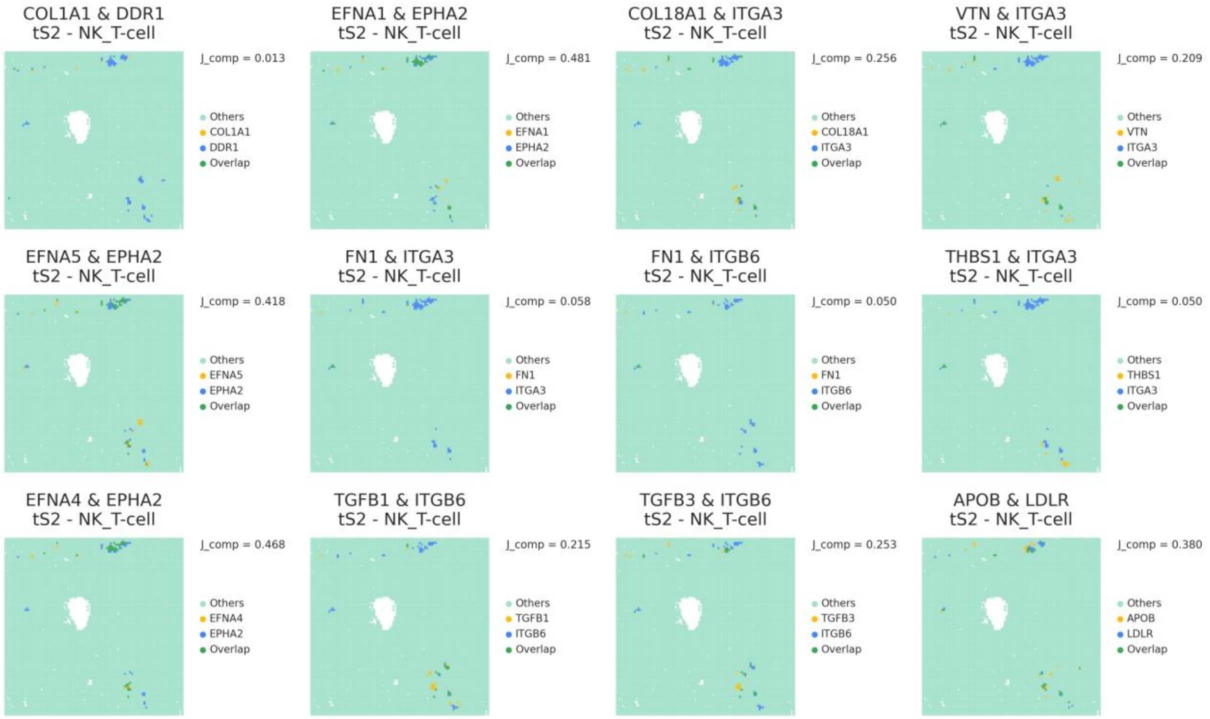
COL1A1 & DDR1
tS2 - NK_T-cell
J_comp = 0.013
Others
COL1A1
DDR1
Overlap
EFNA1 & EPHA2
tS2 - NK_T-cell
J_comp = 0.481
Others
EFNA1
EPHA2
Overlap
COL18A1 & ITGA3
tS2 - NK_T-cell
J_comp = 0.256
Others
COL18A1
ITGA3
Overlap
VTN & ITGA3
tS2 - NK_T-cell
J_comp = 0.209
Others
VTN
ITGA3
Overlap
EFNA5 & EPHA2
tS2 - NK_T-cell
J_comp = 0.418
Others
EFNA5
EPHA2
Overlap
FN1 & ITGA3
tS2 - NK_T-cell
J_comp = 0.058
Others
FN1
ITGA3
Overlap
FN1 & ITGB6
tS2 - NK_T-cell
J_comp = 0.050
Others
FN1
ITGB6
Overlap
THBS1 & ITGA3
tS2 - NK_T-cell
J_comp = 0.050
Others
THBS1
ITGA3
Overlap
EFNA4 & EPHA2
tS2 - NK_T-cell
J_comp = 0.468
Others
EFNA4
EPHA2
Overlap
TGFB1 & ITGB6
tS2 - NK_T-cell
J_comp = 0.215
Others
TGFB1
ITGB6
Overlap
TGFB3 & ITGB6
tS2 - NK_T-cell
J_comp = 0.253
Others
TGFB3
ITGB6
Overlap
APOB & LDLR
tS2 - NK_T-cell
J_comp = 0.380
Others
APOB
LDLR
Overlap

b

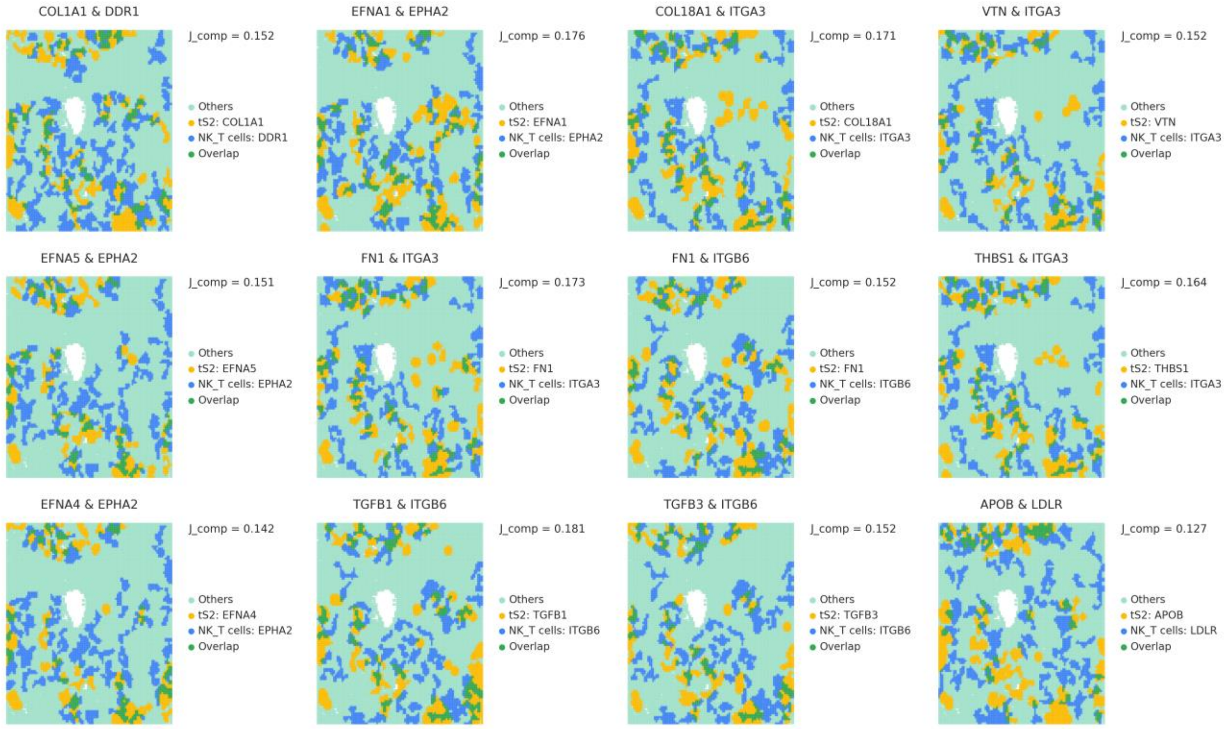
COL1A1 & DDR1
J_comp = 0.152
Others
tS2: COL1A1
NK_T cells: DDR1
Overlap
EFNA1 & EPHA2
J_comp = 0.176
Others
tS2: EFNA1
NK_T cells: EPHA2
Overlap
COL18A1 & ITGA3
J_comp = 0.171
Others
tS2: COL18A1
NK_T cells: ITGA3
Overlap
VTN & ITGA3
J_comp = 0.152
Others
tS2: VTN
NK_T cells: ITGA3
Overlap
EFNA5 & EPHA2
J_comp = 0.151
Others
tS2: EFNA5
NK_T cells: EPHA2
Overlap
FN1 & ITGA3
J_comp = 0.173
Others
tS2: FN1
NK_T cells: ITGA3
Overlap
FN1 & ITGB6
J_comp = 0.152
Others
tS2: FN1
NK_T cells: ITGB6
Overlap
THBS1 & ITGA3
J_comp = 0.164
Others
tS2: THBS1
NK_T cells: ITGA3
Overlap
EFNA4 & EPHA2
J_comp = 0.142
Others
tS2: EFNA4
NK_T cells: EPHA2
Overlap
TGFB1 & ITGB6
J_comp = 0.181
Others
tS2: TGFB1
NK_T cells: ITGB6
Overlap
TGFB3 & ITGB6
J_comp = 0.152
Others
tS2: TGFB3
NK_T cells: ITGB6
Overlap
APOB & LDLR
J_comp = 0.127
Others
tS2: APOB
NK_T cells: LDLR
Overlap

Supplementary Fig. 23. Evaluation of a method for estimating cell type-specific LR interaction in barcode-based SRT.

To assess the feasibility of an alternative approach for estimating cell type-specific LR interaction in barcode-based SRT, an image-based SRT dataset was utilized, as it provides real cell type-specific expression profiles. (**a**) The alternative approach suggested for barcode-based SRT was applied, and the tS2 and NK/T cell-specific LR interaction was predicted. It was predicted by intersecting the regions between the two aggregated CCs of the given LR pairs and colocalized domain of tS2 and NK/T cells. The modified CCs were mapped to the tissue, and the overlapping tissue domain was considered the key niche for tS2 and NK/T cell-specific LR interactions. (**b**) Then, the ligand expression in tS2 and the receptor expression in NK/T cells were used to identify the key niches of LR interactions. The results were considered the reference standard for the tS2 and NK/T cell-specific interaction.

(a)

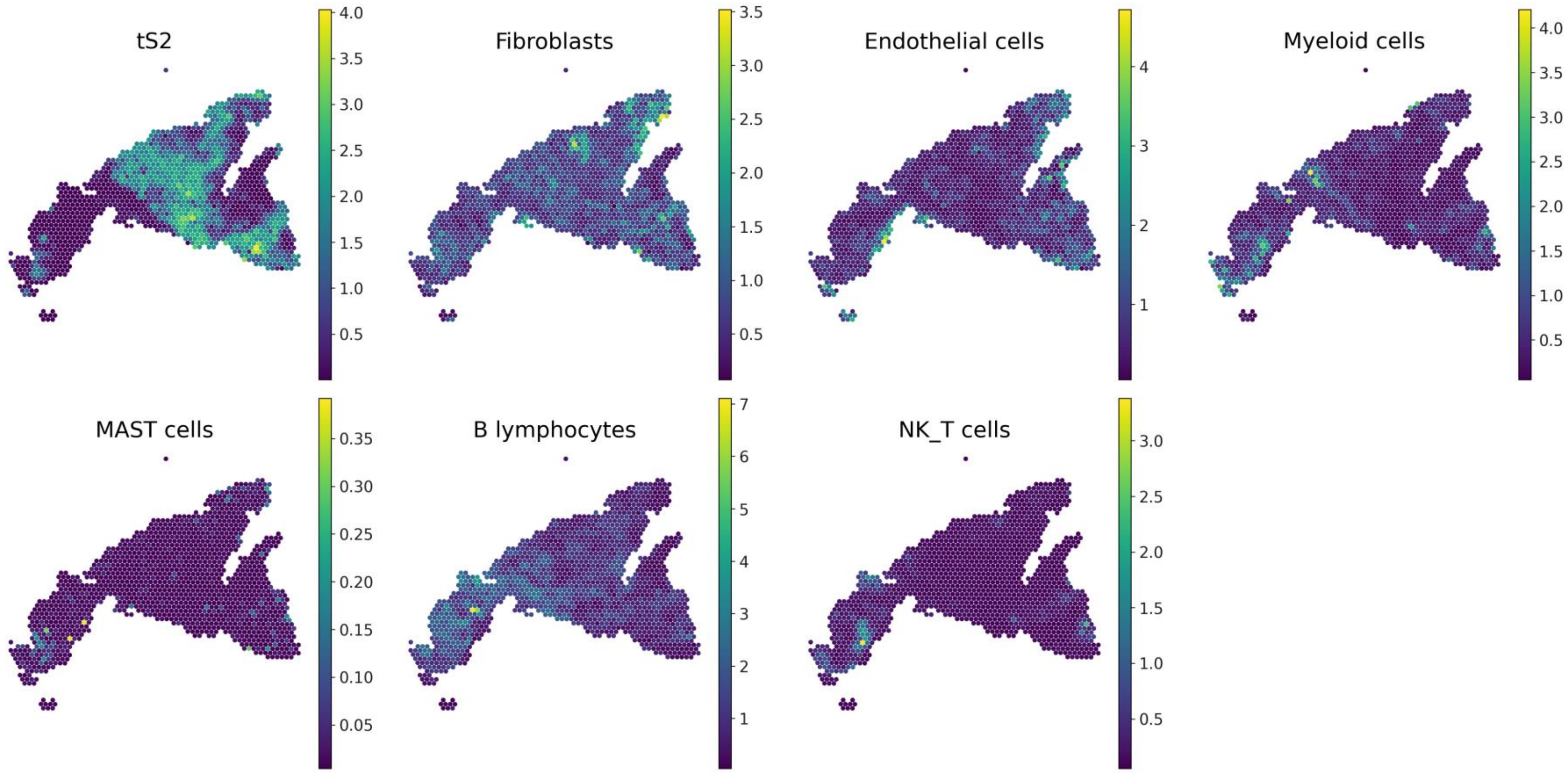
tS2
Fibroblasts
Endothelial cells
Myeloid cells
MAST cells
B lymphocytes
NK_T cells

(b)

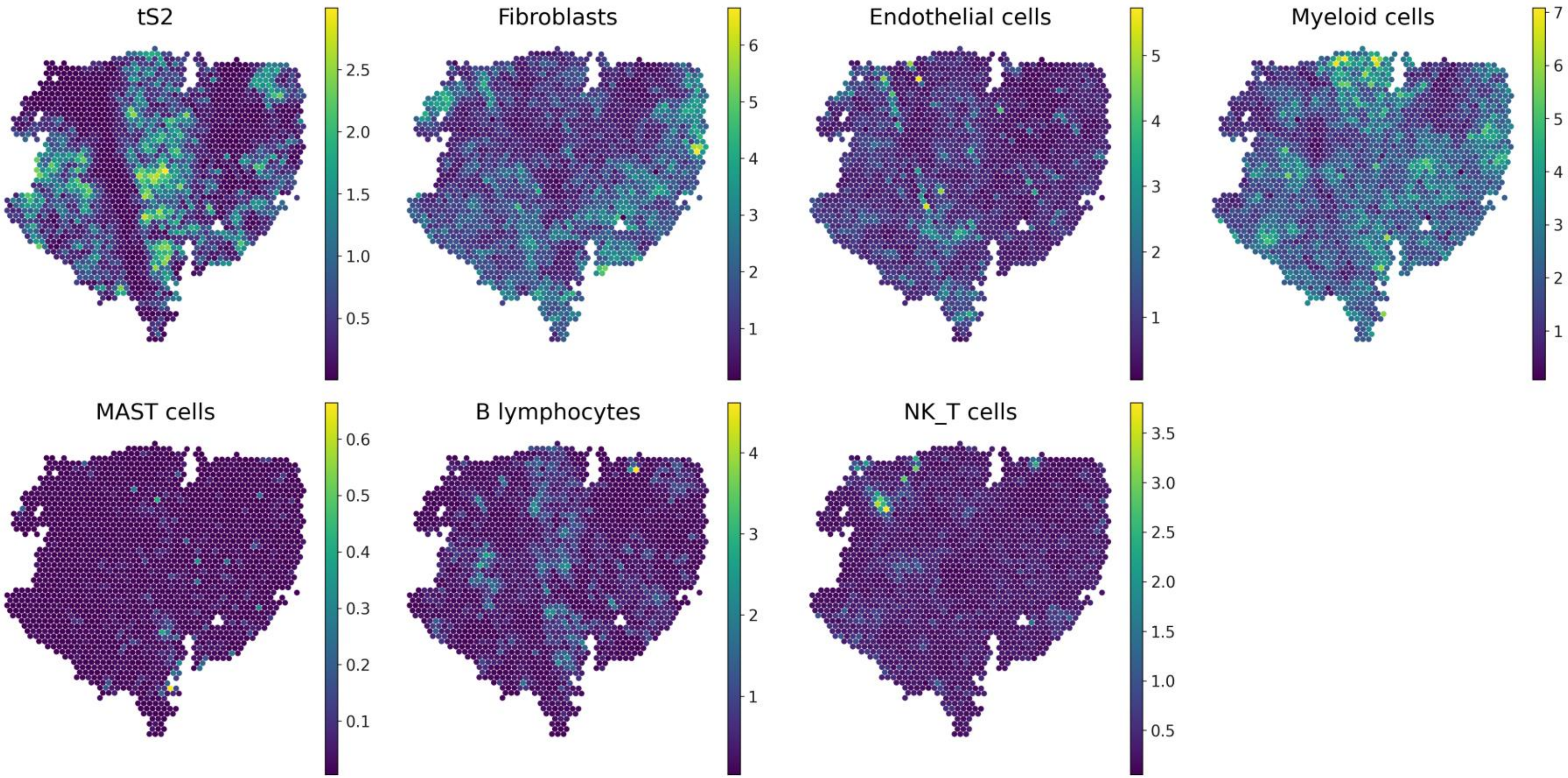

tS2
Fibroblasts
Endothelial cells
Myeloid cells
MAST cells
B lymphocytes
NK_T cells

(c)

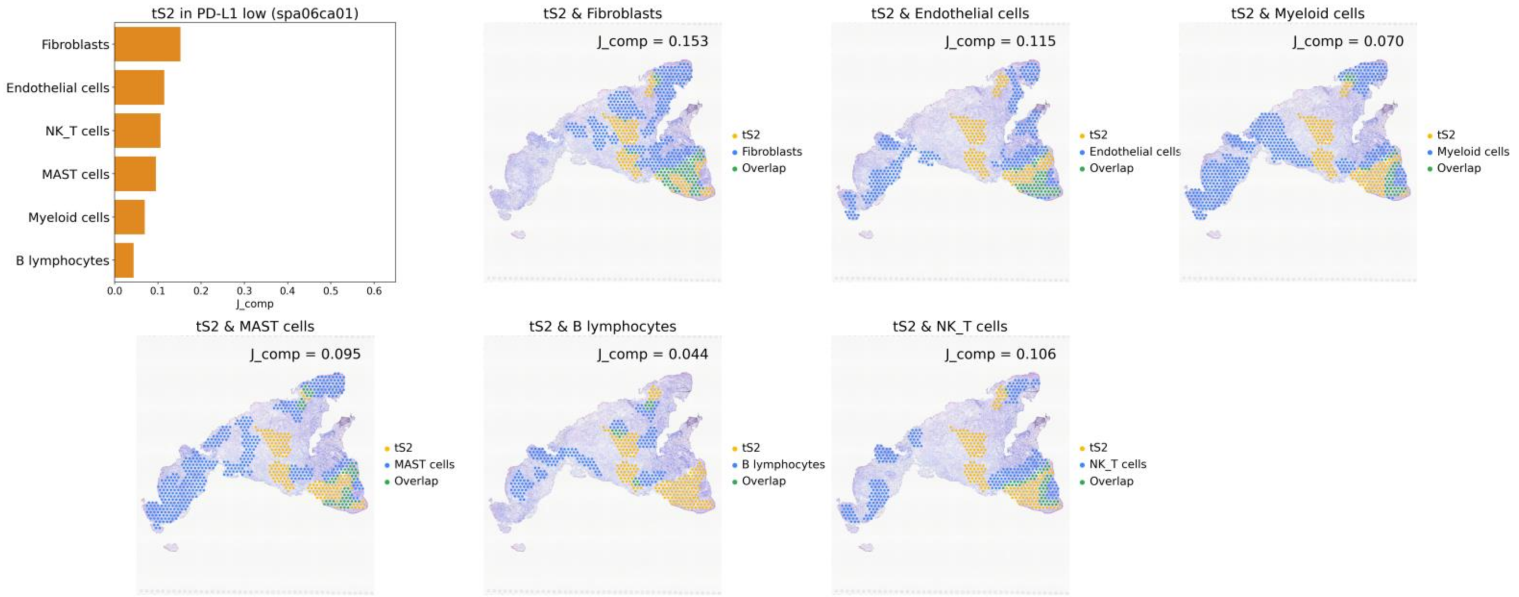

tS2 in PD-L1 low (spa06ca01)
Fibroblasts
Endothelial cells
NK_T cells
MAST cells
Myeloid cells
B lymphocytes
0.0
0.1
0.2
0.3
0.4
0.5
0.6
J_comp
tS2 & Fibroblasts
J_comp = 0.153
tS2
Fibroblasts
Overlap
tS2 & Endothelial cells
J_comp = 0.115
tS2
Endothelial cells
Overlap
tS2 & Myeloid cells
J_comp = 0.070
tS2
Myeloid cells
Overlap
tS2 & MAST cells
J_comp = 0.095
tS2
MAST cells
Overlap
tS2 & B lymphocytes
J_comp = 0.044
tS2
B lymphocytes
Overlap
tS2 & NK_T cells
J_comp = 0.106
tS2
NK_T cells
Overlap

(d)

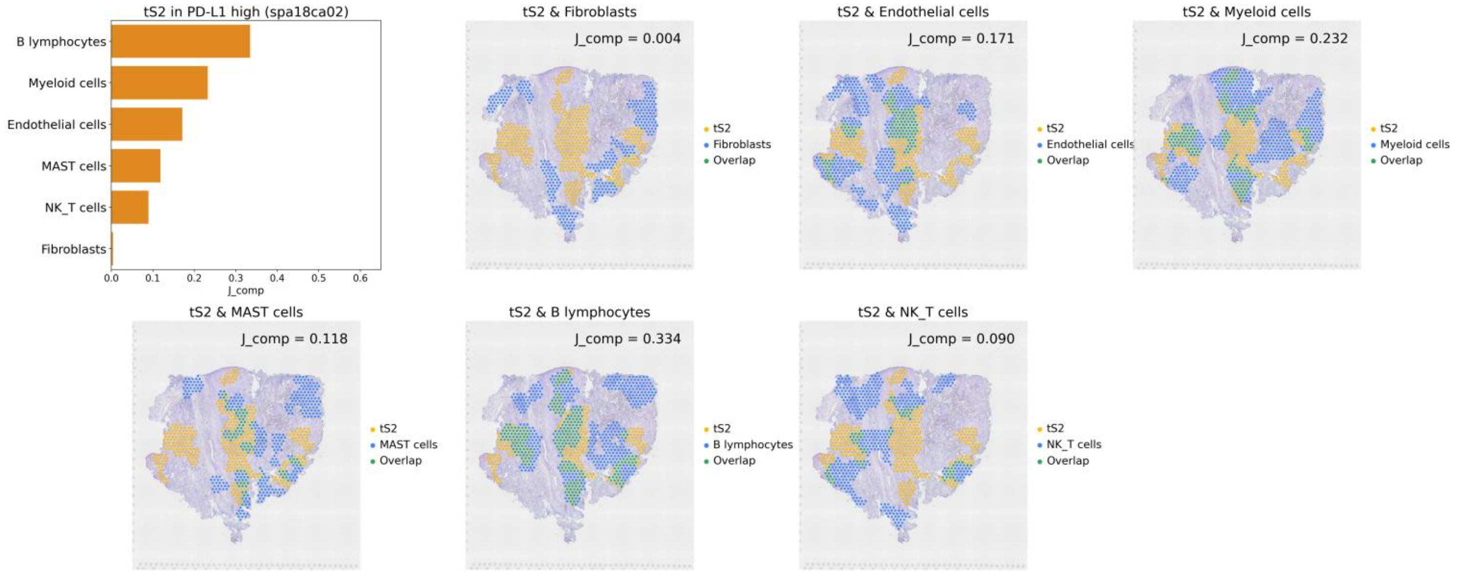

tS2 in PD-L1 high (spa18ca02)
B lymphocytes
Myeloid cells
Endothelial cells
MAST cells
NK_T cells
Fibroblasts
0.0
0.1
0.2
0.3
0.4
0.5
0.6
J_comp
tS2 & Fibroblasts
J_comp = 0.004
tS2
Fibroblasts
Overlap
tS2 & Endothelial cells
J_comp = 0.171
tS2
Endothelial cells
Overlap
tS2 & Myeloid cells
J_comp = 0.232
tS2
Myeloid cells
Overlap
tS2 & MAST cells
J_comp = 0.118
tS2
MAST cells
Overlap
tS2 & B lymphocytes
J_comp = 0.334
tS2
B lymphocytes
Overlap
tS2 & NK_T cells
J_comp = 0.090
tS2
NK_T cells
Overlap

(e)

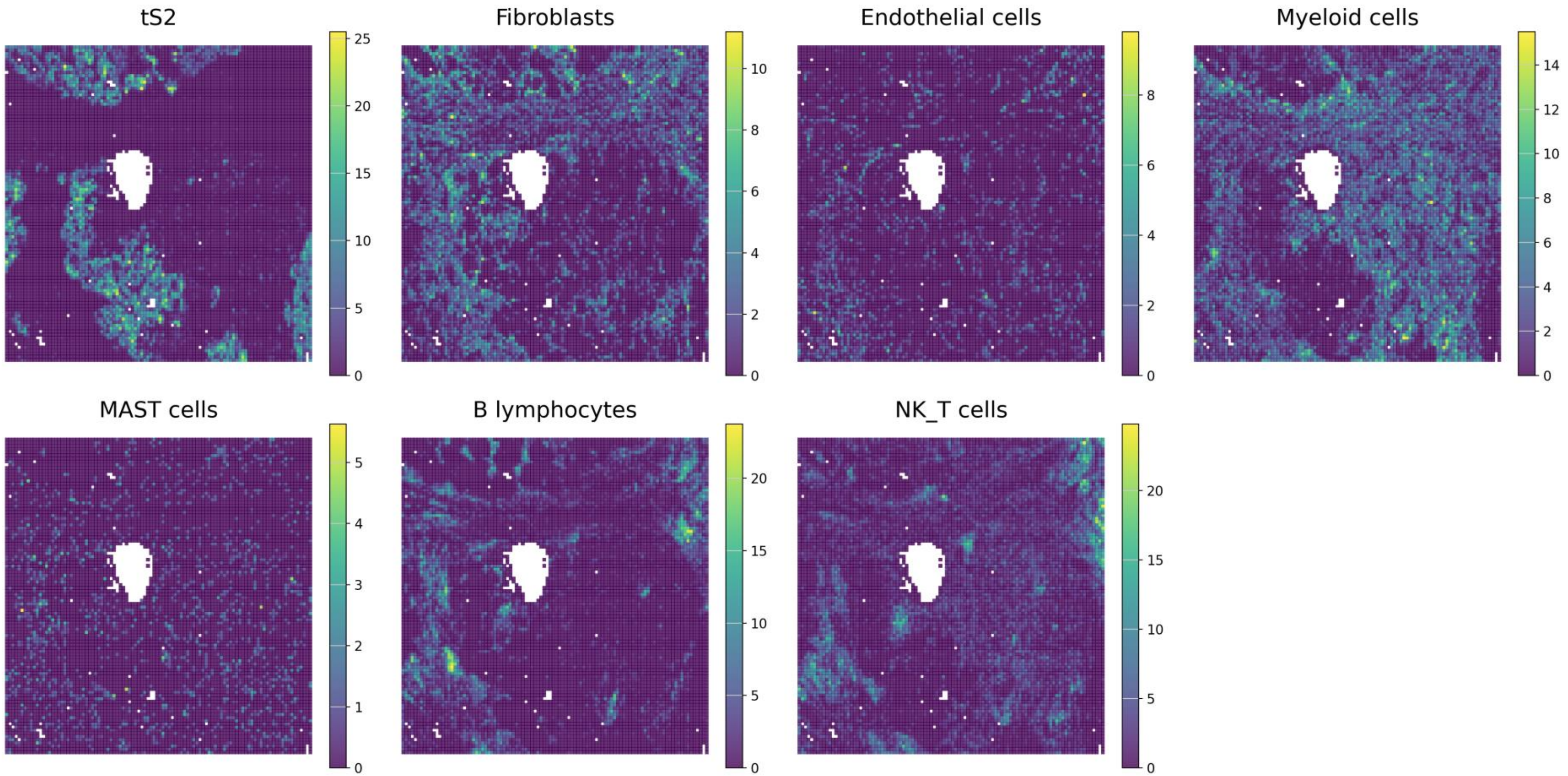

tS2
Fibroblasts
Endothelial cells
Myeloid cells
MAST cells
B lymphocytes
NK_T cells

(f)

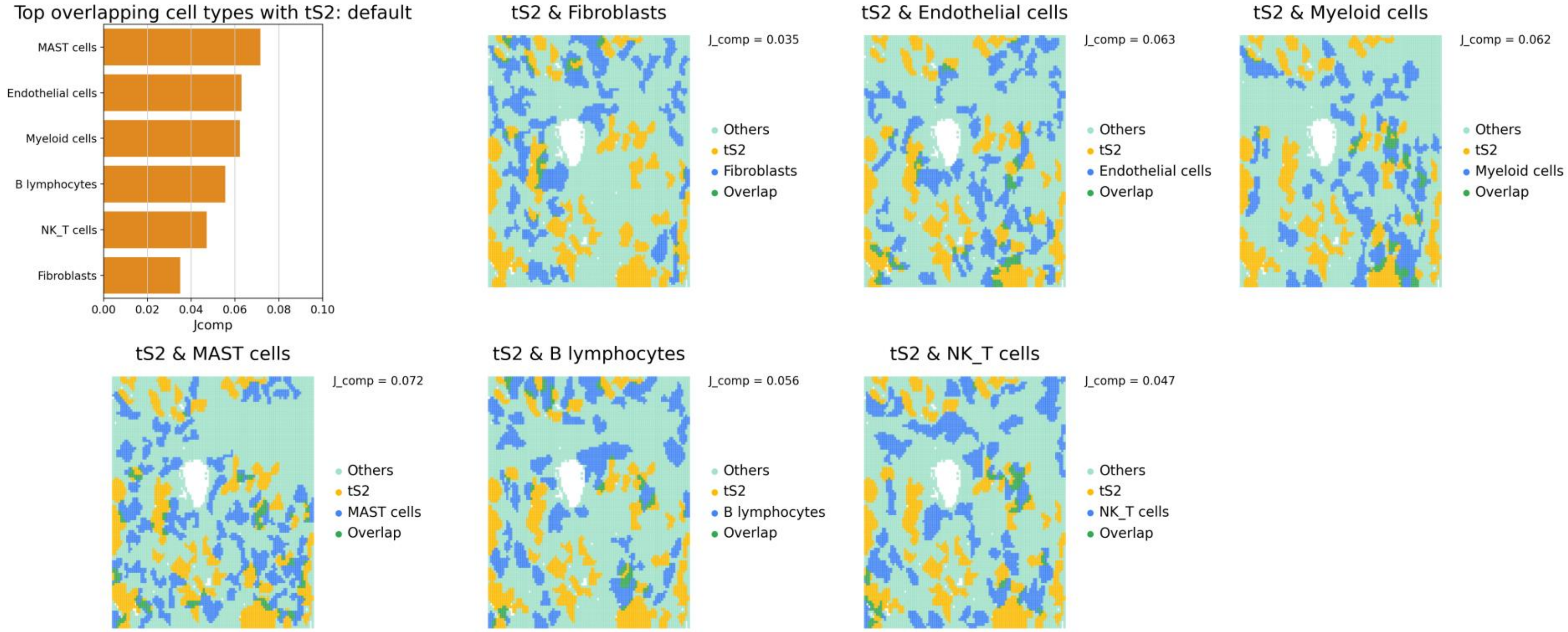
Top overlapping cell types with tS2: default
MAST cells
Endothelial cells
Myeloid cells
B lymphocytes
NK_T cells
Fibroblasts
0.00 0.02 0.04 0.06 0.08 0.10
Jcomp
tS2 & Fibroblasts
J_comp = 0.035
Others
tS2
Fibroblasts
Overlap
tS2 & Endothelial cells
J_comp = 0.063
Others
tS2
Endothelial cells
Overlap
tS2 & Myeloid cells
J_comp = 0.062
Others
tS2
Myeloid cells
Overlap
tS2 & MAST cells
J_comp = 0.072
Others
tS2
MAST cells
Overlap
tS2 & B lymphocytes
J_comp = 0.056
Others
tS2
B lymphocytes
Overlap
tS2 & NK_T cells
J_comp = 0.047
Others
tS2
NK_T cells
Overlap

Supplementary Fig. 24. Colocalization patterns predicted by STopover using different cell type deconvolution and annotation methods

First, the spatial distribution of cell types in barcode-based SRT of lung cancer tissues was predicted using Cell2location, a cell type deconvolution tool. The spatial composition of tumor cell type (tS2) and other main cell types (fibroblasts, endothelial cells, myeloid cells, MAST cells, B lymphocytes, and NK/T cells) in **(a)** PD-L1 low (*spa6ca01*) and **(b)** PD-L1 high tissues (*spa18ca02*) was visualized. Subsequently, STopover was applied to investigate the spatial colocalization patterns between tumor cells (tS2) and other main cell types in both tissues. **(c)** The set-based J_{comp} values between the tumor cell type (tS2) and the other cell types in the spa06ca01 tissue were visualized as a bar plot in the top left corner of the plot. Additionally, the aggregated connected components (CCs) for tS2 (yellow) and other main cell types (blue) were mapped onto the tissue, with the intersecting tissue domain highlighted in green. **(d)** Similarly, the set-based J_{comp} values for tS2 and other cell types in spa18ca02 tissue were visualized with a barplot, and the CC locations were mapped onto the tissue.

Second, cells in the image-based SRT of lung cancer were annotated based on TACCO, a cell type annotation tool. **(e)** The spatial map showing the abundance of tS2 and other main cell types (fibroblasts, endothelial cells, myeloid cells, MAST cells, B lymphocytes, and NK/T cells) was visualized on the transformed grid. Subsequently, STopover was applied to estimate spatial colocalization between tumor cells and other main cell types. **(f)** The set-based J_{comp} scores were calculated between the tumor cell type (tS2) and other main cell types and were visualized with a bar plot in the top left corner. The locations of CCs for tS2 (yellow) and other cell types (blue) were mapped onto the tissue, with the overlapping domain highlighted in green.

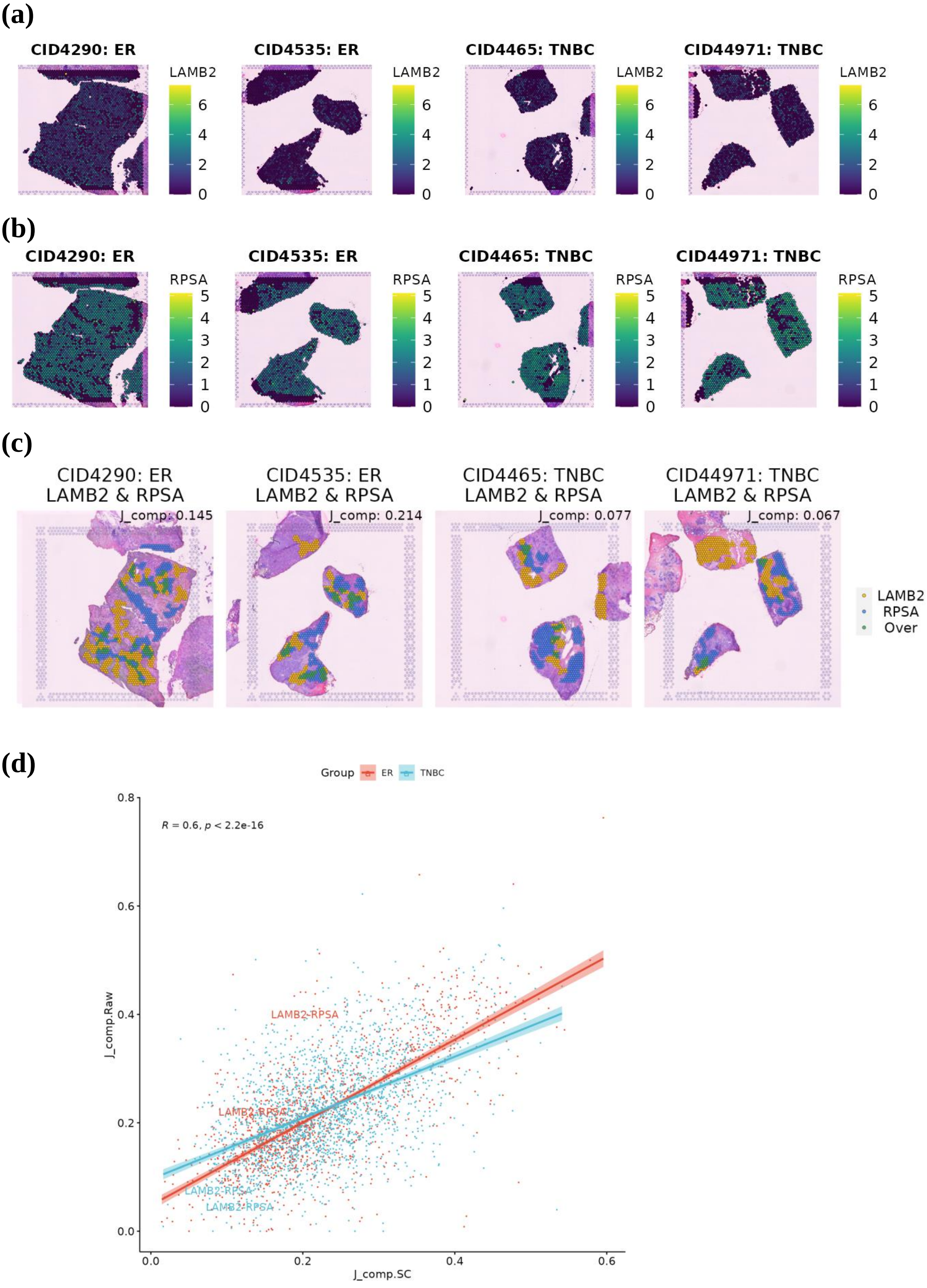
(a)
CID4290: ER
CID4535: ER
CID4465: TNBC
CID44971: TNBC
LAMB2
(b)
CID4290: ER
CID4535: ER
CID4465: TNBC
CID44971: TNBC
RPSA
(c)
CID4290: ER LAMB2 & RPSA
J_comp: 0.145
CID4535: ER LAMB2 & RPSA
J_comp: 0.214
CID4465: TNBC LAMB2 & RPSA
J_comp: 0.077
CID44971: TNBC LAMB2 & RPSA
J_comp: 0.067
LAMB2
RPSA
Over
(d)
Group ER TNBC
R = 0.6, p < 2.2e-16
LAMB2-RPSA
J_comp.Raw
J_comp.SC

Supplementary Fig. 25. STopover analysis before and after correction for RNA diffusion

The tool to correct the lateral diffusion of RNA molecules in barcode-based SRT, called SpotClean, was applied to breast cancer datasets. This correction process was applied to genes with an average expression over 0.1. The spatial expression patterns of the corrected RNA for a ligand, **(a)** *LAMB2*, and receptor, **(b)** *RPSA*, were visualized on the tissue. **(c)** The connected components (CCs) of the *LAMB2* (yellow) and *RPSA* (blue), along with the intersecting subregions (green) between the two aggregated CCs, were visualized on the tissue. The set-based J_{comp} values between cancer epithelial cells and other main cell types in four breast cancer tissues were displayed in the top right corner of the plot. **(d)** The scatter plot illustrates the correlation between J_{comp} values for ligand and receptor pairs in CellTalkDB from four breast cancer tissues before (J_comp.Raw) and after correction (J_comp.SC) for RNA diffusion. The red line and blue line represent linear regression lines for estrogen receptor-positive (ER+) and triple-negative (TNBC) breast cancer, respectively. The annotated text indicates the location of the data points for *LAMB2-RPSA* in the four cancer tissues (ER+: red, TNBC: blue). The Spearman's correlation coefficient is calculated and displayed in the top left corner of the plot.
